# Solvent- and ligand-induced switch of selectivity in gold(I)-catalyzed tandem reactions of 3-propargylindoles

**DOI:** 10.3762/bjoc.7.89

**Published:** 2011-06-09

**Authors:** Estela Álvarez, Delia Miguel, Patricia García-García, Manuel A Fernández-Rodríguez, Félix Rodríguez, Roberto Sanz

**Affiliations:** 1 Área de Química Orgánica, Departamento de Química, Facultad de Ciencias, Universidad de Burgos, Pza. Misael Bañuelos s/n, 09001 Burgos, Spain; 2 Instituto Universitario de Química Organometálica “Enrique Moles”, Universidad de Oviedo, C/Julián Clavería 8, 33006 Oviedo, Spain

**Keywords:** catalysis, gold, indoles, Nazarov cyclizations, selectivity

## Abstract

The selectivity of our previously described gold-catalyzed tandem reaction, 1,2-indole migration followed by aura-iso-Nazarov cyclization, of 3-propargylindoles bearing (hetero)aromatic substituents at both the propargylic and terminal positions, was reversed by the proper choice of the catalyst and the reaction conditions. Thus, 3-(inden-2-yl)indoles, derived from an aura-Nazarov cyclization (instead of an aura-iso-Nazarov cyclization), were obtained in moderate to good yields from a variety of 3-propargylindoles.

## Introduction

**Scheme 1:**
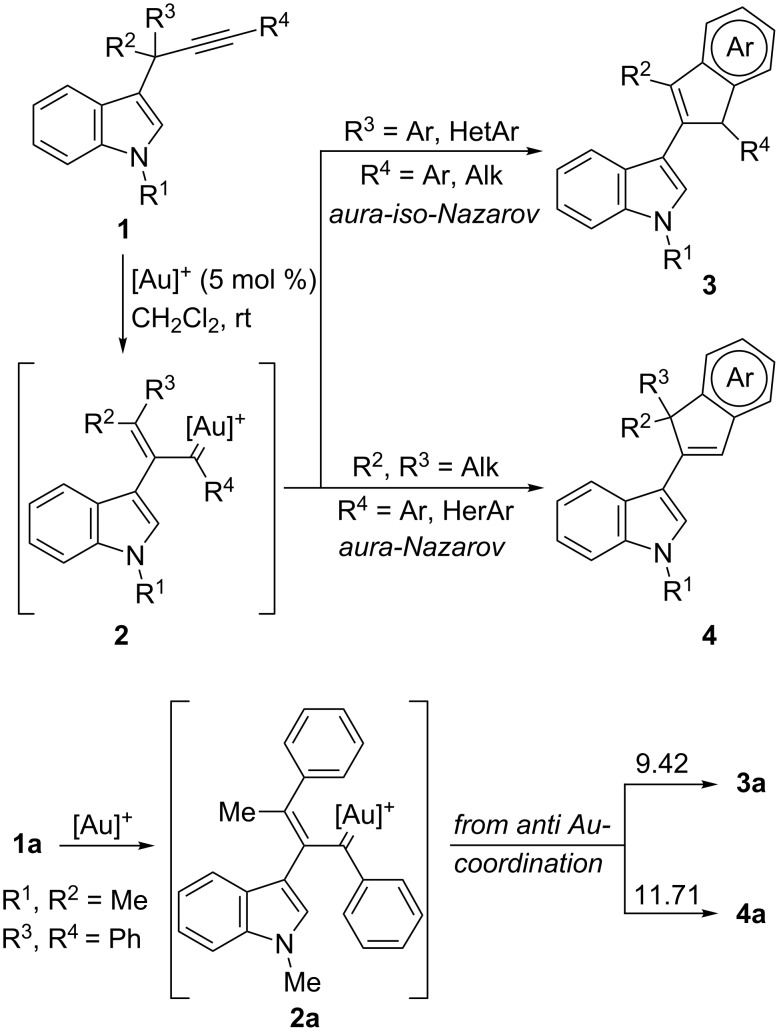
Formation of 3-(inden-2-yl)indoles **3** and **4** from 3-propargylindoles. Energy barriers (kcal/mol) for the cyclization reactions of gold-carbenoid intermediate **2a**.

Catalysis with gold complexes as carbophilic π-acids has become a highly developed area in the last decade [1–7]. In particular, 1,2-acyl migration reactions of propargylic esters have been extensively investigated. In these processes the gold-carbenoid species generated are able to undergo a wide variety of further transformations [8–11]. In addition, propargylic sulfides have also been reported as useful substrates for this type of process, participating in related 1,2-sulfur migrations [12]. Within this area we have reported the first examples of gold-catalyzed migration reactions in propargylic systems that involve a carbon-centered moiety, implying that carbon–carbon bonds are broken and formed instead of carbon–heteroatom bonds [13–14]. Based on the nucleophilic nature of indoles [15], which are known to react with gold-activated alkynes or allenes [16–17], and by taking advantage of our reported methodology for the synthesis of 3-propargylindoles [18–19], we have shown that the indole nucleus is able to participate in gold-catalyzed 1,2-migration reactions of propargylic systems. Thus, 3-propargylindoles **1** give rise to α,β-unsaturated gold-carbenoid intermediates **2** that evolve through different pathways depending on the substituents at the propargylic and terminal positions of the alkyne moiety (Scheme 1). If (hetero)aromatic substituents are present at either of these positions, they undergo further cyclizations to afford 3-(inden-2-yl)indoles **3** or **4** (Scheme 1). An analysis of the aromaticity of the transition state structures for these cyclizations by DFT calculations revealed that these electrocyclic ring closures could be considered as gold variants of the Nazarov (cyclization from **2** to **4**) or iso-Nazarov reactions (cyclization from **2** to **3**) [14]. These theoretical calculations also showed that in those cases where both cyclization pathways are possible (for example in **2a** arising from **1a**; Scheme 1), the calculated energy barriers for the two cyclization modes favored the iso-Nazarov-product **3a** (9.42 kcal/mol vs 11.71 kcal/mol for the Nazarov cyclization assuming that the initial gold coordination to the alkyne is *anti* to the indole). This is in complete agreement with the experimental data, as we always observed the selective formation of cyclization products **3** in those cases where both **3** and **4** could be obtained. However, for the model compound **1a**, similar energy profiles were obtained for the corresponding iso-Nazarov and Nazarov pathways (ΔE = 2.29 kcal/mol) [20]. Since there are several examples reported in the literature that show that the reactivity and selectivity of reactions catalyzed by gold complexes can be appropriately tuned [21–27], we thought that it should be possible to reverse the selectivity of our tandem reaction in favor of the iso-Nazarov pathway, to obtain compounds **4** by a proper setting of the reaction conditions (modulation of the electronic properties of the ligands, counter ion, solvent, substitution pattern of the substrates, etc.). Herein, we report our efforts to control the two competing pathways in the evolution of gold-carbenoid intermediates generated by an initial 1,2-indole migration in 3-propargylindoles.

## Results and Discussion

**Scheme 2:**
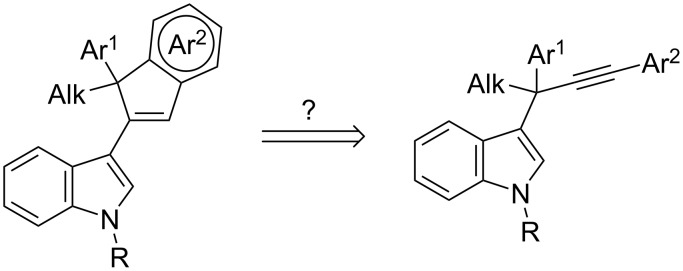
Tandem 1,2-indole migration/aura-Nazarov cyclization from 3-propargylindoles bearing an aromatic substituent at the propargylic position.

It is an intriguing possibility that the aura-Nazarov reaction may also take place with substrates bearing aromatic substituents at both the propargylic and terminal positions, and thus allow access to new functionalized indole derivatives (Scheme 2). It should be remarked that, until now, only the aura-Nazarov cyclization to give products **4** from substrates **1** (without an aromatic substituent at the propargylic position, see R^2^, R^3^ in Scheme 1) has been observed.

**Figure 1:**
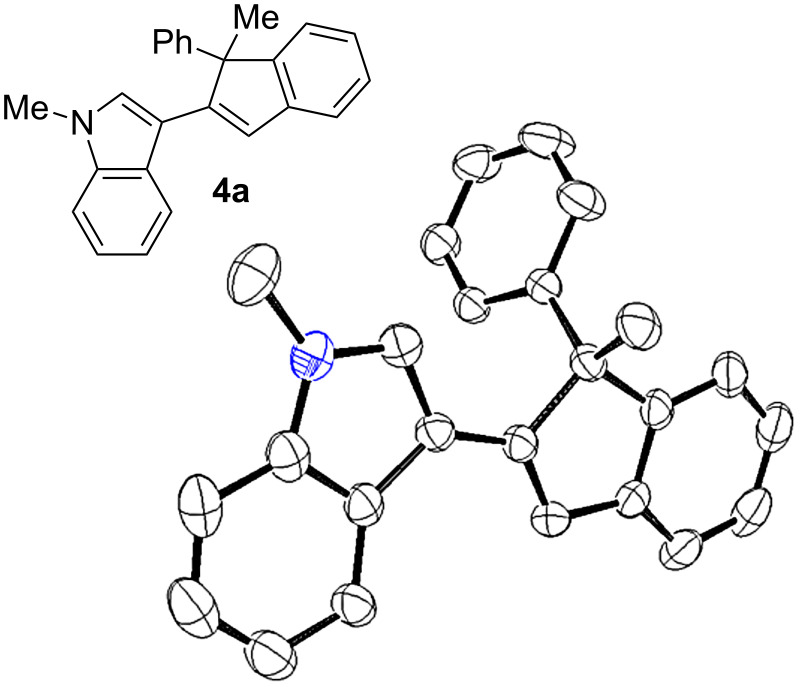
ORTEP diagram for **4a**. Ellipsoids are shown at 30% level (hydrogen atoms are omitted for clarity).

For the initial selectivity control experiments, 1-methyl-3-(1-methyl-1,3-diphenylprop-2-ynyl)-1*H*-indole (**1a**) was selected as the model compound and was treated with several gold catalysts under different reaction conditions (Table 1). As expected, under our standard reported conditions ((Ph_3_P)AuCl/AgSbF_6_ in CH_2_Cl_2_ at room temperature), the 3-(inden-2-yl)indole **3a** was obtained as the major product. However, a minor isomer **4a** was also isolated along with **3a** in a ca. 3.5/1 ratio (Table 1, entry 1). The structure of the minor compound **4a** was established by X-ray diffraction (Figure 1), confirming that the gold-carbenoid intermediate **2a** could also undergo the aura-Nazarov cyclization [28–29].

**Table 1:** Effect of the catalyst and reaction conditions on the reactivity of **1a**.^a^

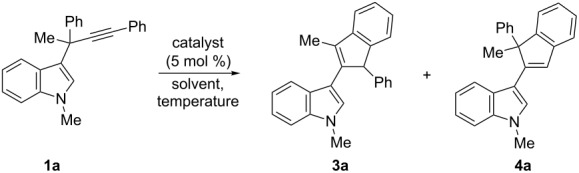			

Entry	Catalyst	Solvent	Ratio^b^ **3a**/**4a**

1	(Ph_3_P)AuCl/AgSbF_6_	CH_2_Cl_2_	3.5/1
2	(Ph_3_P)AuNTf_2_	CH_2_Cl_2_	3.3/1
3	SPhosAuNTf_2_^c^	CH_2_Cl_2_	2.5/1
4	(Et_3_P)AuCl/AgSbF_6_	CH_2_Cl_2_	3.3/1^d^
5	IMeAuCl^e^/AgSbF_6_	CH_2_Cl_2_	2.5/1
6	IPrAuCl^f^/AgSbF_6_	CH_2_Cl_2_	3/1
7	[(PhO)_3_P]AuCl/AgSbF_6_	CH_2_Cl_2_	2.2/1
8	[(2,4-(*t*-Bu)_2_C_6_H_3_O)_3_P]AuCl/AgSbF_6_	CH_2_Cl_2_	1.5/1
9	[(2,4-(*t*-Bu)_2_C_6_H_3_O)_3_P]AuCl/AgBF_4_	CH_2_Cl_2_	1.5/1
10	[(2,4-(*t*-Bu)_2_C_6_H_3_O)_3_P]AuCl/AgNTf_2_	CH_2_Cl_2_	2/1
11	[(2,4-(*t*-Bu)_2_C_6_H_3_O)_3_P]AuCl/AgOTs	CH_2_Cl_2_	1.5/1
12	[(2,4-(*t*-Bu)_2_C_6_H_3_O)_3_P]AuCl/AgOTf	CH_2_Cl_2_	1.4/1
13	[(2,4-(*t*-Bu)_2_C_6_H_3_O)_3_P]AuCl/AgSbF_6_	DME	1.4/1
14	[(2,4-(*t*-Bu)_2_C_6_H_3_O)_3_P]AuCl/AgSbF_6_	THF	1.4/1
15	[(2,4-(*t*-Bu)_2_C_6_H_3_O)_3_P]AuCl/AgSbF_6_	toluene	1/1.8
16	[(2,4-(*t*-Bu)_2_C_6_H_3_O)_3_P]AuCl/AgSbF_6_	toluene^g^	1/2.3
17	[(2,4-(*t*-Bu)_2_C_6_H_3_O)_3_P]AuCl/AgSbF_6_	toluene^h^	1/4^i^

^a^Reactions carried out until complete consumption of the starting material **1a**, as judged by GC-MS and/or TLC analysis, unless otherwise stated. ^b^Determined by ^1^H NMR analysis of the crude reaction mixture. ^c^SPhos = 2-dicyclohexylphosphino-2´,6´-dimethoxybiphenyl. ^d^66% of conversion after 24 h. ^e^IMe = 1,3,4,5-tetramethylimidazol-2-ylidene. ^f^IPr = 1,3-bis(2,6-di-isopropylphenyl)imidazol-2-ylidene. ^g^Conducted at 0 °C. A similar result was obtained by using AgOTf as a silver salt. ^h^Carried out at −20 °C. ^i^50% conversion after 24 h.

The use of cationic gold complexes bearing different types of phosphane ligands always provided the iso-Nazarov product **3a** as the major isomer, with a small increase in the competing Nazarov product **4a** on switching the ligand to SPhos (Table 1, entries 1–4). The use of complexes bearing N-heterocyclic carbene ligands [30] also produced **3a** as the major compound of the corresponding mixtures (Table 1, entries 5 and 6). It was decided to increase the π-acceptor character of the ligand [31], and, in this case, the employment of a triphenylphosphite–gold(I) complex led to a slight increase in the ratio of **4a** (Table 1, entry 7). Finally, the use of the bulky phosphite ligand tris(2,4-di-*t*-butylphenyl)phosphite, gave rise to a 1.5/1 ratio of **3a**/**4a** (Table 1, entry 8) [32].

Once tris(2,4-di-*t*-butylphenyl)phosphite was selected as the best ligand to favor the desired tandem process, the influence of the metal counter ion was then studied. Thus, several silver salts were employed for the generation of the cationic catalytic active gold(I) complex, and it was concluded that the effect on the selectivity is almost negligible (Table 1, entries 9–12). Nevertheless, it should be noted that no reaction occurred when AgOBz was employed whilst the reactions with AgBF_4_, AgNTf_2_ and AgOTs were relatively slow. Therefore, AgOTf and AgSbF_6_ were selected as silver salts, due to their availability and higher reactivity, and subsequently the effect of the solvent was studied. Ethereal solvents, such as DME and THF, led to similar results as CH_2_Cl_2_ (Table 1, entries 13 and 14), whereas acetonitrile proved to be unsuitable for the reaction. Gratifyingly, it was found that the use of toluene as the solvent reverses the selectivity of the reaction, and, with this solvent, the Nazarov product **4a** became the major isomer in the mixture (Table 1, entry 15). Finally, the effect of the temperature in toluene was investigated: It was found that carrying out the reaction at 0 °C afforded a 2.3/1 ratio of isomers in favor of **4a** (Table 1, entry 16). If the temperature is lowered to −20 °C the ratio in favor of **4a** was even higher, although only a 50% conversion was observed after 24 h (Table 1, entry 17). Under the optimized and synthetically useful reaction conditions, i.e., toluene at 0 °C, with [(2,4-(*t*-Bu)_2_C_6_H_3_O)_3_P]AuCl/AgOTf as the catalytic system, **4a** was obtained in 60% isolated yield.

At this point it was unclear whether the observed change of selectivity in favor of the Nazarov product **4a** was mainly a solvent effect, or if the nature of the ligand also exerted an influence on the selectivity. To clear up this point the initial catalyst [(Ph_3_P)AuCl/AgOTf] was revisited, and **1a** was treated with this catalytic system in toluene. Since the reaction was very slow at 0 °C, the temperature was increased to rt. Under these conditions the observed **3a**/**4a** ratio was 1/1.5. By comparing this result with that in entry 1 of Table 1 led to the conclusion that the change of solvent is the main factor responsible for the selectivity switch in favor of the Nazarov product. Nevertheless, the beneficial effect of the bulky phosphite ligand is also significant factor with regards to both reactivity and selectivity.

To examine further the scope of this switch of selectivity in favor of the Nazarov pathway in tandem gold-catalyzed reactions of 3-propargylindoles initiated by 1,2-indole migrations, a selection of substrates **1a**–**i**, bearing a methyl group at one of the propargylic positions and different (hetero)aromatic groups at both the other propargylic and terminal positions, were reacted under the established conditions (Table 2). From the results obtained, the selectivity in favor of the Nazarov products **4** seems to be general for the selected indoles **1a**–**h** (Table 2, entries 1–8). N-unsubstituted indole **1b** also showed a preference for the corresponding Nazarov product **4b**, although in this case the selectivity was slightly lower compared to **1a** (Table 2, entry 2), and the reaction gave a poorer overall yield. When an electron-withdrawing substituent was present on the aryl group at the propargylic position, selectivity in favor of Nazarov products **4** appeared to be slightly increased (Table 2, entry 3) [33]. Substrate **1d**, with a bulky electron-withdrawing substituent at one of the *ortho* positions of the aromatic propargylic group, afforded almost exclusively the Nazarov product **4d** in high yield (Table 2, entry 4). Similarly, the presence of a π-electron rich heteroaromatic group or an electron-rich aromatic group at the terminal position of the triple bond also favors the Nazarov pathway (Table 2, entries 5–7). On the other hand, the use of 3-propargylindole **1h** as starting material, bearing an electron-withdrawing substituent on the aromatic ring at the terminal position, led to a slight decrease in the selectivity (Table 2, entry 8). Moreover, the introduction of an electron-donating group on the aromatic ring at the propargylic position gave rise to the almost exclusive formation of the iso-Nazarov product **3i** (Table 2, entry 9). A comparison of these selectivities with that obtained for the parent indole **1a**, leads to the conclusion that the electronic nature of the aryl groups at both the propargylic and terminal positions also has a significant influence on the preferred cyclization pathway. The Nazarov products **4** seem to be more favored when electron-withdrawing groups are present at the propargylic position and electron-donating substituents are present at the terminal position. Under these optimized conditions, new and interesting 3-(inden-2-yl)indoles **4a**–**h** were isolated in good yields.

**Table 2:** Synthesis of 3-(inden-2-yl)indoles **4** by gold-catalyzed tandem 1,2-indole migration/Nazarov-type cyclization of 3-propargylindoles **1**.

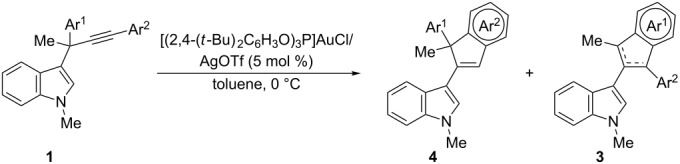				

Entry	Substrate	Ratio Nazarov (**4**)/ iso-Nazarov (**3**)^a^	Product	Yield (%)^b^

1	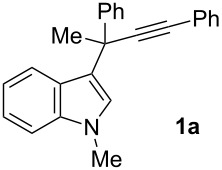	2.3/1	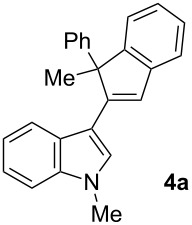	60
2	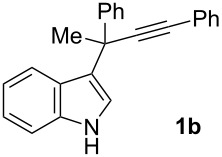	1.8/1	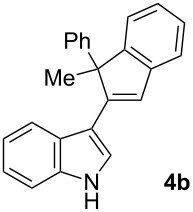	41
3	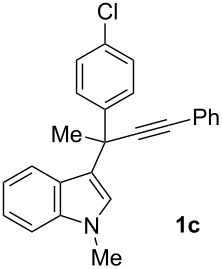	3/1	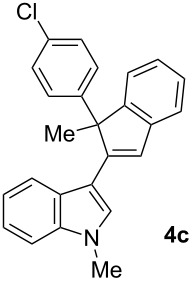	67
4	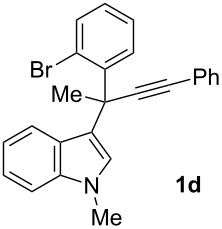	>10/1	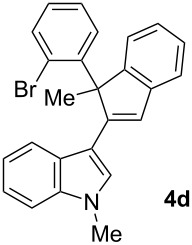	86
5	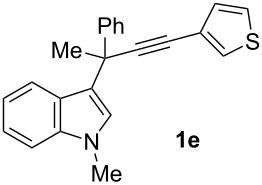	3/1	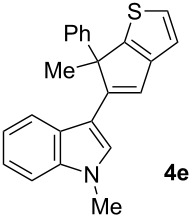	62
6	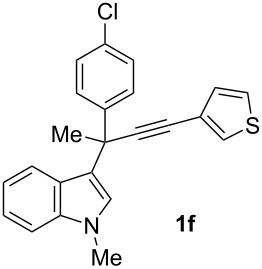	4/1	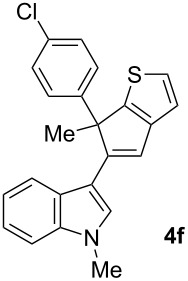	71
7	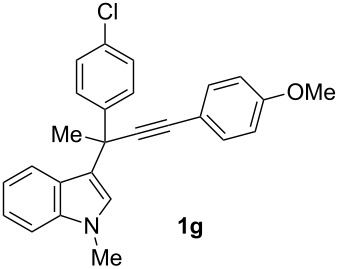	3/1	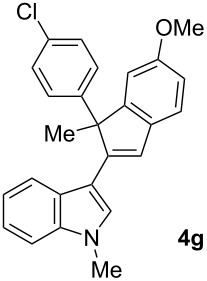	60
8	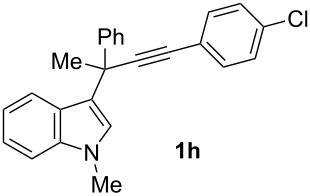	1.2/1	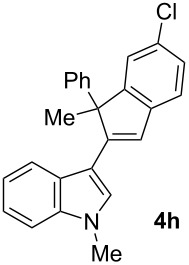	47^c^
9	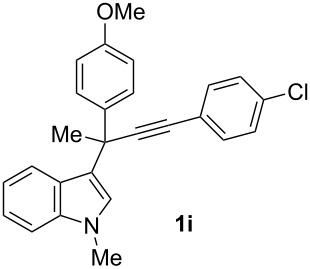	<1/10	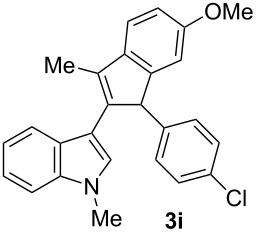	88^d^

^a^Determined by ^1^H NMR analysis of the crude reaction mixture. ^b^Isolated yield of compounds **4** after column chromatography, unless otherwise stated. ^c^Determined by NMR from the mixture of **3h** and **4h**.^d^Combined yield for **3i** and **3´i**, in which the double bond has isomerized. Both compounds have been isolated and characterized. See Supporting Information File 1 (Experimental and analytical data) and Supporting Information File 2 (NMR spectra).

**Scheme 3:**
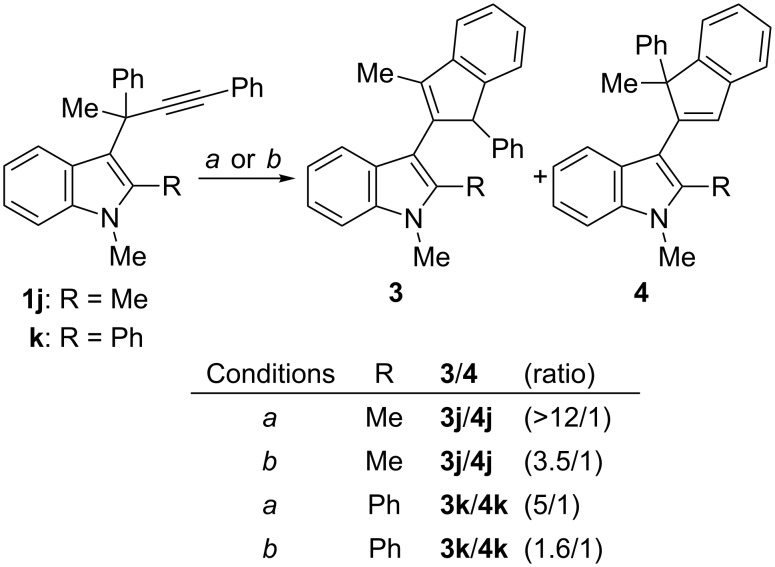
Comparison of the reactivity of C-2 substituted indoles **1j** and **1k**. Conditions: *a*) (Ph_3_P)AuCl/AgSbF_6_ (5 mol %), CH_2_Cl_2_, rt; *b*) [(2,4-(*t*-Bu)_2_C_6_H_3_O)_3_P]AuCl/AgOTf (5 mol %), toluene, 0 °C.

By contrast, it was previously observed that substitution at C-2 of the starting 3-propargylindole led almost exclusively to the formation of iso-Nazarov products **3** [13–14]. For instance, indoles **1j** and **1k**, bearing a methyl and a phenyl group at C-2, respectively, provided the corresponding indole derivatives **3j** and **3k** with high selectivity when the reaction was conducted in CH_2_Cl_2_ with (Ph_3_P)AuCl/AgSbF_6_ as catalyst (Scheme 3). Interestingly, under the new conditions developed herein, i.e., treatment with a cationic phosphite–gold complex in toluene, the reaction of **1j** afforded a ca. 3.5/1 mixture of **3j**/**4j**, whereas **1k** gave rise to a ca. 1.6/1 mixture of **3k**/**4k** (Scheme 3). These results again show that the change of selectivity in the competitive iso-Nazarov/Nazarov pathways could be induced by a change of ligand and solvent, although complete reversal of selectivity was not achieved for these substrates.

**Scheme 4:**
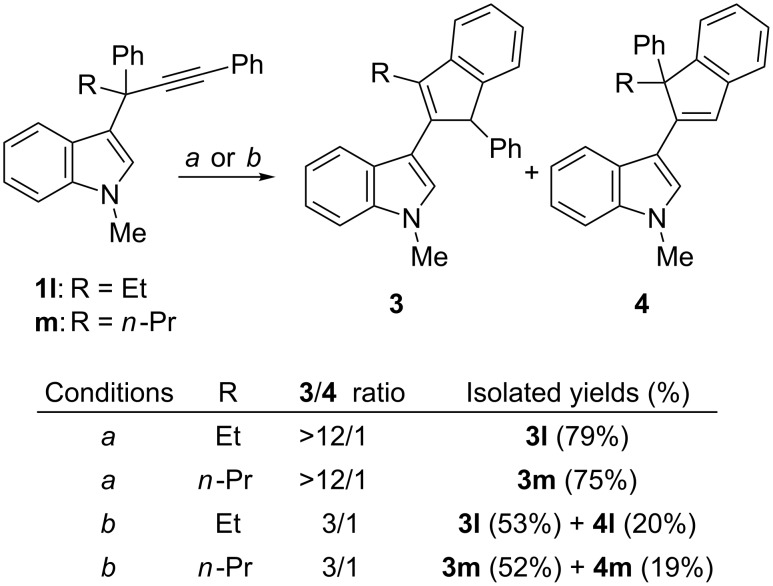
Reactions of 3-propargylindoles **1l** and **1m** with bulky alkyl substituents at the propargylic position. Conditions: *a*) (Ph_3_P)AuCl/AgSbF_6_ (5 mol %), CH_2_Cl_2_, rt; *b*) [(2,4-(*t*-Bu)_2_C_6_H_3_O)_3_P]AuCl/AgOTf (5 mol %), toluene, 0 °C.

It has also been observed that reactions of 3-propargylindoles bearing alkyl substituents bulkier than methyl at the propargylic position, such as **1l** and **1m**, almost exclusively produced the corresponding iso-Nazarov products **3l** and **3m** with (Ph_3_P)AuCl/AgSbF_6_ as catalyst in CH_2_Cl_2_ (Scheme 4) [13–14]. Again, the use of the phosphite–gold complex as catalyst and toluene as solvent slightly favored the Nazarov pathway: Approximately 3/1 ratios of the corresponding indole derivatives **3l, m**/**4l, m** were obtained (Scheme 4) [32]. In addition, we were able to isolate the new Nazarov compounds **4l** and **4m**, albeit in low yields (Scheme 4). Finally, when the more sterically demanding isopropyl group was present at the propargylic position, the corresponding iso-Nazarov product was produced exclusively irrespective of the conditions employed. Although these results show that the change of the methyl group at the propargylic position of the starting indole **1** to a bulkier alkyl group strongly favors the iso-Nazarov pathway, they also show that our new reported conditions make the Nazarov pathway more accessible.

## Conclusion

We have studied the effect of the ligands and counter ion of the catalyst, as well as the electronic nature of the aryl substituents and the reaction conditions (solvent, temperature), in the gold(I)-catalyzed tandem reactions of 3-propargylindoles initiated by 1,2-indole migrations. We have been able to switch the preference of 3-propargylindoles, bearing (hetero)aromatic substituents at both propargylic and terminal positions of the alkyne moiety, from undergoing an aura-iso-Nazarov cyclization in favor of an aura-Nazarov cyclization. The two competitive pathways are influenced mainly by the electronic and steric properties of the aryl substituent at the propargylic position, as well as the ligand of the catalyst and the solvent used. In this way, new and interesting 3-(inden-2-yl)indoles were obtained in good yields.

## Supporting Information

Experimental procedures and spectroscopic data for all new compounds. Copies of ^1^H NMR and ^13^C NMR spectra for new compounds.

File 1 Experimental and analytical data.

File 2 NMR spectra.
