# Supplementary material for: Solvent- and ligand-induced switch of selectivity in gold(I)-catalyzed tandem reactions of 3-propargylindoles
Source: Beilstein J Org Chem. 2011 Jun 9;7:786–93. doi: 10.3762/bjoc.7.89 (PMC3135218; doi:10.3762/bjoc.7.89)
Supplement: File 1 — Experimental and analytical data. [file Beilstein_J_Org_Chem-07-786-s001.pdf]

Supporting Information

for

Solvent- and ligand-induced switch of selectivity in gold(I)-catalyzed tandem reactions of 3-propargylindoles

Estela Álvarez[1], Delia Miguel[1], Patricia García-García[1], Manuel A. Fernández-Rodríguez[1], Félix Rodríguez[2] and Roberto Sanz*[1]

Address: [1]Área de Química Orgánica, Departamento de Química, Facultad de Ciencias, Universidad de Burgos, Pza. Misael Bañuelos s/n, 09001 Burgos, Spain and [2]Instituto Universitario de Química Organometálica "Enrique Moles", Universidad de Oviedo, C/Julián Clavería 8, 33006 Oviedo, Spain

Email: Roberto Sanz - rsd@ubu.es

*Corresponding author

Experimental and analytical data

General procedure 1 (GP1) for the synthesis of starting 3-propargylindoles 1: *p*-Toluenesulfonic acid (5 mol %) was added to a mixture of the corresponding alkynol (1.2 equiv) and *N*-methylindole (1 equiv) in analytical-grade MeCN (~1 mL/mmol). The reaction mixture was stirred at room temperature until the starting *N*-methylindole had been consumed, as determined by GC-MS and/or TLC. The crude reaction mixture was neutralized by the addition of 1 M NaOH. The mixture was extracted with Et_2O (3 × 10 mL) and the combined organic layers were dried with anhydrous Na_2SO_4. The solvent was removed under reduced pressure and the residue purified by silica gel column chromatography (eluent: mixtures of hexane/Et_2O or hexane/AcOEt) to afford the corresponding 3-alkylated indoles **1**. Analytical data for compounds **1a-e** and **1j-n** have been previously described by us [1].

3-[1-(4-Chlorophenyl)-1-methyl-3-thiophen-3-yl-prop-2-ynyl]-1-methyl-1*H*-indole (1f)

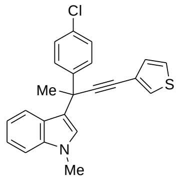

Following **GP1** from 2-(4-chlorophenyl)-4-thiophen-3-yl-but-3-yn-2-ol (1.068 g, 4 mmol) as the alkynol and purification by column chromatography (hexane/AcOEt, 10:1), **1f** was obtained as a pale brown solid (1.101 g, 89%). R_f 0.18 (hexane/AcOEt, 5:1). M.p. 50–52 ºC. ^{1}H NMR (400 MHz, $CDCl_3$): δ 2.13 (s, 3H, CH_3C), 3.80 (s, 3H, CH_3N), 7.01–7.09 (m, 2H, ArH), 7.14–7.17 (m, 1H, ArH), 7.22–7.36 (m, 5H, ArH), 7.42–7.45 (m, 1H, ArH), 7.49–7.51 (m, 1H, ArH), 7.56–7.61 (m, 2H, ArH) ppm. ^{13}C NMR (75 MHz, CDCl3) δ 31.1 (CH_3), 32.9 (CH_3), 39.6 (C), 78.4 (C), 94.1 (C), 109.4 (CH), 119.1 (CH), 119.6 (C), 121.1 (CH), 121.8 (CH), 122.6 (C), 125.1 (CH), 126.0 (C), 126.3 (CH), 182.2 (CH), 182.2 (2 × CH), 182.3 (2 × CH), 130.2 (CH), 132.3 (C), 137.9 (C), 145.0 (C) ppm. LRMS (70 eV, EI): *m/z* (%) 377 [$(M+2)^+$, 17], 375 (M^+, 41),

362 (37), 360 (100), 340 (23), 264 (12). HRMS calcd for $C_{23}H_{18}ClNS$, 375.0848; found, 375.0850.

3-[1-(4-Chlorophenyl)-3-(4-methoxyphenyl)-1-methylprop-2-ynyl]-1-methyl-1*H*-indole (1g)

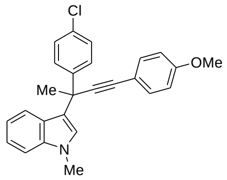

Following **GP1** from 2-(4-chlorophenyl)-4-(4-methoxyphenyl)-but-3-yn-2-ol (1.424 g, 4.9 mmol) as the alkynol and purification by column chromatography (hexane/AcOEt, 20:1), **1g** was obtained as a pale yellow solid (1.086 g, 72%). R_f 0.11 (hexane/AcOEt, 20:1). M.p. 58–60 ºC. ^{1}H NMR (400 MHz, $CDCl_3$): δ 2.08 (s, 3H, CH_3C), 3.78 (s, 3H, XCH_3), 3.79 (s, 3H, XCH_3), 6.80–6.86 (m, 2H, ArH), 6.97–7.03 (m, 1H, ArH), 7.03–7.07 (m, 1H, ArH), 7.17–7.24 (m, 1H, ArH), 7.24–7.34 (m, 3H, ArH), 7.35–7.42 (m, 2H, ArH), 7.45–7.51 (m, 1H, ArH), 7.52–7.59 (m, 2H, ArH) ppm. ^{13}C NMR (75.4 MHz, $CDCl_3$): δ 31.2 (CH_3), 32.8 (CH_3), 39.5 (C), 55.3 (CH_3), 83.1 (C), 93.0 (C), 109.4 (CH), 113.8 (2 × CH), 115.8 (C), 119.0 (CH), 119.8 (C), 121.1 (CH), 121.8 (CH), 126.0 (C), 126.2 (CH), 128.2 (2 × CH), 128.2 (2 × CH), 132.1 (C), 133.1 (2 × CH) 137.9 (C), 145.3 (C), 159.3 (C) ppm. LRMS (70 eV, EI): *m/z* (%) 401 [$(M+2)^+$, 24], 399 (M^+, 66), 386 (36), 384 (100), 364 (28), 288 (10). HRMS calcd for $C_{26}H_{22}ClNO$, 399.1390; found, 399.1382.

3-[4-(4-Chlorophenyl)-2-phenylbut-3-yn-2-yl]-1-methyl-1*H*-indole (1h)

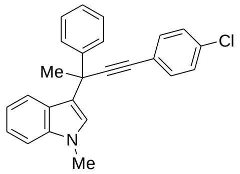

Following **GP1** from 4-(4-chlorophenyl)-2-phenylbut-3-yn-2-ol (0.150 g, 0.58 mmol) as the alkynol and purification by column chromatography (hexane/AcOEt, 20:1), **1h** was obtained as a white solid (0.145 g, 80%). R_f 0.25 (hexane/AcOEt, 10:1). M.p. 122–124 ºC. ^{1}H NMR (300 MHz, $CDCl_3$): δ 2.13 (s, 3H CH_3C), 3.80 (s, 3H, NCH_3),

6.96–7.07 (m, 2H, ArH), 7.17–7.46 (m, 9H, ArH), 7.47–7.55 (m, 1H, ArH), 7.58–7.66 (m, 2H, ArH) ppm. ^{13}C NMR (75.4 MHz, $CDCl_3$): δ 31.1 (CH_3), 32.9 (CH_3), 40.00 (C), 82.0 (C), 96.3 (C), 109.4 (CH), 119.0 (CH), 120.0 (C), 121.2 (CH), 121.8 (CH), 122.5 (C), 126.2 (C), 126.3 (CH), 126.6 (CH), 126.7 (2 × CH), 128.3 (2 × CH), 128.5 (2 × CH), 133.0 (2 × CH) 133.7 (C), 137.9 (C), 146.1 (C) ppm. LRMS (70 eV, EI): *m/z* (%) 371 [$(M+2)^+$, 19], 369 (M^+, 53), 356 (36), 354 (100), 292 (10), 241 (12). HRMS calcd for $C_{25}H_{20}ClN$, 369.1284; found, 369.1280.

3-[3-(4-Chlorophenyl)-1-(4-methoxyphenyl)-1-methylprop-2-ynyl)-1-methyl-1*H*-indole (1i)

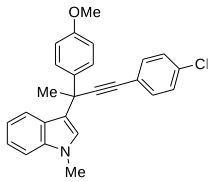

Following **GP1** from 4-(4-chlorophenyl)-2-(4-methoxyphenyl)-but-3-yn-2-ol (2.148 g, 7.5 mmol) as the alkynol and purification by column chromatography (hexane/AcOEt, 10:1), **1i** was obtained as a pale orange solid (1.825 g, 74%). R_f 0.16 (hexane/AcOEt, 20:1). M.p. 60–62 ºC. ^{1}H NMR (400 MHz, $CDCl_3$): δ 2.10 (s, 3H, CH_3C), 3.77 (s, 3H, XCH_3), 3.79 (s, 3H, XCH_3), 6.83–6.90 (m, 2H, ArH), 6.97–7.06 (m, 2H, ArH), 7.17–7.34 (m, 4H, ArH), 7.34–7.42 (m, 2H, ArH), 7.48–7.56 (m, 3H, ArH) ppm. ^{13}C NMR (75.4 MHz, $CDCl_3$): δ 31.2 (CH_3), 32.9 (CH_3), 39.3 (C), 55.3 (CH_3), 81.7 (C), 96.5 (C), 109.4 (CH), 113.5 (2 × CH), 119.0 (CH), 120.3 (C), 121.3 (CH), 121.7 (CH), 122.5 (C), 126.1 (C), 126.3 (CH), 127.8 (2 × CH), 128.5 (2 × CH), 133.0 (2 × CH), 133.7 (C), 137,9 (C), 138.3 (C), 158.2 (C) ppm. LRMS (70 eV, EI): *m/z* (%) 401 [$(M+2)^+$, 29], 399 (M^+, 81), 386 (36), 384 (100), 340 (11). HRMS calcd for $C_{26}H_{22}ClNO$, 399.1390; found, 399.1390.

General procedure 2 (GP2) for the standard gold-catalyzed reaction of 3-propargylindoles 1. Synthesis of indole derivatives 3a and 3c: To a stirred mixture of $(Ph_3P)AuCl$ (12.4 mg, 0.025 mmol, 5 mol%) and $AgSbF_6$ (8.6 mg, 0.025 mmol, 5 mol%) in analytical grade CH_2Cl_2 (1 mL), the corresponding 3-propargylindole **1a,c** (0.5 mmol) was added at rt. The resulting reaction mixture was stirred at rt until complete conversion to the corresponding 3-(inden-2-yl)indoles **3** and/or **4** (monitored by GC-MS and/or TLC). After the removal of the solvent, the purification and isolation of major isomers **3a** and **3c** was accomplished by silica gel column chromatography (hexane/Et_2O, 50:1).

1-Methyl-3-(3-methyl-1-phenyl-1*H*-inden-2-yl)-1*H*-indole (3a)

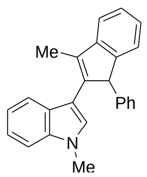

Following **GP2** from **1a** (167 mg, 0.5 mmol) as the starting 3-propargylindole a ca. 3.5:1 mixture of **3a**:**4a** was obtained. Indole derivative **3a** was isolated as a white solid (100 mg, 60%). M.p. 186–188 ºC. ^1H NMR (300 MHz, $CDCl_3$): δ 2.32 (s, 3H, CH_3C=), 3.68 (s, 3H, NCH_3), 4.96 (s, 1H, PhCH), 6.68 (s, 1H, =CH), 7.10–7.28 (m, 10H, ArH), 7.36 (td, *J* = 7.5, 1.3 Hz, 1H, ArH), 7.40–7.45 (m, 1H, ArH), 7.62–7.72 (m, 1H, ArH) ppm. ^{13}C NMR (75.4 MHz, $CDCl_3$): δ 12.7 (CH_3), 32.8 (CH_3), 58.9 (CH), 109.4 (CH), 111.3 (C), 118.7 (CH), 119.4 (CH), 120.9 (CH), 121.7 (CH), 123.6 (CH), 124.9 (CH), 126.5 (CH), 126.9 (CH), 127.5 (C), 128.0 (CH), 128.4 (2 × CH), 128.5 (2 × CH), 134.5 (C), 136.8 (C), 139.4 (C), 140.7 (C), 146.6 (C), 148.4 (C) ppm. LRMS (70 eV, EI): *m/z* (%) 335 (M^+, 100), 320 (77), 304 (16). HRMS calcd for $C_{25}H_{21}N$, 335.1674; found, 335.1684.

3-(6-Chloro-3-methyl-1-phenyl-1*H*-inden-2-yl)-1*H*-indole (3c)

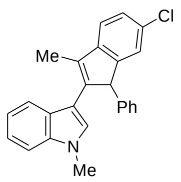

Following **GP2** from **1c** (185 mg, 0.5 mmol) as the starting 3-propargylindole a ca. 2.2:1 mixture of **3c**:**4c** was obtained. Indole derivative **3c** was isolated as a white solid (94 mg, 51%). M.p. 118–120 ºC. ^{1}H NMR (400 MHz, $CDCl_3$): δ 2.35 (s, 3H, CH_3C=), 3.63 (s, 3H, NCH_3),4.99 (s, 1H, PhCH), 6.73 (s, 1H, =CH), 7.13–7.25 (m, 7H, ArH), 7.28 (d, J = 3.6 Hz, 2H, ArH), 7.33 (d, J = 8.0 Hz, 1H, ArH), 7.37 (dd, J = 8.0, 1.8 Hz, 1H, ArH), 7.72 (d, J = 7.9 Hz, 1H, ArH) ppm. ^{13}C NMR (100.6 MHz, $CDCl_3$): δ 12.7 (CH_3), 32.8 (CH_3), 58.7 (CH), 109.5 (CH), 110.9 (C), 119.47 (CH), 119.53 (CH), 120.8 (CH), 121.8 (CH), 124.0 (CH), 126.8 (CH), 127.0 (CH), 127.3 (C), 128.1 (CH), 128.3 (2 × CH), 128.6 (2 × CH), 130.6 (C), 133.6 (C), 136.7 (C), 139.8 (C), 139.9 (C), 145.1 (C), 149.9 (C) ppm. LRMS (70 eV, EI): *m/z* (%) 371 [$(M+2)^+$, 33), 369 (M^+, 100), 354 (76), 334 (14). HRMS calcd for $C_{25}H_{20}ClN$, 369.1284; found, 369.1286.

General procedure 3 (GP3) for the (triarylphosphite)gold-catalyzed reaction of 3-propargylindoles 1. Synthesis of indole derivatives 3 and 4: To a stirred mixture of $[(2,4\text{-}(t\text{-Bu})_2C_6H_3O)_3P]AuCl$ (5 mol%) and AgOTf (5 mol%) in analytical grade toluene (2 mL/mmol), the corresponding 3-propargylindole **1** (1 equiv.) was added at 0 ºC. The resulting mixture was stirred at 0 ºC until complete conversion (monitored by GC-MS and/or TLC). After filtration through a short pad of celite (elution with hexane/AcOEt 5:1), the solvent was removed under reduced pressure and the residue purified by silica gel column chromatography (hexane/Et_2O or

hexane/AcOEt) to afford 3-(inden-2-yl)indoles **3** and/or **4**. Compounds **3b** and **3j-n** have been reported in our previous work [2,3].

1-Methyl-3-(1-methyl-1-phenyl-1*H*-inden-2-yl)-1*H*-indole (4a)

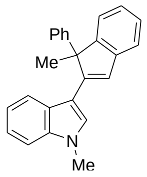

Following **GP3** from**1a** (150 mg, 0.45 mmol) as the starting 3-propargylindole a ca. 2.3:1 mixture of **4a**:**3a** was obtained. Indole derivative **4a** was isolated by column chromatography (hexane/ AcOEt, 50:1) as a white solid (90 mg, 60%). M.p. 138–140 ºC. ^{1}H NMR (400 MHz, $CDCl_3$): δ 1.80 (s, 3H, CH_3C), 3.61 (s, 3H, NCH_3), 6.43 (s, 1H, =CH), 7.05–7.08 (m, 2H, ArH), 7.19–7.35 (m, 9H, ArH), 7.37 (s, 1H, =CH), 7.43 (d, *J* = 7.4 Hz, 1H, ArH), 8.09–8.13 (m, 1H, ArH) ppm. ^{13}C NMR (100.6 MHz, $CDCl_3$): δ 23.7 (CH_3), 33.1 (CH_3), 58.3 (C), 109.6 (CH), 110.2 (C), 120.4 (CH), 120.6 (CH), 121.1 (CH), 122.3 (2 × CH), 123.1 (CH), 124.7 (CH), 126.3 (2 × CH), 126.5 (CH), 126.8 (CH), 127.2 (C), 128.3 (C), 128.7 (2 × CH), 137.2 (C), 143.2 (C), 143.8 (C), 150.9 (C), 154.6 (C) ppm. IR (KBr): ν 2964, 1465, 1455, 1372, 1332, 699 cm^{-1}. LRMS (70 eV, EI): *m/z* (%) 335 (M^+, 100), 320 (79), 304 (12). HRMS calcd for $C_{25}H_{21}N$, 335.1674; found, 335.1680.

3-(1-Methyl-1-phenyl-1*H*-inden-2-yl)-1*H*-indole (4b)

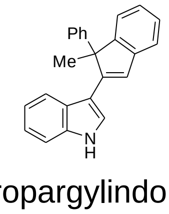

Following **GP3** from **1b** (95 mg, 0.29 mmol) as the starting 3-propargylindole a ca. 1.8:1 mixture of **4b**:**3b** was obtained. Indole derivative **4b** was isolated by column chromatography (hexane/Et_2O, 20:1) as a white-grey solid (36 mg, 38%). M.p. 183–185 ºC. ^{1}H NMR (300 MHz, $CDCl_3$): δ 1.81 (s, 3H, CH_3C), 6.57 (s, 1H, =CH), 7.09 (d, *J* = 4.2 Hz, 1H, ArH), 7.19–7.51 (m, 12H, ArH), 7.93 (s, 1H, NH), 8.09–8.18

(m, 1H, ArH) ppm. ^{13}C NMR (75.4 MHz, $CDCl_3$): δ 23.7 (CH_3), 58.6 (C), 111.6 (CH), 111.9 (CH), 120.9 (2 × CH), 121.1 (CH), 122.5 (CH), 122.9 (CH), 123.8 (C), 123.9 (CH), 125.0 (CH), 126.5 (2 × CH), 126.7 (CH), 127.2 (CH), 128.7 (C), 128.8 (2 × CH), 136.4 (C), 143.3 (C), 144.0 (C), 151.0 (C), 154.7 (C) ppm. LRMS (70 eV, EI): *m/z* (%) 321 (M^+, 100), 306 (55), 304 (22), 244 (9). HRMS calcd for $C_{24}H_{19}N$, 321.1517; found, 321.1519.

3-[1-(4-Chlorophenyl)-1-methyl-1*H*-inden-2-yl]-1-methyl-1*H*-indole (4c)

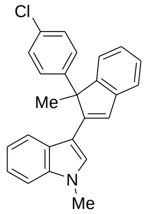

Following **GP3** from **1c** (169 mg, 0.45 mmol) as the starting 3-propargylindole a ca. 3:1 mixture of **4c**:**3c** was obtained. Indole derivative **4c** was isolated by column chromatography (hexane/AcOEt, 50:1) as a yellow foam (113 mg, 67%). R_f 0.26 (hexane/ AcOEt, 5:1). ^{1}H NMR (400 MHz, $CDCl_3$): δ 1.80 (s, 3H, CH_3C), 3.65 (s, 3H, NCH_3), 6.46 (s, 1H, =CH), 7.05 (d, *J* = 7.1 Hz, 1H, ArH), 7.09 (dt, *J* = 7.3, 0.8 Hz, 1H, ArH), 7.21–7.30 (m, 6H, ArH), 7.32–7.35 (m, 2H, ArH), 7.39 (s, 1H, =CH), 7.45 (d, *J* = 7.4 Hz, 1H, ArH), 8.12 (d, *J* = 6.7 Hz, 1H, ArH) ppm. ^{13}C NMR (100.6 MHz, $CDCl_3$): δ 23.7 (CH_3), 33.1 (CH_3), 57.9 (C), 109.7 (CH), 110.0 (C), 120.5 (CH), 120.7 (CH), 121.1 (CH), 122.2 (CH), 122.5 (CH), 123.3 (CH), 124.8 (CH), 127.1 (CH), 127.8 (2 × CH), 128.1 (CH), 128.9 (2 × CH), 132.3 (C), 137.2 (C), 142.7 (C), 143.1 (C), 150.4 (C), 154.0 (C) ppm. LRMS (70 eV, EI): *m/z* (%) 371 [$(M+2)^+$, 15), 369 (M^+, 45), 354 (100), 334 (32). HRMS calcd for $C_{25}H_{20}ClN$, 369.1284; found, 369.1281.

3-[1-(2-Bromophenyl)-1-methyl-1*H*-inden-2-yl]-1-methyl-1*H*-indole (4d)

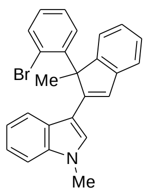

Following **GP3** from**1d** (100 mg, 0.24 mmol) as the starting 3-propargylindole a ca. >10:1 mixture of **4d**:**3d** was obtained. Indole derivative **4d** was isolated by column chromatography (hexane/AcOEt, 20:1) as a white solid (86 mg, 86%). M.p. 164–166 ºC. ^{1}H NMR (400 MHz, $CDCl_3$): δ 1.87 (s, 3H, CH_3C), 3.58 (s, 3H, NCH_3), 6.36 (s, 1H, =CH), 7.03 (d, *J* = 7.3 Hz, 1H, ArH), 7.18–7.25 (m, 2H, ArH), 7.30–7.40 (m, 4H, ArH), 7.44 (s, 1H, =CH), 7.52–7.56 (m, 3H, ArH), 8.02 (d, *J* = 7.7 Hz, 1H, ArH), 8.20–8.22 (m, 1H, ArH) ppm. ^{13}C NMR (100.6 MHz, $CDCl_3$): δ 29.2 (CH_3), 33.0 (CH_3), 59.7 (C), 109.5 (CH), 110.8 (C), 120.3 (CH), 120.9 (CH), 121.2 (CH), 121.4 (CH), 122.3 (CH), 124.1 (CH), 124.5 (CH), 124.7 (C), 126.8 (CH), 127.2 (CH + C), 127.3 (CH), 128.6 (CH), 129.9 (CH), 135.3 (CH), 137.2 (C), 142.5 (C), 145.1 (C), 149.3 (C), 151.7 (C) ppm. LRMS (70 eV, EI): *m/z* (%) 415 [$(M+2)^+$, 98), 413 (M^+, 100), 334 (36), 319 (48). HRMS calcd for $C_{25}H_{20}BrN$, 413.0779; found,413.0770.

1-Methyl-3-(6-methyl-6-phenyl-6*H*-cyclopenta[*b*]thiophen-5-yl)-1*H*-indole (4e)

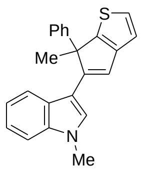

Following **GP3** from **1e** (300 mg, 0.87 mmol) as the starting 3-propargylindole a ca. 3:1 mixture of **4e**:**3e** was obtained. Indole derivative **4e** was isolated by column chromatography (hexane/Et_2O, 80:1) as a white solid (185 mg, 62%). M.p. 130–132 ºC. ^{1}H NMR (400 MHz, $CDCl_3$): δ = 1.83 (s, 3H, CH_3C), 3.60 (s, 3H, NCH_3), 6.37 (s, 1H, =CH), 7.03 (d, *J* = 4.9, 1H, ArH), 7.18–7.37 (m, 10H, ArH), 8.00 (d, *J* = 7.8, 1H, ArH) ppm. ^{13}C NMR (75.4 MHz, $CDCl_3$): δ 24.8 (CH_3), 32.9 (CH_3), 56.9 (CH), 109.5 (CH), 110.8 (C), 119.3 (CH), 119.5 (CH), 120.1 (CH), 120.9 (CH), 122.2 (CH), 126.3

(2 × CH), 126.8 (CH), 126.8 (C), 127.0 (CH), 127.7 (CH), 128.8 (2 × CH), 137.0 (C), 143.5 (C), 145.9 (C), 153.6 (C), 154.9 (C) ppm. LRMS (70 eV, EI): *m/z* (%) 341 (M^+, 100), 327 (15), 326 (55), 310 (8), 364 (10). HRMS calcd for $C_{23}H_{19}NS$, 341.1238; found, 341.1241.

3-[6-(4-Chlorophenyl)-6-methyl-6*H*-cyclopenta[*b*]thiophen-5-yl]-1-methyl-1*H*-indole (4f)

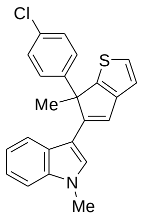

Following **GP3** from **1f** (292 mg, 0.75 mmol) as the starting 3-propargylindole a ca. 4:1 mixture of **4f**:**3f** was obtained. Indole derivative **4f** was isolated by column chromatography (hexane/Et_2O, 50:1) as a white solid (207 mg, 71%) M.p. 155 ºC with decomposition. ^1H NMR (400 MHz, $CDCl_3$): δ 1.90 (s, 3H, CH_3C), 3.66 (s, 3H NCH_3), 6.47 (s, 1H =CH, 7.10 (d, *J* = 4.8 Hz, 1H, ArH), 7.29–7.38 (m, 9H, ArH), 8.07–8.11 (m, 1H, ArH) ppm. ^{13}C NMR (75.4 MHz, $CDCl_3$): δ 24.7 (CH_3), 33.0 (CH_3), 56.3 (C), 109.6 (CH), 110.6 (C), 119.4 (CH), 119.7 (CH), 120.2 (CH), 120.9 (CH), 122.4 (CH), 126.7 (C), 126.8 (CH), 127.7 (2 × CH), 128.0 (CH), 128.9 (2 × CH), 132.5 (C), 137.0 (C), 142.3 (C), 146.0 (C), 153.2 (C), 154.3 (C) ppm. LRMS (70 eV, EI): *m/z* (%) 377 [$(M+2)^+$, 39], 375 (M^+, 100), 362 (33), 360 (82), 324 (15), 264 (17). HRMS calcd for $C_{23}H_{18}ClNS$, 375.0848; found, 375.0846.

3-(1-(4-Chlorophenyl)-6-methoxy-1-methyl-1*H*-inden-2-yl)-1-methyl-1*H*-indole (4g)

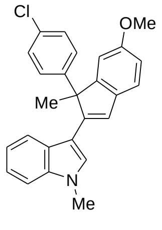

Following **GP3** from**1g** (399.7 mg, 1 mmol) as the starting 3-propargylindole a ca. 3:1 mixture of **4g**:**3g** was obtained. Indole derivative **4g** was isolated by column chromatography (hexane/Et_2O, 60:1) as a white solid (239 mg, 60%). M.p. 172–174

ºC. ^{1}H NMR (400 MHz, $CDCl_3$): δ 1.78 (s, 3H, CH_3C), 3.63 (s, 3H, NCH_3), 3.76 (s, 3H, OCH_3), 6.40 (s, 1H, =CH), 6.63 (d, J = 2.3 Hz, 1H, ArH), 6.80 (dd, *J* = 8.2, 2.3 Hz, 1H, ArH), 7.20–7.35 (m, 9H, ArH), 8.08 (d, *J* = 7.8 Hz, 1H, ArH). ppm. ^{13}C NMR (75.4 MHz, $CDCl_3$): δ 23.8 (CH_3), 33.0 (CH_3), 55.2 (CH_3), 57.9 (C), 109.3 (C), 109.6 (CH), 110.1 (C), 111.8 (CH), 120.3 (CH), 121.0 (CH), 121.0 (CH), 122.3 (CH), 122.9 (CH), 126.9 (C), 127.5 (CH), 127.2 (2 × CH), 128.8 (2 × CH), 132.2 (C), 136.0 (C), 137.1 (C), 142.7 (C), 148.4 (C), 155.9 (C), 157.9 (C) ppm. LRMS (70 eV, EI): *m/z* (%) 401 [$(M+2)^+$, 36], 399 (M^+, 100), 386 (17), 384 (42), 341 (7). HRMS calcd for $C_{26}H_{22}ClNO$, 399.1390; found, 399.1397.

3-(6-Chloro-1-methyl-1-phenyl-1*H*-inden-2-yl)-1-methyl-1*H*-indole (4h) / 3-(1-(4-chlorophenyl)-3-methyl-1H-inden-2-yl)-1-methyl-1*H*-indole (3h)

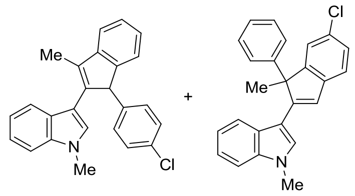

Following **GP3** from **1h** (110 mg, 0.3 mmol) as the starting 3-propargylindole a ca. 1.2:1 mixture of **4h**:**3h** was obtained. The components could not be completely separated by column chromatography (hexane/Et_2O, 50:1). The analytical data reported were obtained from a 1:1 mixture of **4h** and **3h**. ^{1}H NMR (300 MHz, $CDCl_3$): δ 1.83 (s, 3H, CH_3C, **4h**), 2.33 (s, 3H, CH_3C=, **3h**), 3.61 (s, 3H, NCH_3, **4h**), 3.73 (s, 3H, NCH_3, **3h**), 4.98 (s, 1H, PhCH, **3h**), 6.45 (s, 1H, =CH, **4h**), 6.77 (s, 1H, =CH, **3h**), 7.03–7.07 (m, 3H, ArH), 7.13–7.45 (m, 20H, ArH), 7.67–7.70 (m, 1H, ArH), 8.10–8.13 (m, 1H, ArH) ppm. ^{13}C NMR (75.4 MHz, $CDCl_3$): δ 12.7 (CH_3, **3h**), 23.6 (CH_3, **4h**), 32.95 (CH_3), 33.03 (CH_3), 58.0 (CH, **3h**), 57.9 (C, **4h**), 109.5 (CH, **3h**), 109.7 (CH, **4h**), 109.9 (C, **4h**), 111.0 (C, **3h**), 118.9 (CH), 119.6 (CH), 120.5 (CH), 120.7 (CH), 121.0 (CH), 121,4 (CH), 121.8 (CH), 122.1 (CH), 122.4 (CH), 122.9 (CH), 123.5 (CH), 125.0 (CH), 126.3 (2 × CH), 126.9 (CH), 127.0 (CH), 127.1 (CH), 127.5 (C),

128.0 (CH), 128.4 (CH), 128.7 (2 × CH), 128.9 (2 × CH), 129.7 (2 × CH), 130.2 (C), 131.1 (C), 132.1 (C), 134.7 (C), 136.8 (C), 137.2 (C), 139.1 (C), 139.4 (C) 141.7 (C), 143.0 (C), 146.5 (C), 147.9 (C), 151.5 (C), 156.2 (C) ppm.

3-[1-(4-Chlorophenyl)-6-methoxy-3-methyl-1*H*-inden-2-yl]-1-methyl-1*H*-indole (3i)

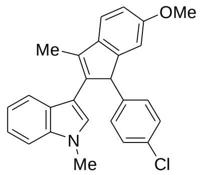

Following **GP3** from **1i** (200 mg, 0.5 mmol) as the starting 3-propargylindole a ca. >10:1 mixture of **3i**:**4i** was obtained. Indole derivatives **3i** and **3´i** were isolated by column chromatography on neutral alumina (hexane/Et_2O, 20:1) in 88% overall yield. Data for **3i**: Yellow solid. M.p. 69 ºC with decomposition. ^{1}H NMR (400 MHz, $CDCl_3$): δ 2.26 (s, 3H, CH_3C), 3.70 (s, 3H, XCH_3), 3.78 (s, 3H, XCH_3), 4.90 (s, 1H, ArCH), 6.70 (s, 1H, ArH), 6.78 (d, *J* = 2.4 Hz, 1H, ArH), 6.90 (dd, *J* = 2.4, 8.1 Hz, 1H, ArH), 7.01 (d, *J* = 8.5 Hz, 2H, ArH), 7.12 (d, *J* = 8.5 Hz, 2H, ArH), 7.10–7.16 (m, 1H, ArH), 7.21–7.30 (m, 3H, ArH), 7.63 (d, *J* = 8.1 Hz, 1H, ArH) ppm. ^{13}C NMR (75.4 MHz, $CDCl_3$): δ 12.7 (CH_3), 32.9 (CH_3), 55.6 (CH_3), 58.0 (CH), 109.4 (CH), 110.4 (CH), 111.1 (C), 112.2 (CH), 119.2 (CH), 119.4 (CH), 120.7 (CH), 121.7 (CH), 127.5 (C), 127.8 (CH), 128.7 (2 × CH), 129.7 (2 × CH), 132.1 (C), 134.4 (C), 136.8 (C), 137.0 (C), 139.5 (C), 139.6 (C), 149.6 (C), 158.2 (C) ppm. LRMS (70 eV, EI): *m/z* (%) 401 [$(M+2)^+$, 32], 399 (M^+, 100), 384 (29). HRMS calcd for $C_{26}H_{22}ClNO$, 399.1390; found, 399.1397.

3-[3-(4-Chlorophenyl)-5-methoxy-1-methyl-1*H*-inden-2-yl]-1-methyl-1*H*-indole (3´i)

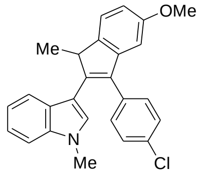

Data for **3´i**: Yellow solid. M.p. 197–199 ºC. ^{1}H NMR (400 MHz, $CDCl_3$): δ 1.3 (d, *J* = 7.5 Hz, 3H, CH_3C), 3.72 (s, 3H, XCH_3), 3.81 (s, 3H, XCH_3), 4.15 (q, *J* = 7.5 Hz, 1H,

$CHCH_3$), 6.77–6.84 (m, 3H, ArH), 7.03 (ddd, *J* = 1.0, 7.0, 8.0 Hz, 1H, ArH), 7.21 (ddd, *J* = 1.0, 7.0, 8.0 Hz, 1H, ArH), 7.24–7.45 (m, 7H, ArH) ppm. ^{13}C NMR (75.4 MHz, $CDCl_3$): δ 17.5 (CH_3), 33.1 (CH_3), 46.0 (CH), 55.7 (CH_3), 105.3 (CH), 109.4 (CH), 110.1 (CH), 110.5 (C), 119.9 (CH), 120.9 (CH), 121.9 (CH), 123.3 (CH), 126.8 (C), 128.9 (2 × CH), 129.4 (CH), 131.0 (2 × CH), 132.7 (C), 143.3 (C), 135.2 (C), 137.1 (C), 140.8 (C), 144.9 (C), 146.4 (C), 159.2 (C) ppm. LRMS (70 eV, EI): *m/z* (%) 401 [$(M+2)^+$, 33) 399 (M^+, 100), 386 (16), 384 (47), 341 (8), 152 (8). HRMS calcd for $C_{26}H_{22}ClNO$, 399.1390; found, 399.1385.

1-methyl-3-(1-methyl-1-phenyl-1*H*-inden-2-yl)-2-phenyl-1*H*-indole (4k)

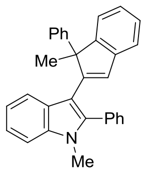

Following **GP3** from **1k** (165 mg, 0.4 mmol) as the starting 3-propargylindole a ca 1.6:1 of **3k**:**4k** was obtained. Indole derivative **4k** was isolated by column chromatography (hexane/Et_2O, 20:1) as a yellow foam (56 mg, 34%). ^{1}H NMR (300 MHz, $CDCl_3$): δ 1.54 (s, 3H, CH_3C), 3.54 (s, 3H, NCH_3), 6.70–6.75 (m, 2H, ArH), 6.81–7.07 (m, 7H, ArH), 7.10–7.36 (m, 10H, ArH) ppm. ^{13}C NMR (75.4 MHz, $CDCl_3$): δ 22.3 (CH_3), 31.1 (CH_3), 60.2 (C), 109.1 (CH), 109.7 (C), 119.6 (CH), 120.8 (CH), 120.9 (CH), 121.6 (CH), 123.1 (CH), 125.3 (CH), 126.2 (CH), 126.7 (CH), 127.4 (C), 127.0 (2 × CH), 127.9 (CH), 127.96 (2 × CH), 128.04 (2 × CH), 130.8 (2 × CH),131.7 (CH), 132.3 (C), 136.8 (C), 139.5 (C), 142.4 (C), 143.3 (C), 152.2 (C), 155.1 (C) ppm. LRMS (70 eV, EI): *m/z* (%) 411 (M^+, 100), 396 (32), 318 (19).

3-(1-Ethyl-1-phenyl-1*H*-inden-2-yl)-1-methyl-1*H*-indole (4l)

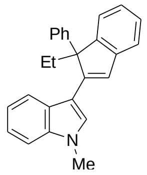

Following **GP3** from **1l** (174.7 mg, 0.5 mmol) as the starting 3-propargylindole a ca. 3:1 mixture of **3l**:**4l** was obtained. Indole derivative **4l** was isolated by column chromatography (hexane/Et_2O, 50 :1) as a white solid (34.9 mg, 20%). M.p. 156–158 ºC. ^1H NMR (300 MHz, $CDCl_3$): δ 0.38 (t, *J* = 7.2 Hz, 3H, CH_3CH_2), 2.28–2.43 (m, 1H, CH_3CHH), 2.56–2.72 (m, 1H, CH_3CHH), 3.61 (s, 3H, NCH_3), 6.44 (s, 1H, =CH), 6.97–7.07 (m, 2H, ArH), 7.17–7.39 (m, 10H, ArH), 7.42 (s, 1H, =CH), 8.12 (dd, *J* = 5.7, 2.4 Hz, 1H, ArH) ppm. ^{13}C NMR (75.4 MHz, $CDCl_3$): δ 7.9 (CH_3), 28.5 (CH_2), 33.1 (CH_3), 62.7 (C), 109.6 (CH), 110.4 (C), 120.2 (CH), 120.3 (CH), 121.0 (CH), 122.2 (CH), 122.3 (CH), 124.6 (CH), 125. 3 (CH), 126.49 (2 × CH), 126.52 (CH), 126.8 (CH), 127.0 (C), 128.0 (CH), 128.7 (2 × CH), 137.1 (C), 144.4 (C), 144.7 (C), 148.0 (C), 152.4 (C) ppm. LRMS (70 eV, EI): *m/z* (%) 349 (M^+, 74), 320 (100), 304 (18). HRMS calcd for $C_{26}H_{23}N$, 349.1830; found, 349.1827.

1-Methyl-3-(1-phenyl-1-propyl-1*H*-inden-2-yl)-1*H*-indole (4m)

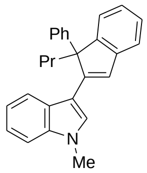

Following **GP3** from **1m** (140 mg, 0.38 mmol) as the starting 3-propargylindole a ca. 3:1 mixture of **3m**:**4m** was obtained. Indole derivative **4m** was isolated, slightly contaminated with an unknown impurity, by column chromatography (hexane/Et_2O, 70:1) as a pale yellow solid (28 mg, 19%). M.p. 158–160 ºC. ^1H NMR (300 MHz, $CDCl_3$): δ 0.48–0.60 (m, 1H, CH_3CHH) 0.66 (t, *J* = 7.2Hz, 3H, CH_3CH_2), 0.86–0.96 (m, 1H, CH_3CHH), 2.30 (td, *J* = 12.2, 4.2 Hz, 1H, CH_3CH_2 CHH), 2.48 (td, *J* = 12.3, 4.2 Hz, 1H, CH_3CH_2 CHH), 3.60 (s, 3H), 6.42 (s, 1H), 7.01–7.04 (m, 2H), 7.15–7.40

(m, 11H), 8.07–8.10 (m, 1H) ppm. ^{13}C NMR (75.4 MHz, $CDCl_3$): δ 14.8 (CH_3), 16.5 (CH_2), 17.5 (CH_2), 33.2 (CH_3), 62.3 (C), 109.7 (CH), 110.5 (C), 120.3 (CH), 120.4 (CH), 121.1 (CH), 122.2 (CH), 122.4 (CH), 124.7 (CH), 124.9 (CH), 126.5 (2 × CH), 126.6 (CH), 126.8 (CH), 127.1 (C), 128.2 (C), 128.8 (2 × CH), 137.2 (C), 144.5 (C), 144.5 (C), 148.6 (C), 153.0 (C) ppm. LRMS (70 eV, EI): *m/z* (%) 363 (M^+, 100), 321 (47), 320 (100), 304 (22), 160. HRMS calcd for $C_{27}H_{25}N$, 363.1987; found, 363.1988.

References

1. Sanz, R.; Miguel, D.; Martínez, A.; Gohain, M.; García-García, P.; Fernández-Rodríguez, M. A.; Álvarez, E.; Rodríguez, F. *Eur. J. Org. Chem.* **2010**, 7027–7039. doi:10.1002/ejoc.201001055

2. Sanz, R.; Miguel, D.; Rodríguez, F. *Angew. Chem., Int. Ed.* **2008**, *47*, 7354–7357. doi:10.1002/anie.200802660

3. Sanz, R.; Miguel, D.; Gohain, M.; García-García, P.; Fernández-Rodríguez, M. A.; González-Pérez, A.; Nieto-Faza, O.; de Lera, A. R.; Rodríguez, F. *Chem. Eur. J.* **2010**, *16*, 9818–9828. doi:10.1002/chem.201001162
