# Supplementary material for: Solvent- and ligand-induced switch of selectivity in gold(I)-catalyzed tandem reactions of 3-propargylindoles
Source: Beilstein J Org Chem. 2011 Jun 9;7:786–93. doi: 10.3762/bjoc.7.89 (PMC3135218; doi:10.3762/bjoc.7.89)
Supplement: File 2 — NMR spectra. [file Beilstein_J_Org_Chem-07-786-s002.pdf]

Supporting Information

for

Solvent- and ligand-induced switch of selectivity in gold(I)-catalyzed tandem reactions of 3-propargylindoles

Estela Álvarez[1], Delia Miguel[1], Patricia García-García[1], Manuel A. Fernández-Rodríguez[1], Félix Rodríguez[2] and Roberto Sanz*[1]

Address: [1]Área de Química Orgánica, Departamento de Química, Facultad de Ciencias, Universidad de Burgos, Pza. Misael Bañuelos s/n, 09001 Burgos, Spain and [2]Instituto Universitario de Química Organometálica “Enrique Moles”, Universidad de Oviedo, C/Julián Clavería 8, 33006 Oviedo, Spain

Email: Roberto Sanz - rsd@ubu.es

*Corresponding author

NMR spectra

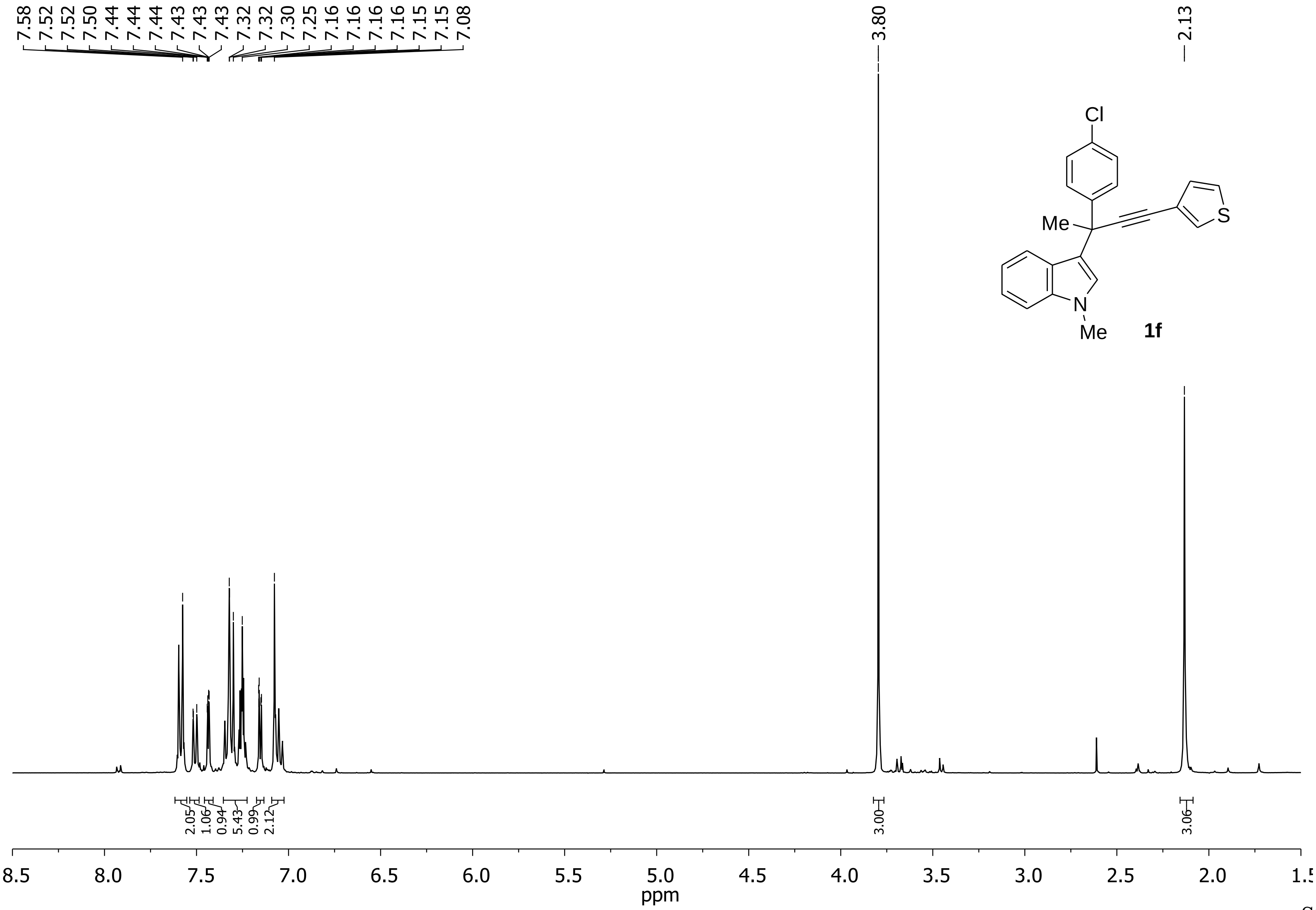
7.58
7.52
7.52
7.50
7.44
7.44
7.44
7.43
7.43
7.43
7.32
7.32
7.30
7.25
7.16
7.16
7.16
7.16
7.15
7.15
7.08
3.80
2.13
Cl
Me
S
N
Me
1f
2.05
1.06
0.94
5.43
0.99
2.12
3.00
3.06
8.5
8.0
7.5
7.0
6.5
6.0
5.5
5.0
4.5
4.0
3.5
3.0
2.5
2.0
ppm

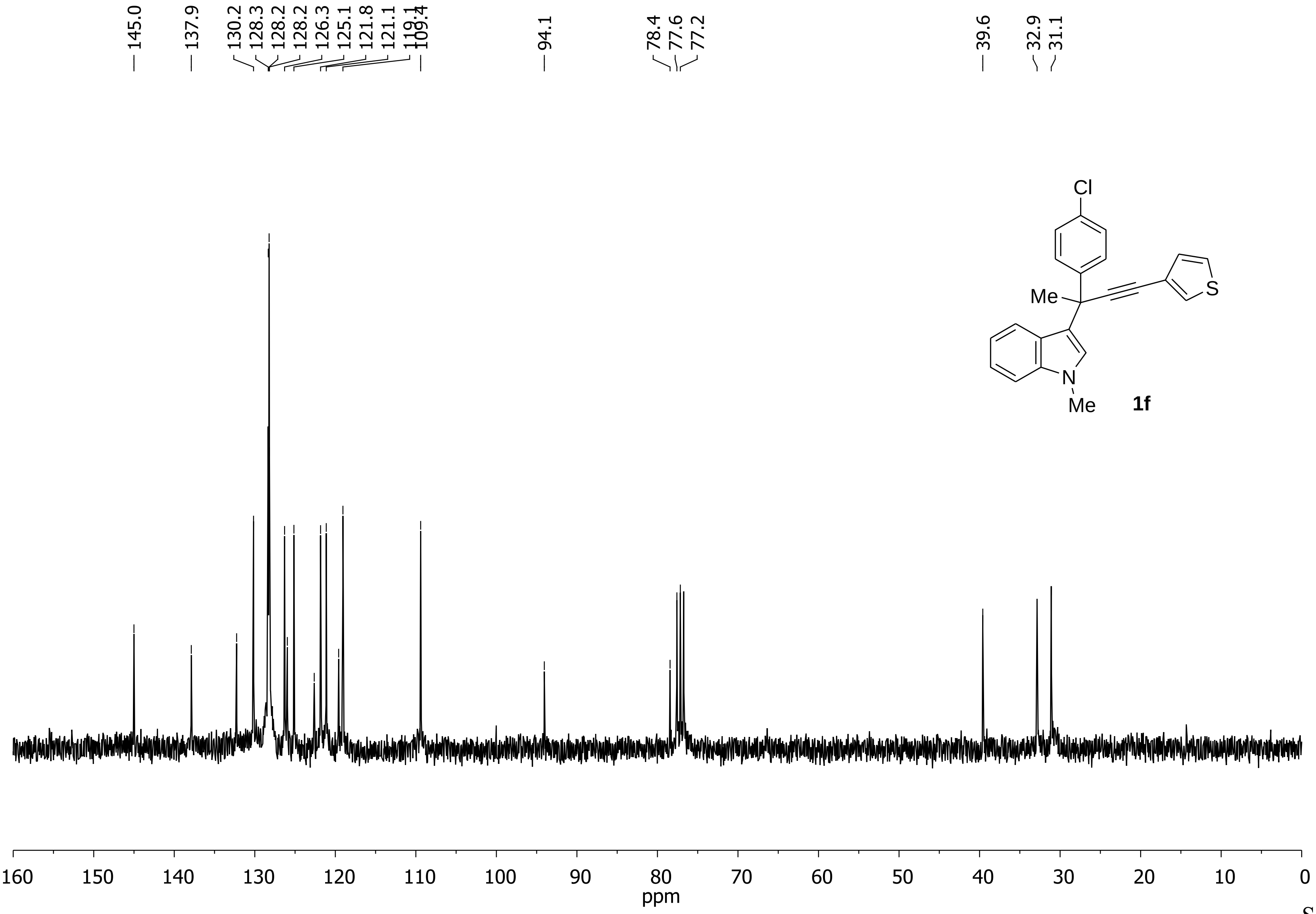
145.0
137.9
130.2
128.3
128.2
128.2
126.3
125.1
121.8
121.1
119.1
109.4
94.1
78.4
77.6
77.2
39.6
32.9
31.1
Cl
Me
S
N
Me
1f
160
150
140
130
120
110
100
90
80
70
60
50
40
30
20
10
0
ppm

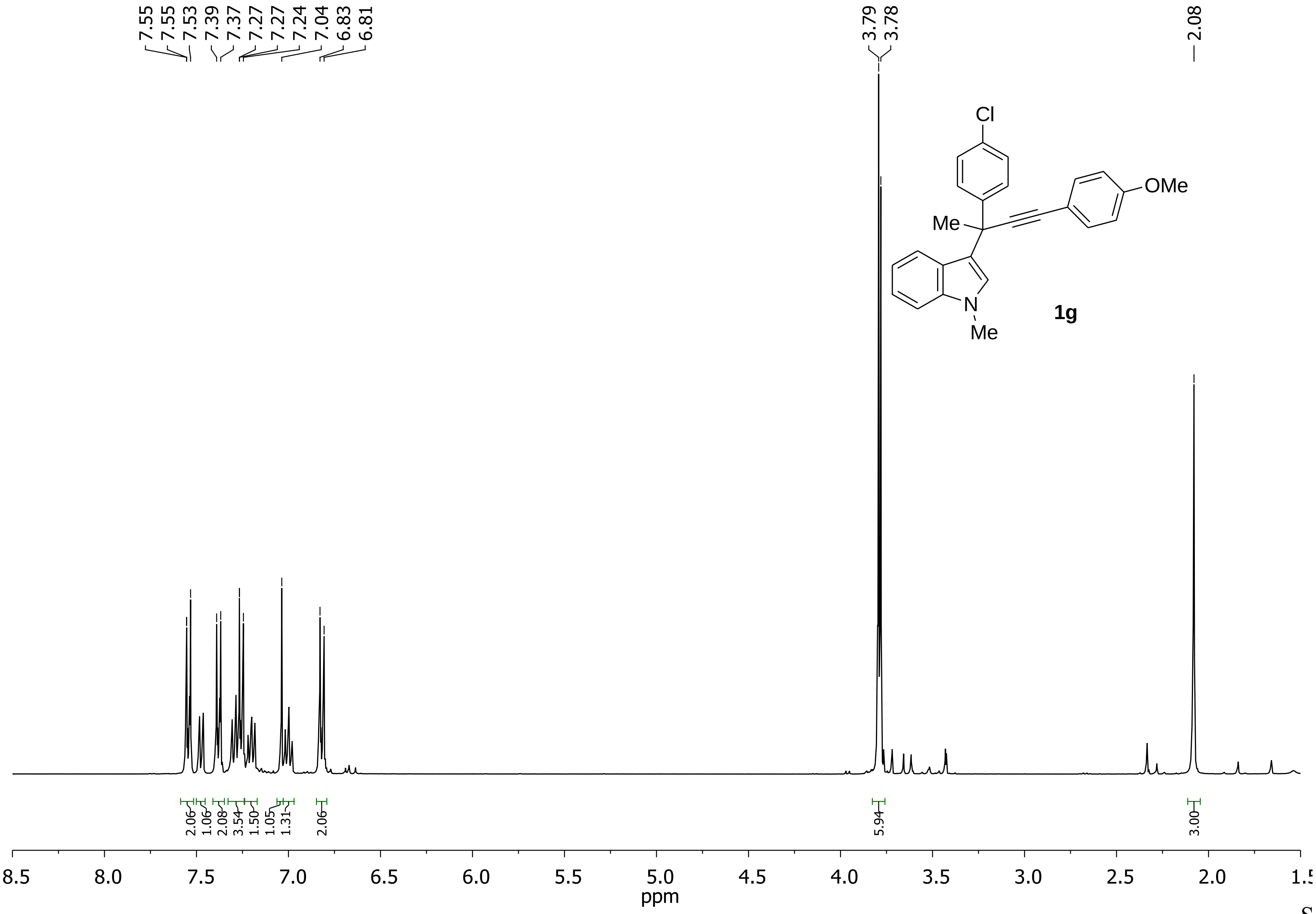
7.55
7.55
7.53
7.39
7.37
7.27
7.27
7.24
7.04
6.83
6.81
3.79
3.78
2.08
Cl
OMe
Me
N
Me
1g
2.06
1.06
2.08
3.54
1.50
1.05
1.31
2.06
5.94
3.00
8.5
8.0
7.5
7.0
6.5
6.0
5.5
5.0
4.5
4.0
3.5
3.0
2.5
2.0
ppm

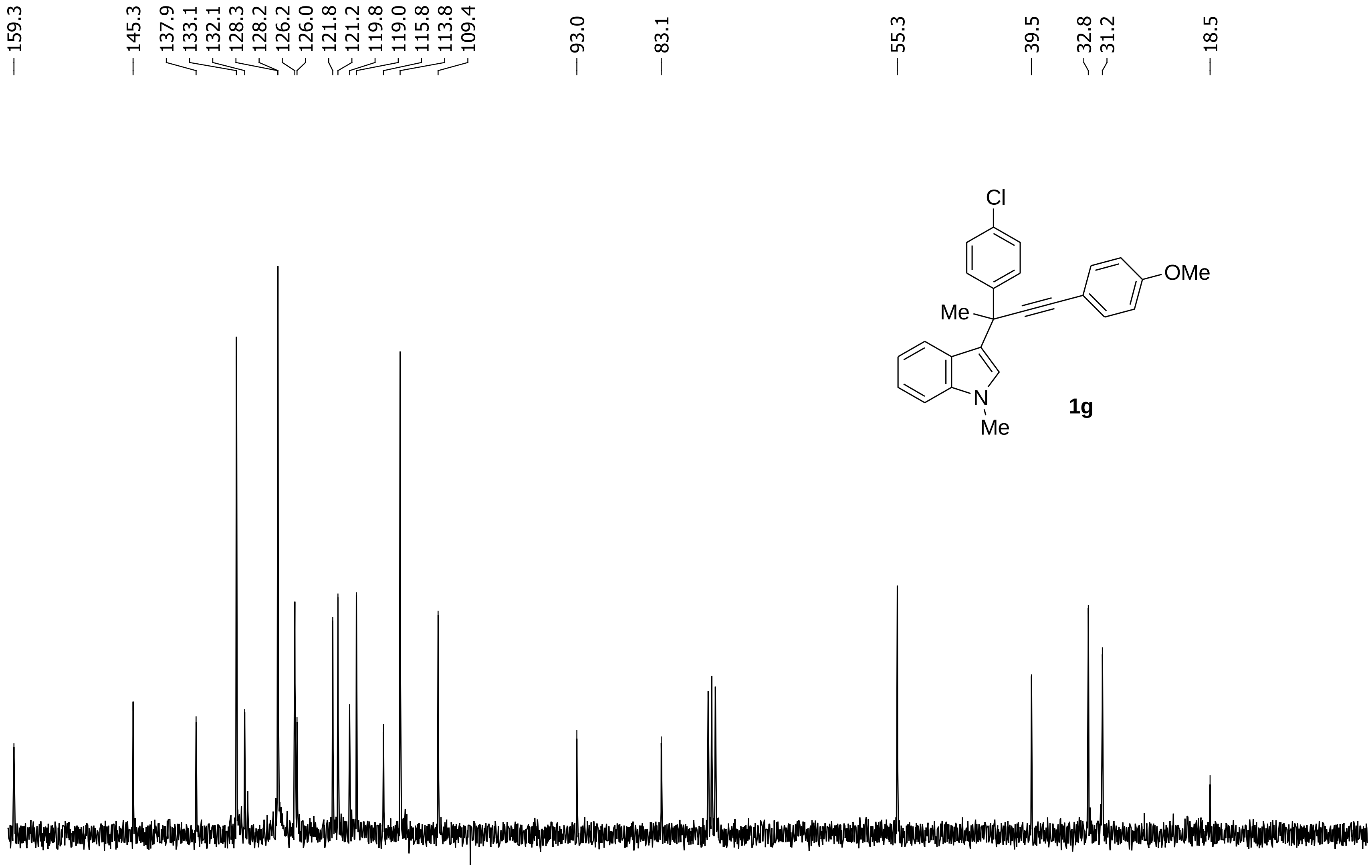
159.3
145.3
137.9
133.1
132.1
128.3
128.2
126.2
126.0
121.8
121.2
119.8
119.0
115.8
113.8
109.4
93.0
83.1
55.3
39.5
32.8
31.2
18.5
Cl
OMe
Me
N
Me
1g

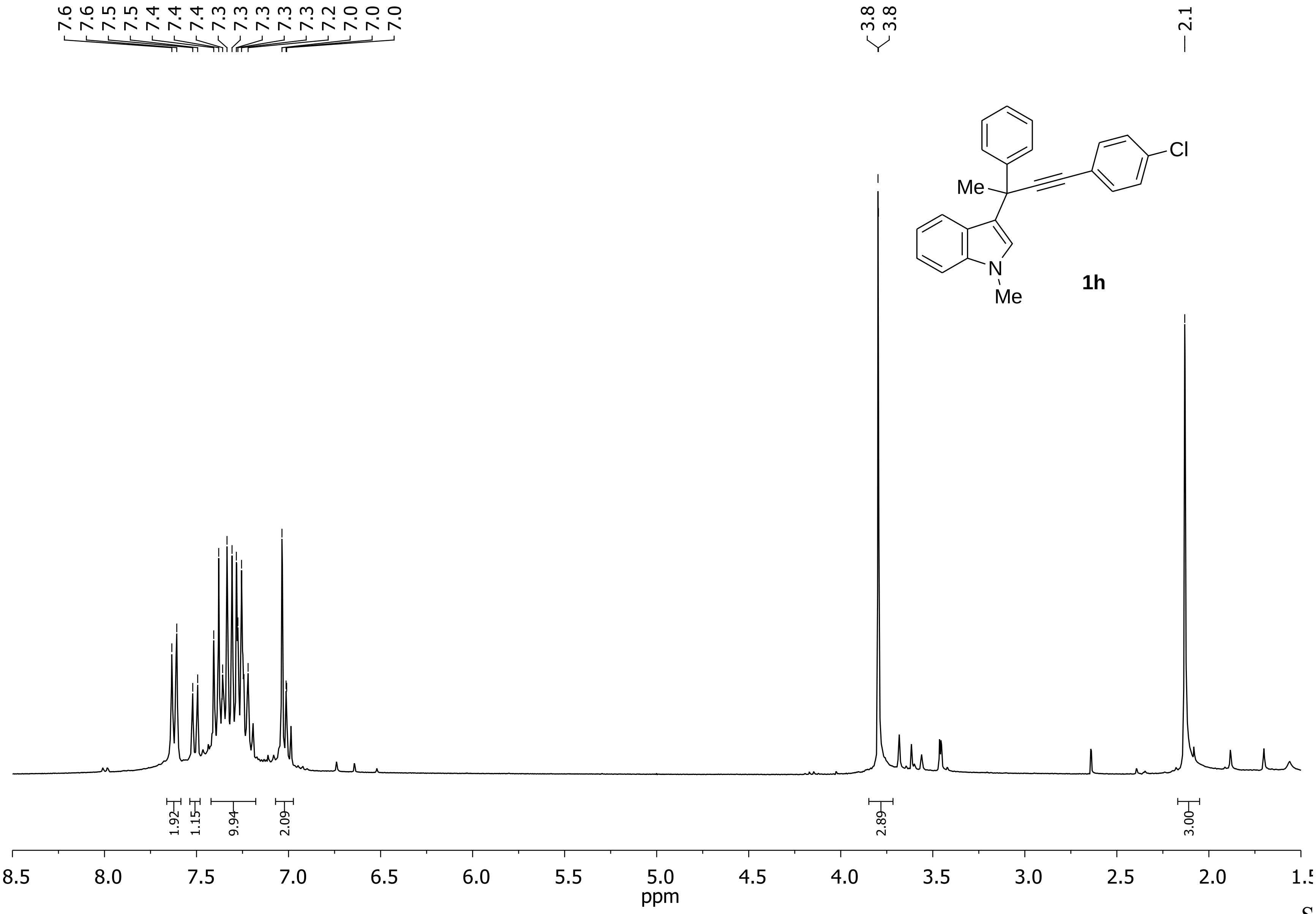
7.6
7.6
7.5
7.5
7.4
7.4
7.4
7.3
7.3
7.3
7.3
7.3
7.2
7.0
7.0
7.0
3.8
3.8
2.1
Me
Cl
N
Me
1h
1.92
1.15
9.94
2.09
2.89
3.00
8.5
8.0
7.5
7.0
6.5
6.0
5.5
5.0
4.5
4.0
3.5
3.0
2.5
2.0
ppm

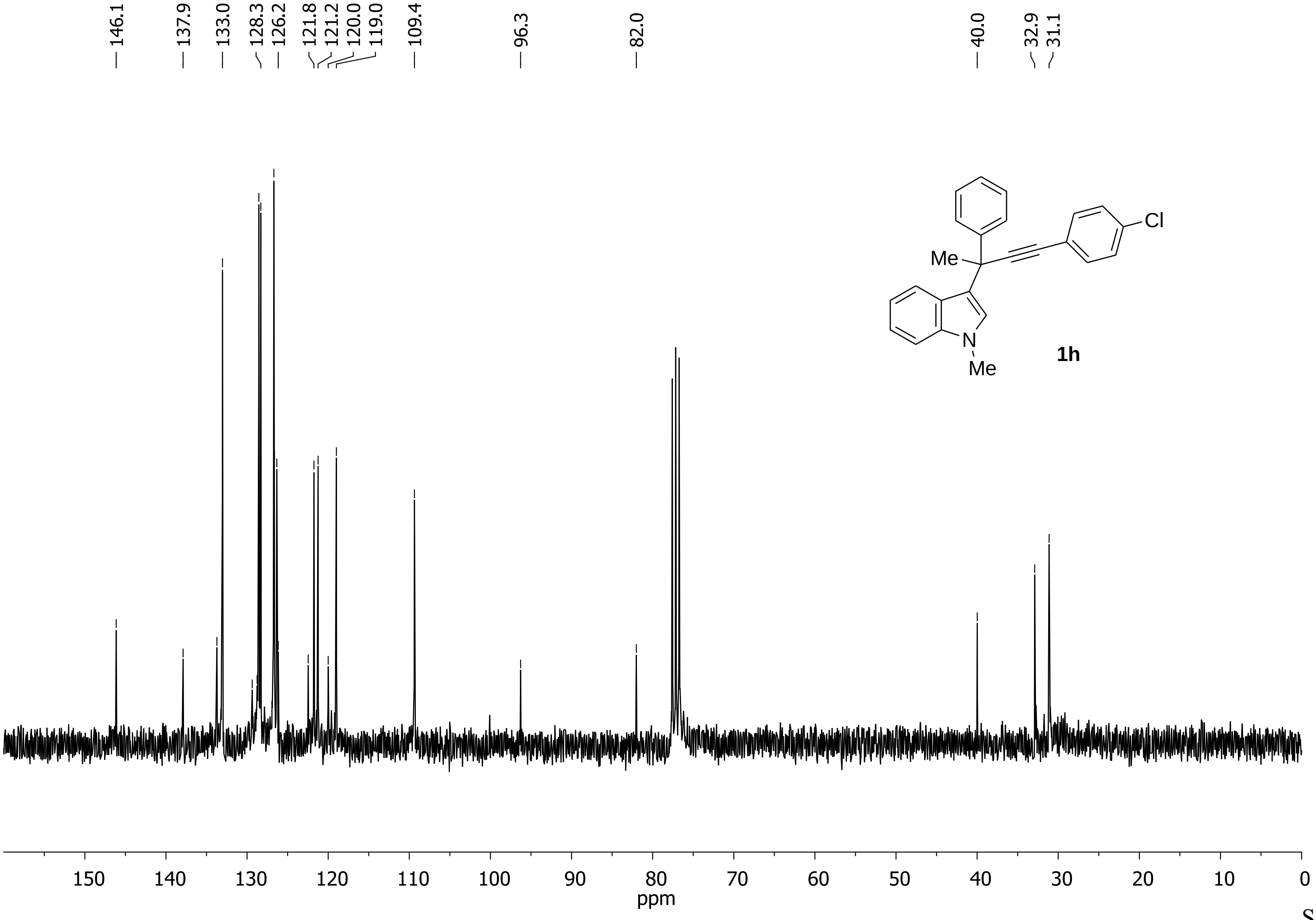

146.1
137.9
133.0
128.3
126.2
121.8
121.2
120.0
119.0
109.4
96.3
82.0
40.0
32.9
31.1
Me
Cl
N
Me
1h
150
140
130
120
110
100
90
80
70
60
50
40
30
20
10
0
ppm

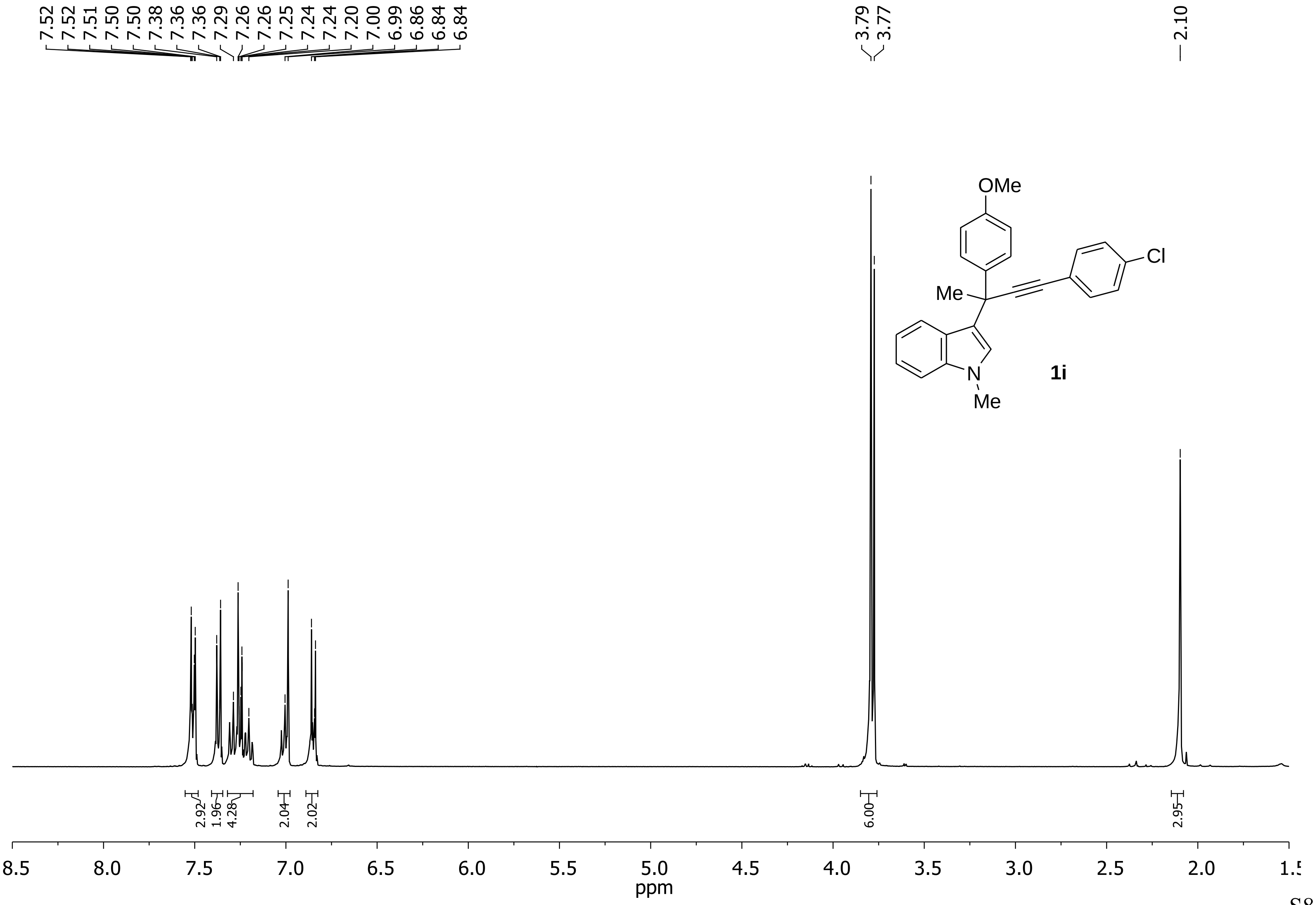
7.52
7.52
7.51
7.50
7.50
7.38
7.36
7.36
7.29
7.26
7.26
7.25
7.24
7.24
7.20
7.00
6.99
6.86
6.84
6.84
3.79
3.77
2.10
OMe
Cl
Me
N
Me
1i
2.92
1.96
4.28
2.04
2.02
6.00
2.95
8.5
8.0
7.5
7.0
6.5
6.0
5.5
5.0
4.5
4.0
3.5
3.0
2.5
2.0
ppm

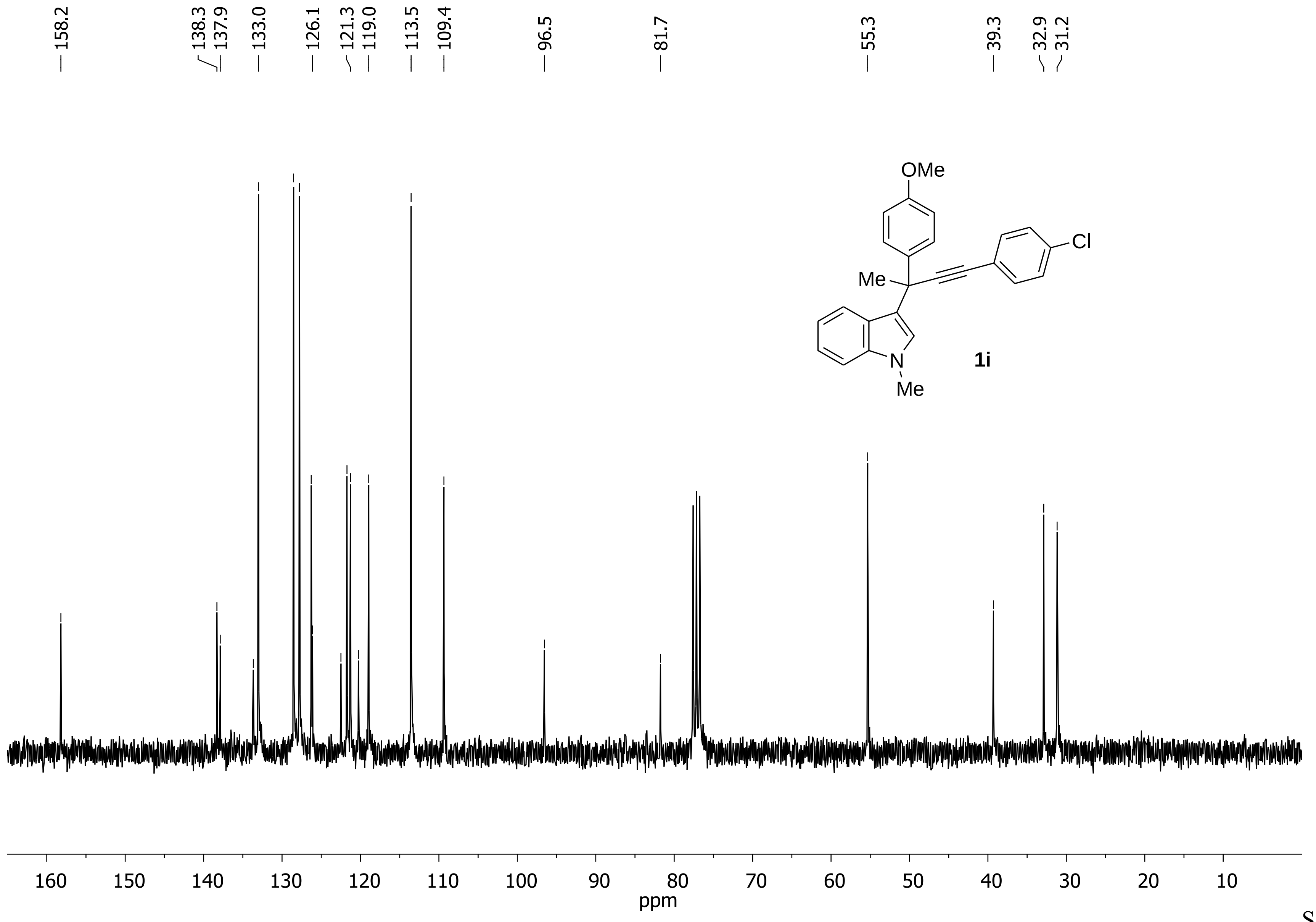

158.2
138.3
137.9
133.0
126.1
121.3
119.0
113.5
109.4
96.5
81.7
55.3
39.3
32.9
31.2
OMe
Cl
Me
N
Me
1i
160
150
140
130
120
110
100
90
80
70
60
50
40
30
20
10
ppm

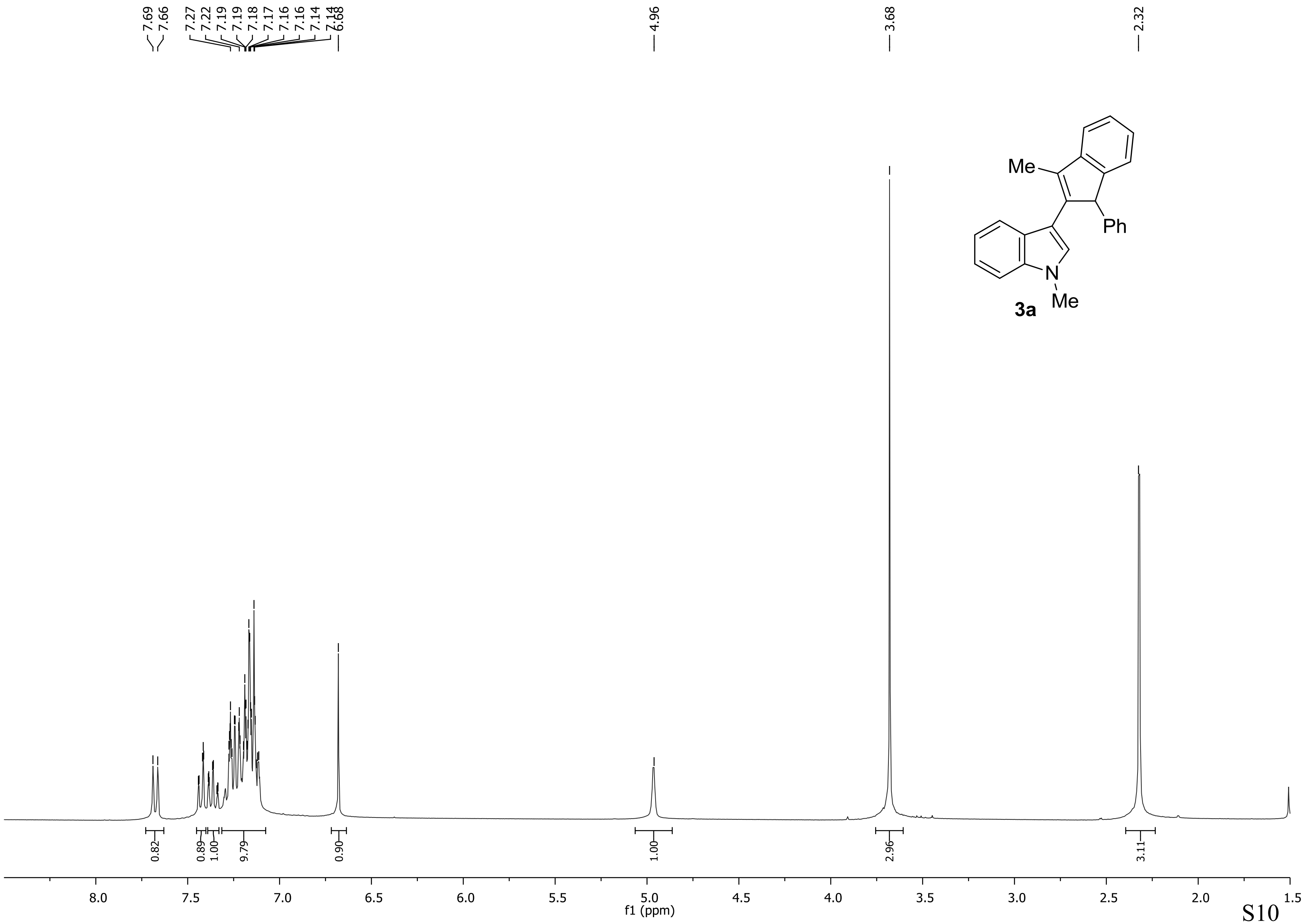
7.69
7.66
7.27
7.22
7.19
7.19
7.18
7.17
7.16
7.16
7.14
7.14
6.68
4.96
3.68
2.32
Me
Ph
N
Me
3a
0.82
0.89
1.00
9.79
0.90
1.00
2.96
3.11
8.0
7.5
7.0
6.5
6.0
5.5
5.0
4.5
4.0
3.5
3.0
2.5
2.0
1.5
f1 (ppm)

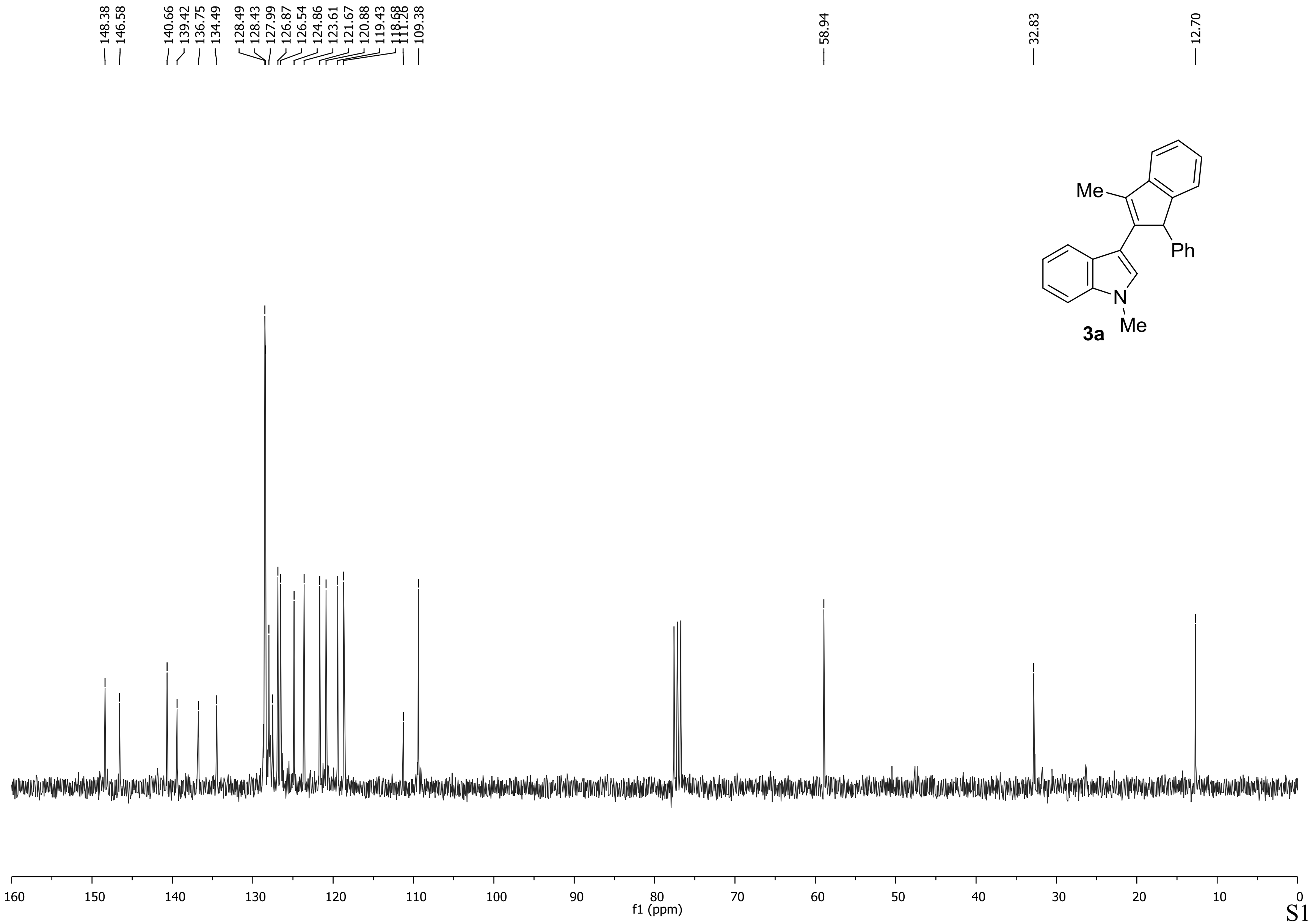
148.38
146.58
140.66
139.42
136.75
134.49
128.49
128.43
127.99
126.87
126.54
124.86
123.61
121.67
120.88
119.43
118.68
111.26
109.38
58.94
32.83
12.70
Me
Ph
N
Me
3a
160
150
140
130
120
110
100
90
80
70
60
50
40
30
20
10
0
f1 (ppm)

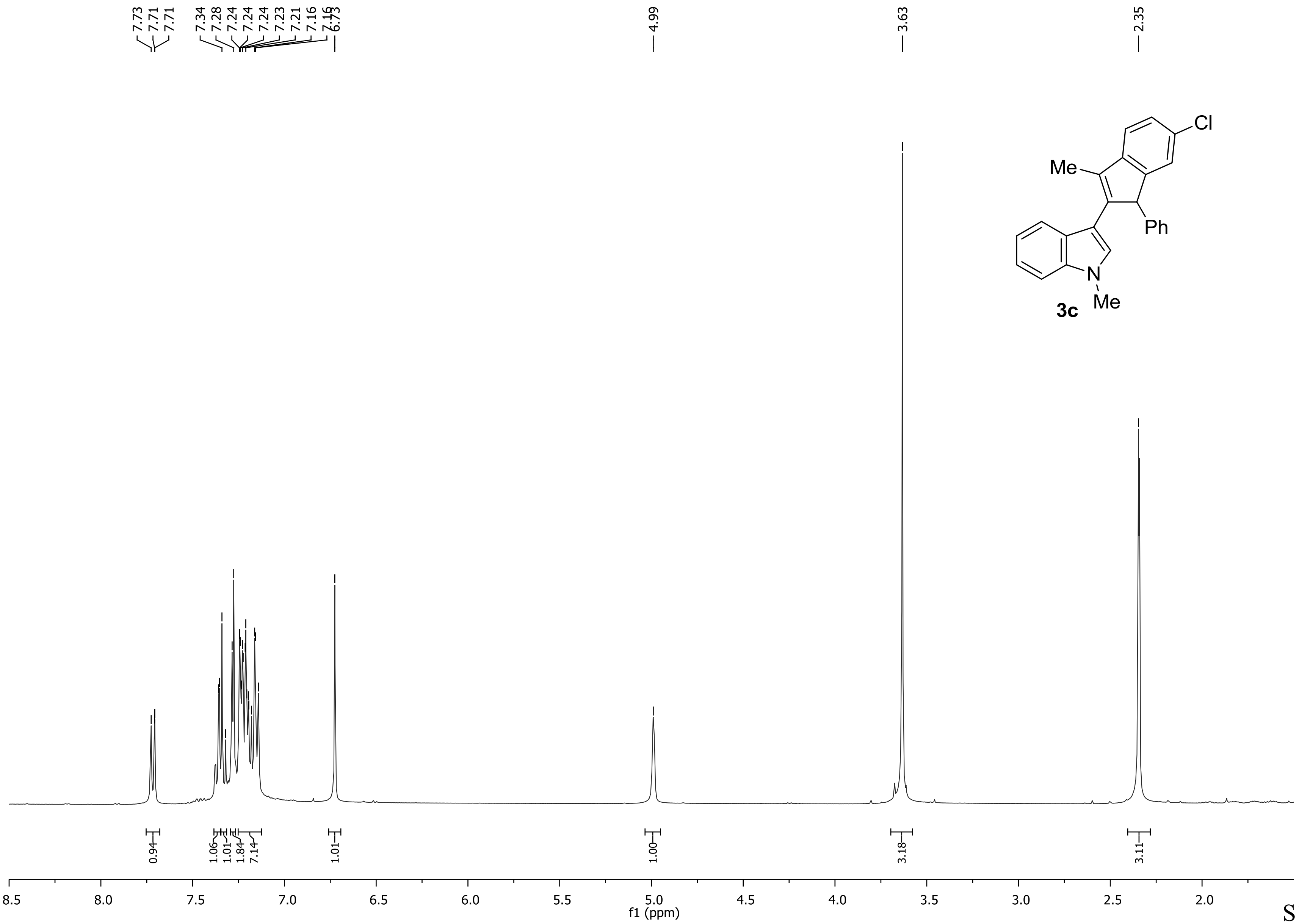

7.73
7.71
7.71
7.34
7.28
7.24
7.24
7.24
7.23
7.21
7.16
7.16
6.73
4.99
3.63
2.35
Cl
Me
Ph
N
Me
3c
0.94
1.06
1.01
1.84
7.14
1.01
1.00
3.18
3.11
8.5
8.0
7.5
7.0
6.5
6.0
5.5
5.0
4.5
4.0
3.5
3.0
2.5
2.0
f1 (ppm)

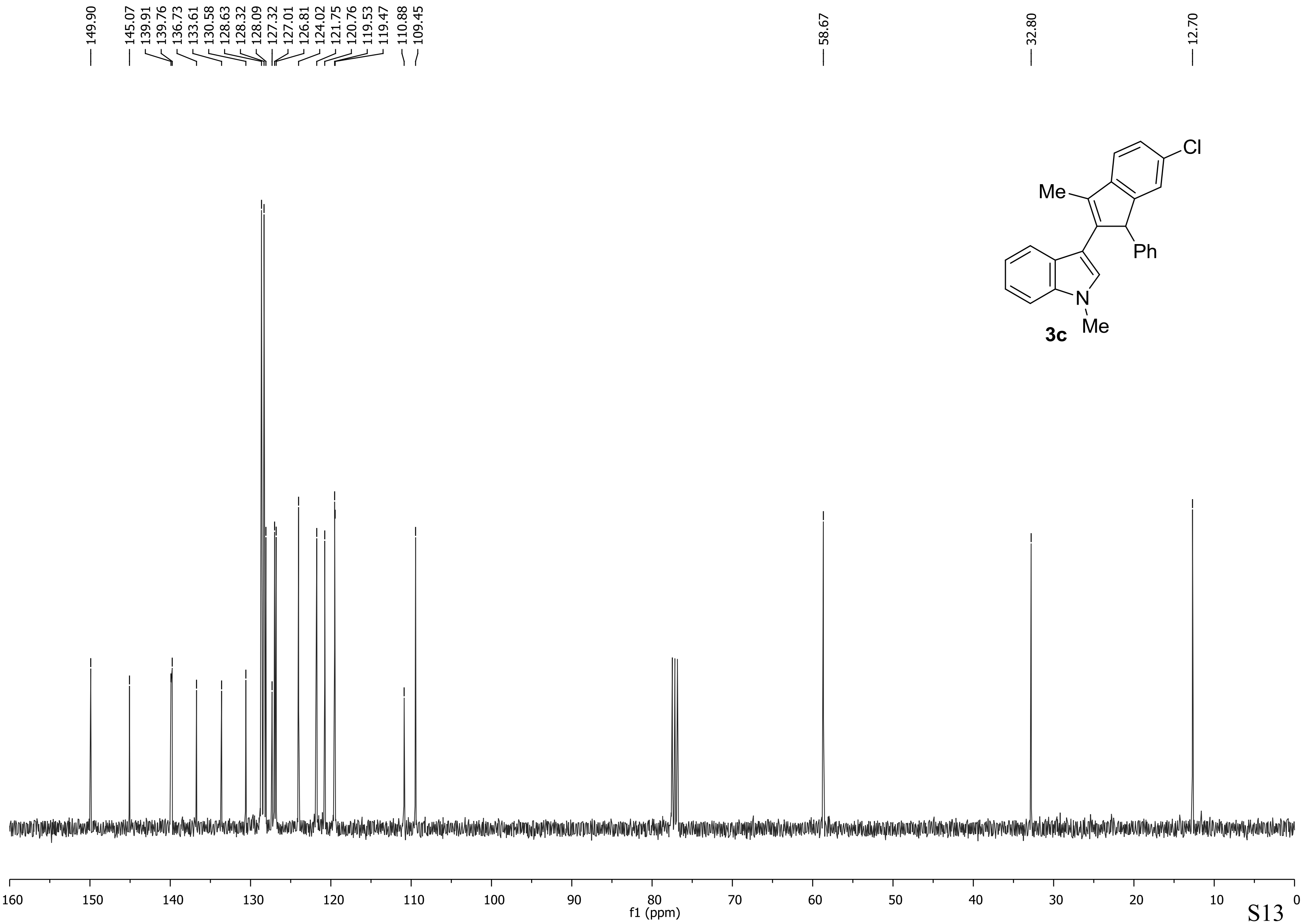
149.90
145.07
139.91
139.76
136.73
133.61
130.58
128.63
128.32
128.09
127.32
127.01
126.81
124.02
121.75
120.76
119.53
119.47
110.88
109.45
58.67
32.80
12.70
Cl
Me
Ph
N
Me
3c
160
150
140
130
120
110
100
90
80
70
60
50
40
30
20
10
0
f1 (ppm)

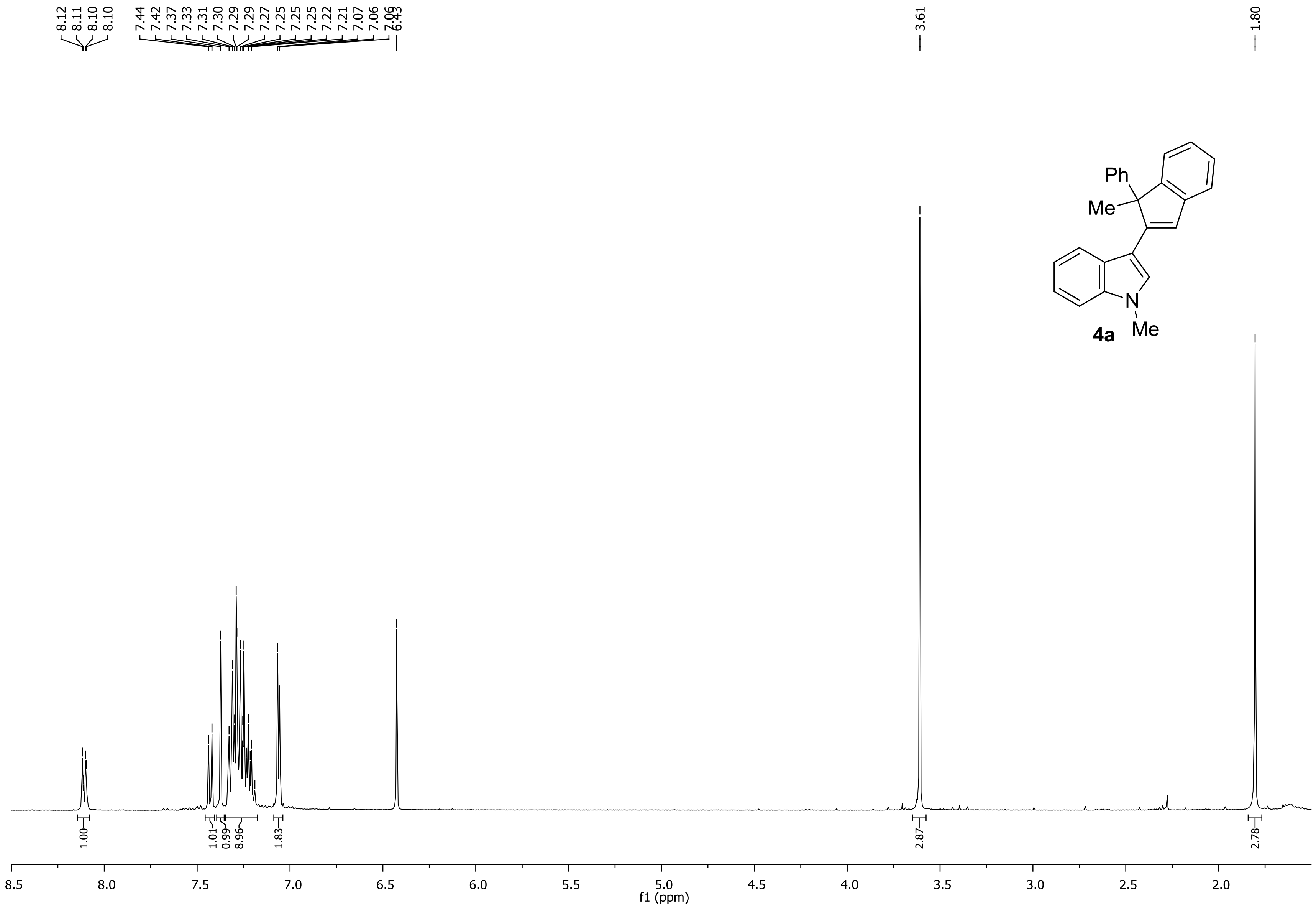
8.12
8.11
8.10
8.10
7.44
7.42
7.37
7.33
7.31
7.30
7.29
7.29
7.27
7.25
7.25
7.25
7.22
7.21
7.07
7.06
6.43
3.61
1.80
Ph
Me
N
Me
4a
1.00
1.01
0.99
8.96
1.83
2.87
2.78
f1 (ppm)

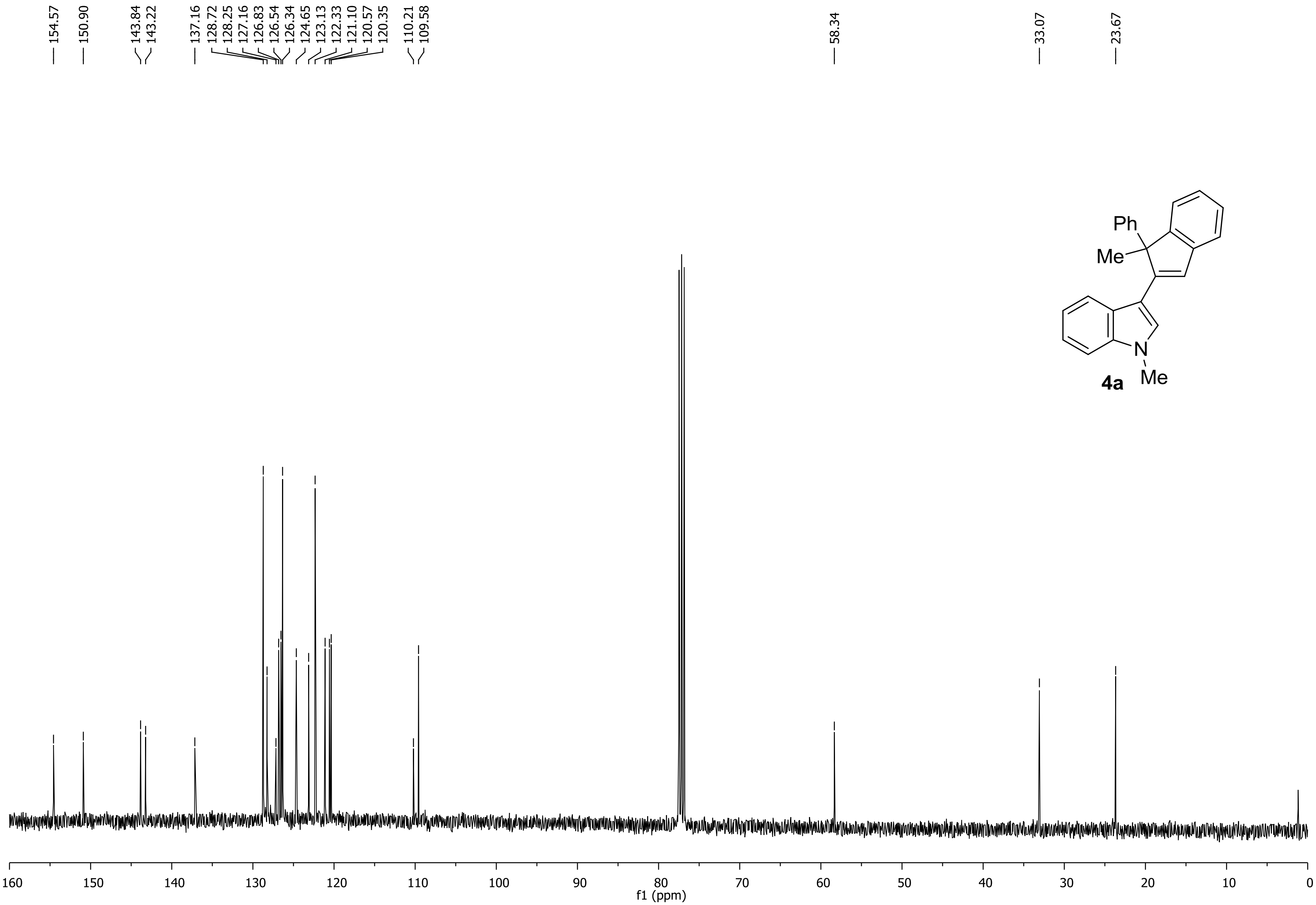
154.57
150.90
143.84
143.22
137.16
128.72
128.25
127.16
126.83
126.54
126.34
124.65
123.13
122.33
121.10
120.57
120.35
110.21
109.58
58.34
33.07
23.67
Ph
Me
N
Me
4a
160 150 140 130 120 110 100 90 80 70 60 50 40 30 20 10 0
f1 (ppm)

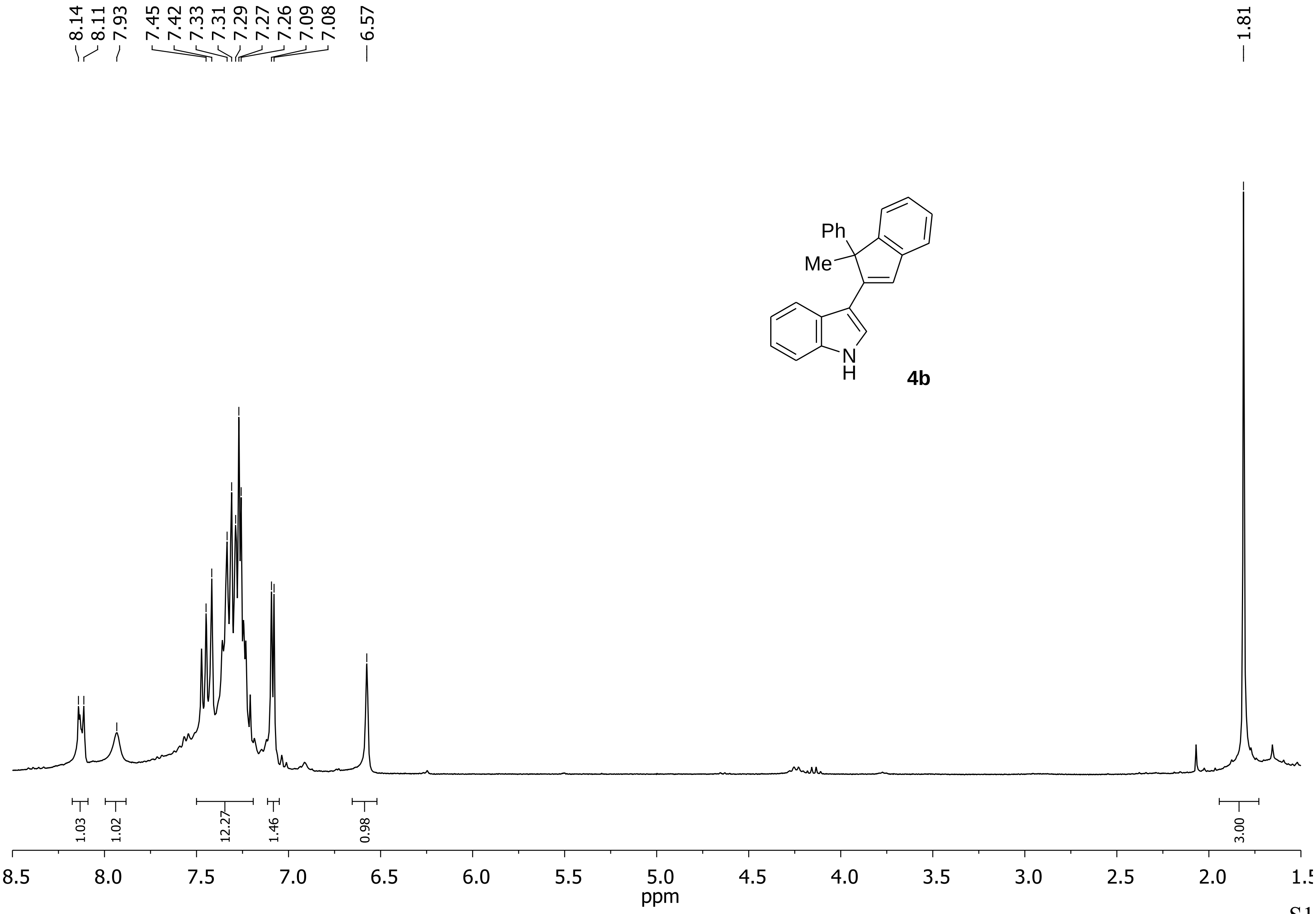
8.14
8.11
7.93
7.45
7.42
7.33
7.31
7.29
7.27
7.26
7.09
7.08
6.57
1.81
Ph
Me
N
H
4b
1.03
1.02
12.27
1.46
0.98
3.00
8.5
8.0
7.5
7.0
6.5
6.0
5.5
5.0
4.5
4.0
3.5
3.0
2.5
2.0
ppm

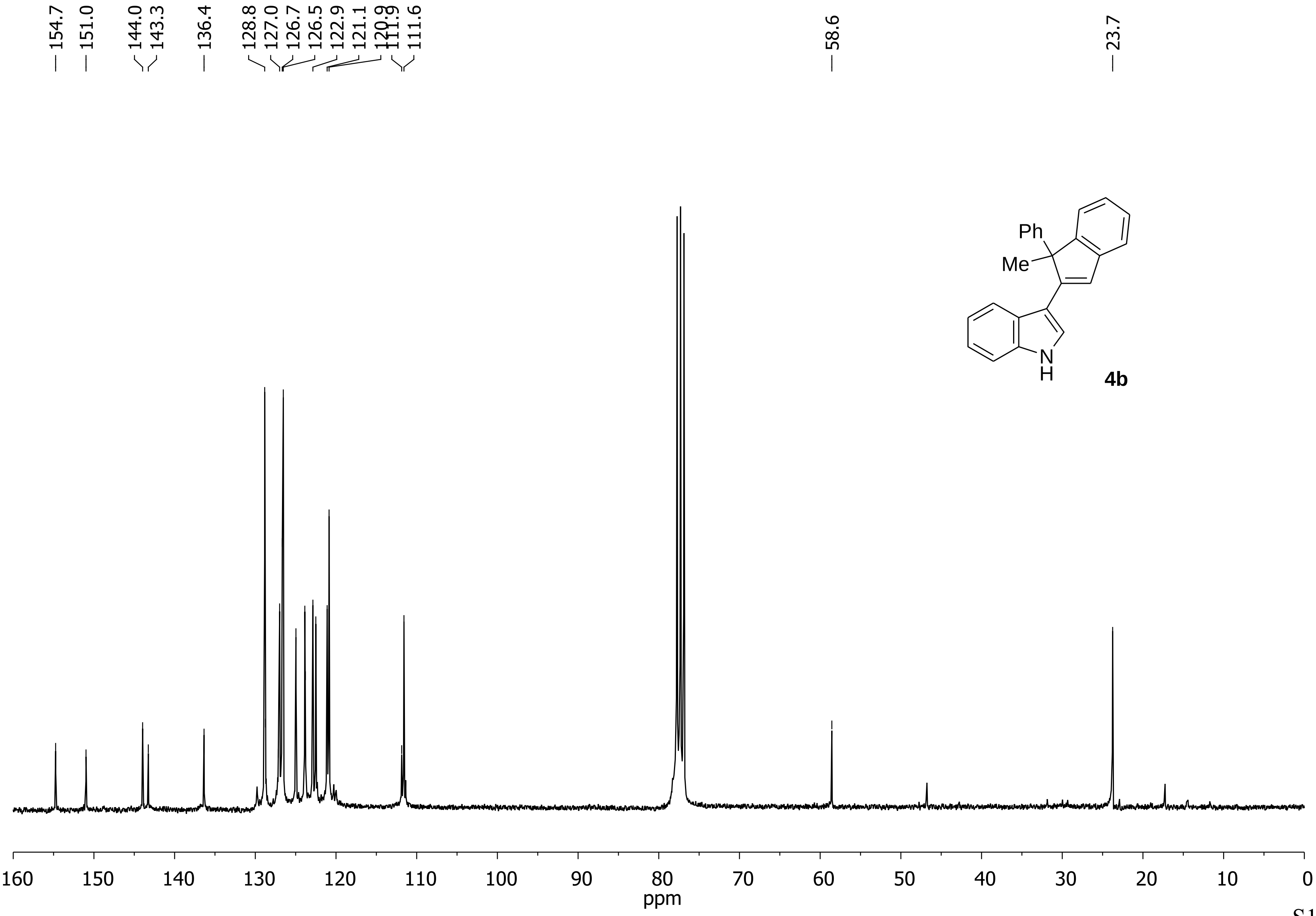
154.7
151.0
144.0
143.3
136.4
128.8
127.0
126.7
126.5
122.9
121.1
120.9
111.9
111.6
58.6
23.7
Ph
Me
N
H
4b
160
150
140
130
120
110
100
90
80
70
60
50
40
30
20
10
0
ppm

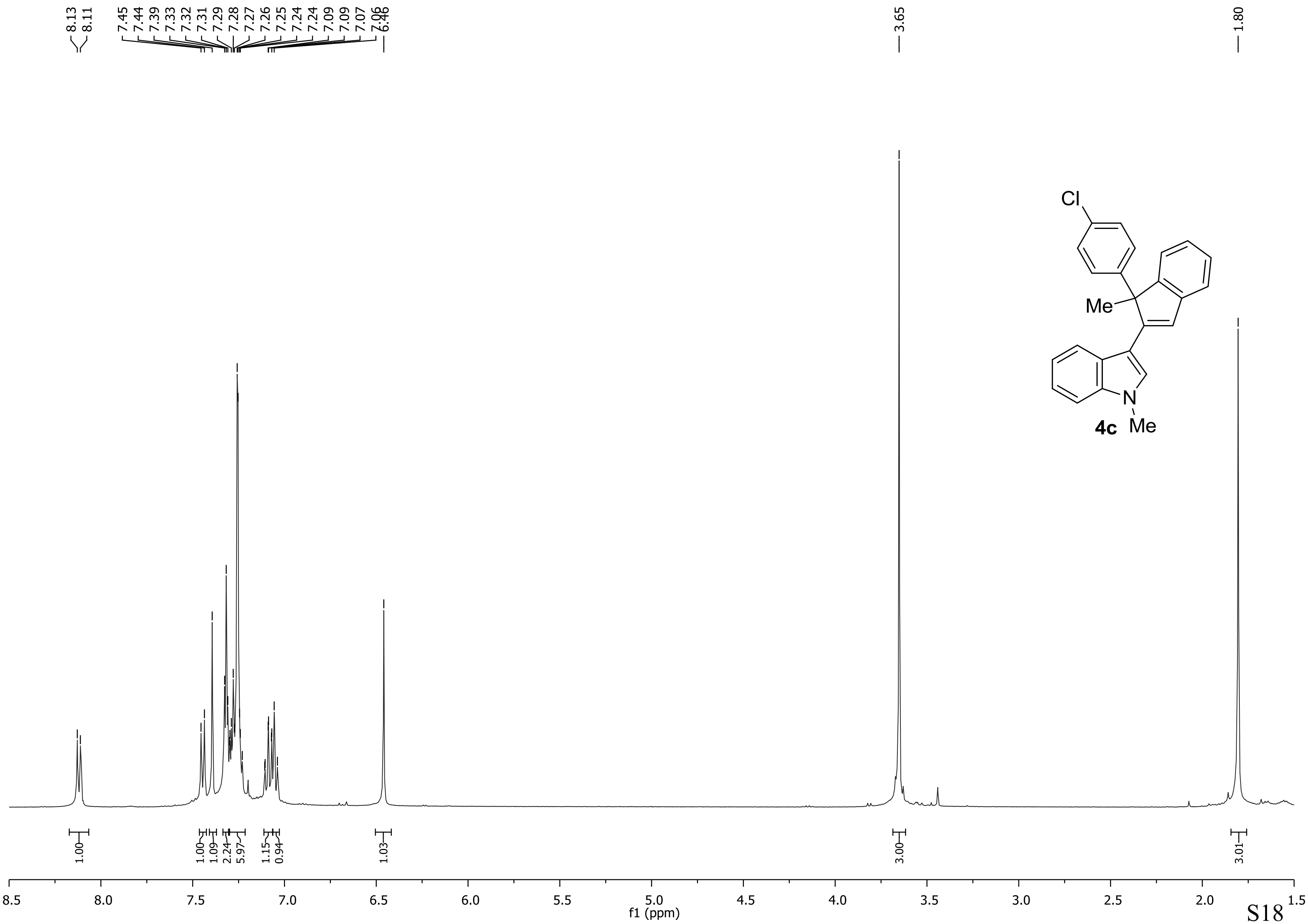
8.13
8.11
7.45
7.44
7.39
7.33
7.32
7.31
7.29
7.28
7.27
7.26
7.25
7.24
7.24
7.09
7.09
7.07
7.06
6.46
3.65
1.80
Cl
Me
N
Me
4c
1.00
1.00
1.09
2.24
5.97
1.15
0.94
1.03
3.00
3.01
8.5
8.0
7.5
7.0
6.5
6.0
5.5
5.0
4.5
4.0
3.5
3.0
2.5
2.0
1.5
f1 (ppm)

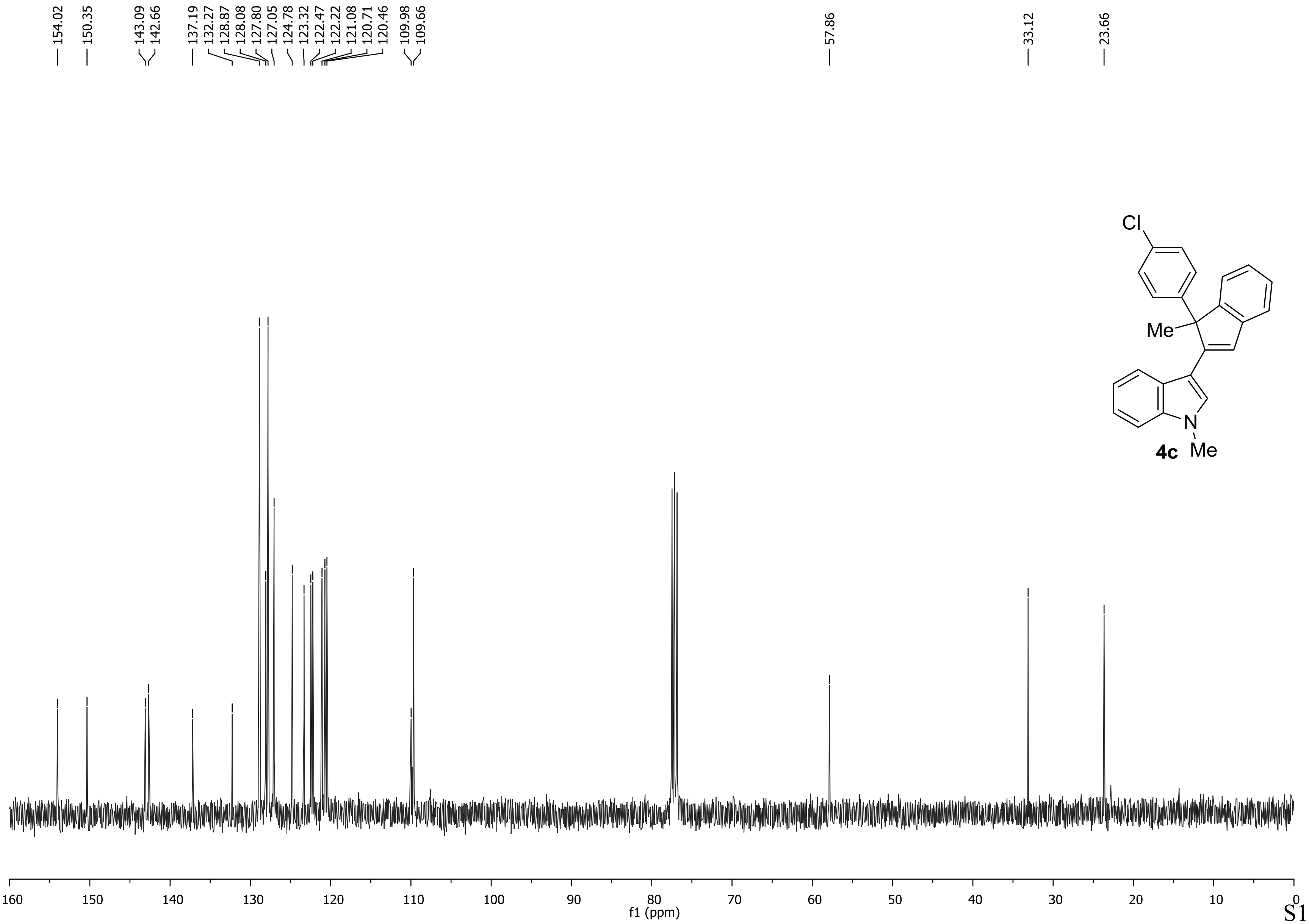
154.02
150.35
143.09
142.66
137.19
132.27
128.87
128.08
127.80
127.05
124.78
123.32
122.47
122.22
121.08
120.71
120.46
109.98
109.66
57.86
33.12
23.66
Cl
Me
N
Me
4c
160 150 140 130 120 110 100 90 80 70 60 50 40 30 20 10 0
f1 (ppm)

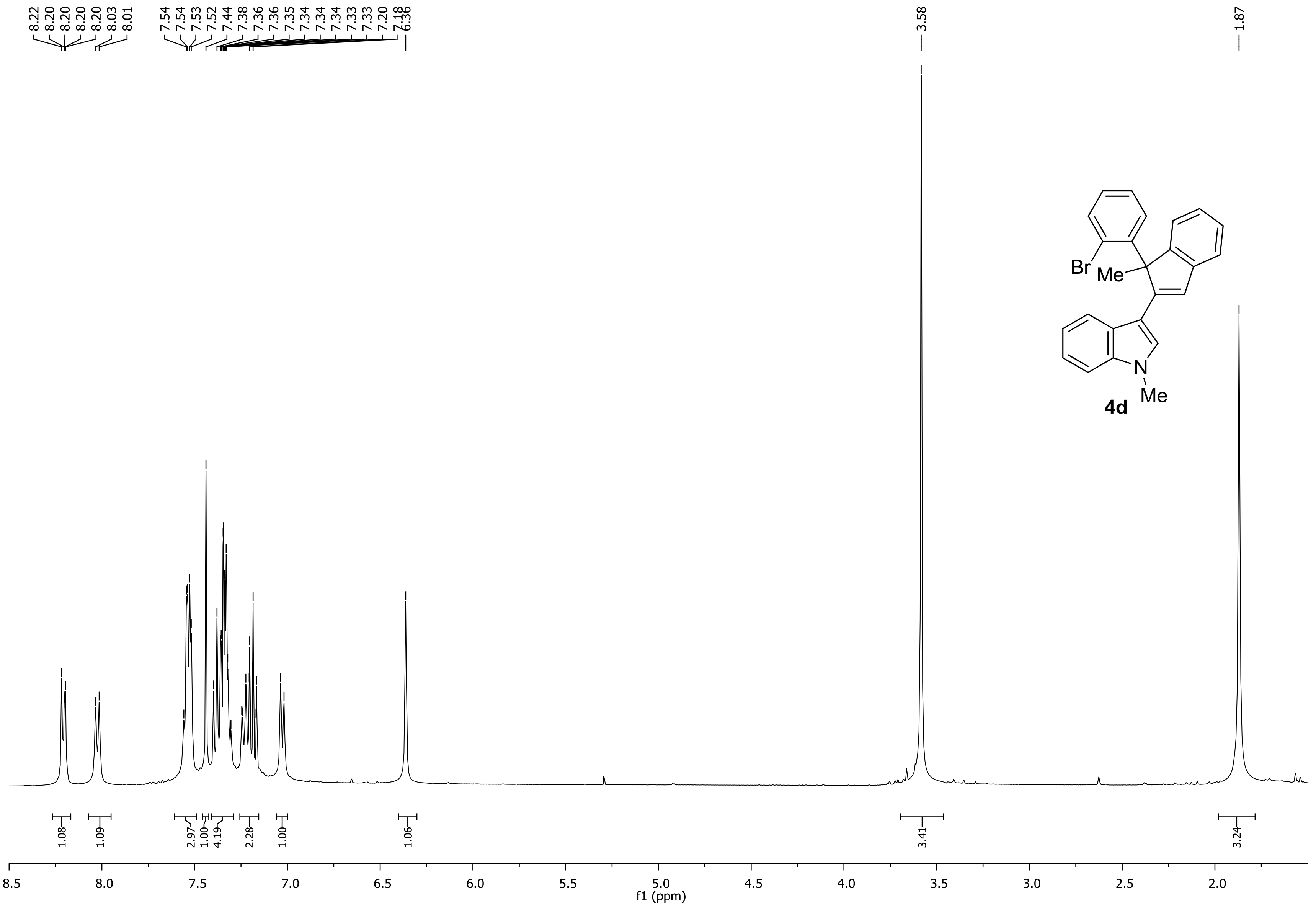

8.22
8.20
8.20
8.20
8.20
8.03
8.01
7.54
7.54
7.53
7.52
7.44
7.38
7.36
7.36
7.35
7.34
7.34
7.34
7.33
7.33
7.20
7.18
6.36
3.58
1.87
Br
Me
N
Me
4d
1.08
1.09
2.97
1.00
4.19
2.28
1.00
1.06
3.41
3.24
8.5
8.0
7.5
7.0
6.5
6.0
5.5
5.0
4.5
4.0
3.5
3.0
2.5
2.0
f1 (ppm)

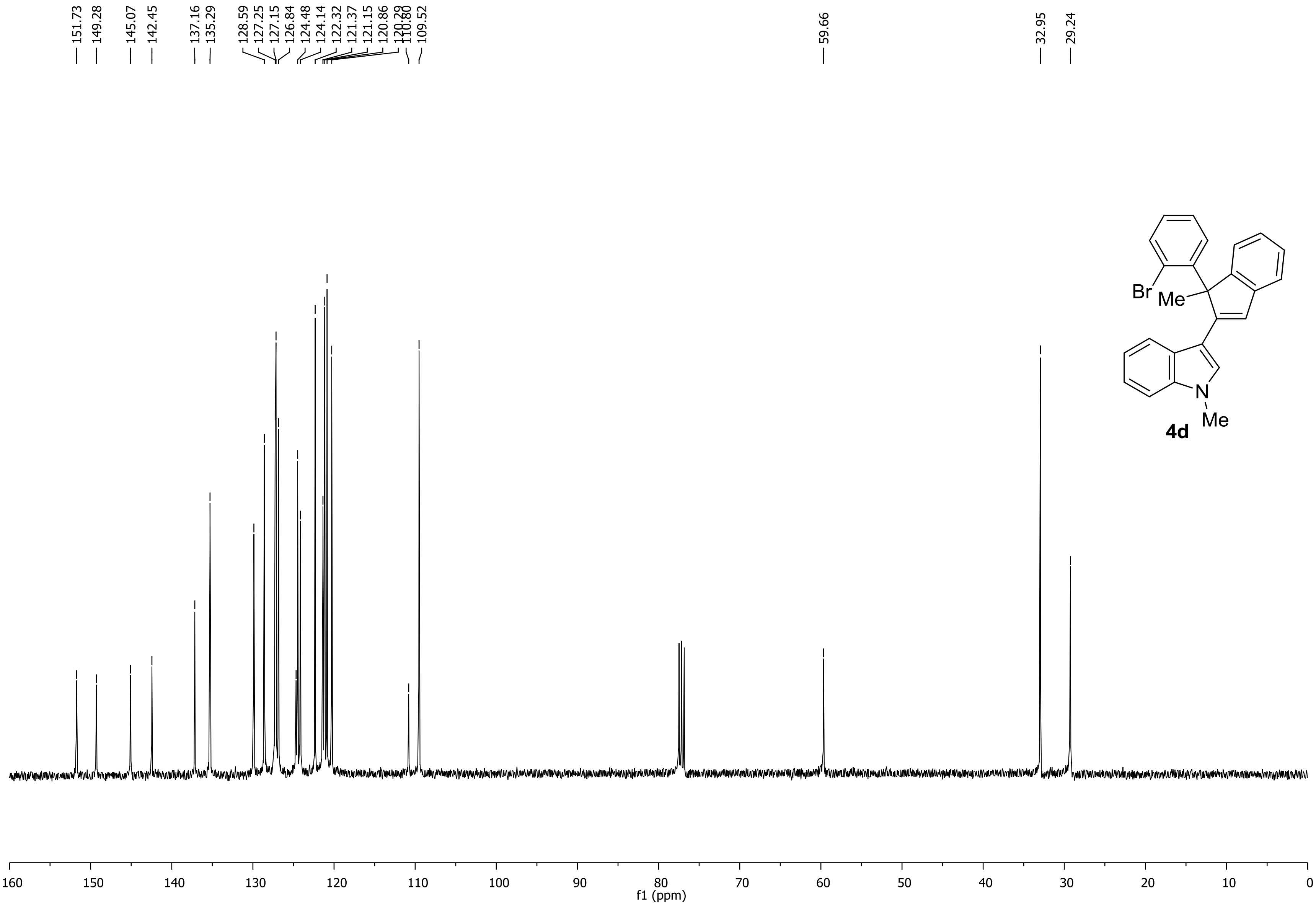
151.73
149.28
145.07
142.45
137.16
135.29
128.59
127.25
127.15
126.84
124.48
124.14
122.32
121.37
121.15
120.86
120.29
110.80
109.52
59.66
32.95
29.24
Br
Me
N
Me
4d
f1 (ppm)

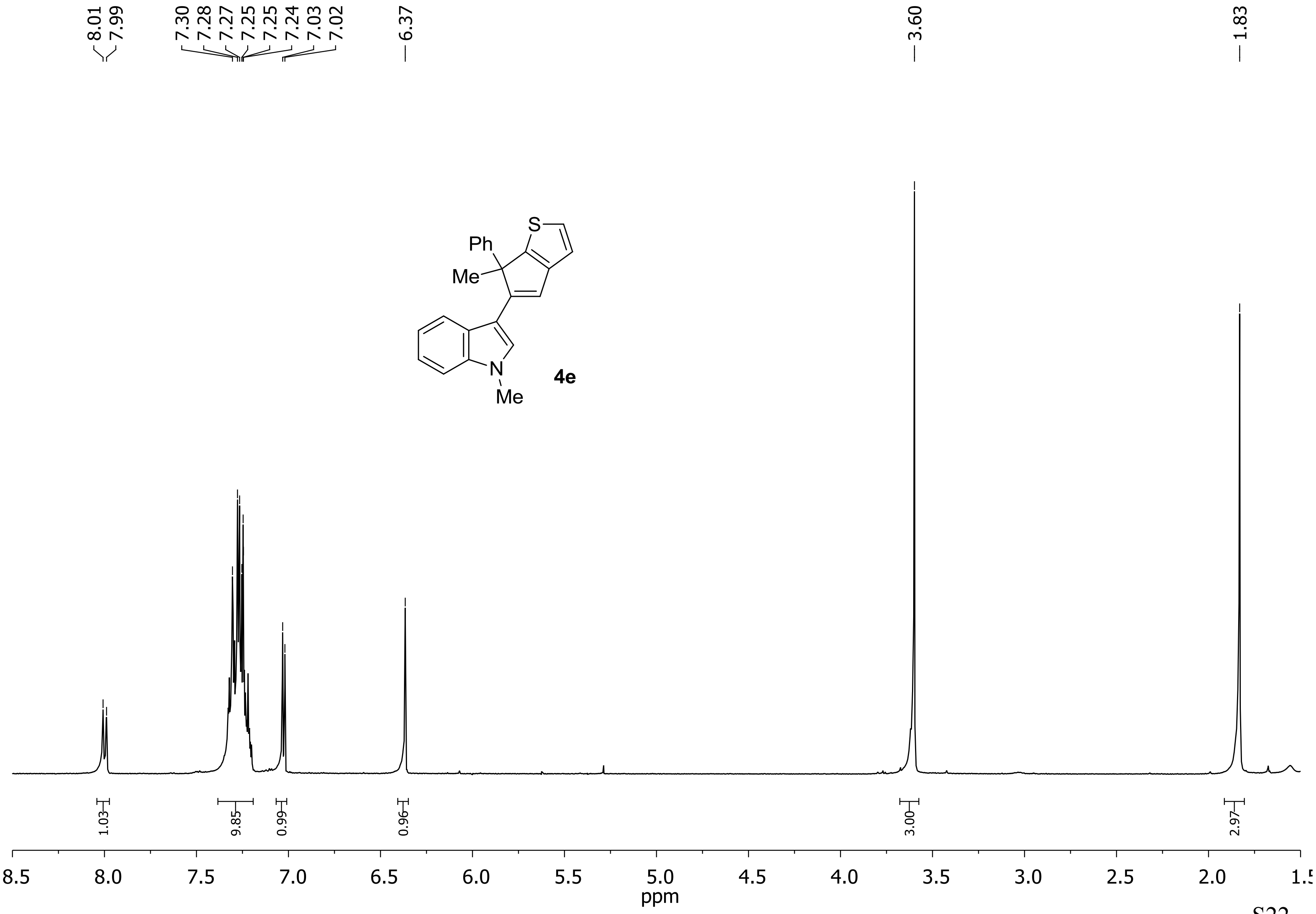
8.01
7.99
7.30
7.28
7.27
7.25
7.25
7.24
7.03
7.02
6.37
3.60
1.83
Ph
S
Me
N
Me
4e
1.03
9.85
0.99
0.96
3.00
2.97
8.5
8.0
7.5
7.0
6.5
6.0
5.5
5.0
4.5
4.0
3.5
3.0
2.5
2.0
ppm

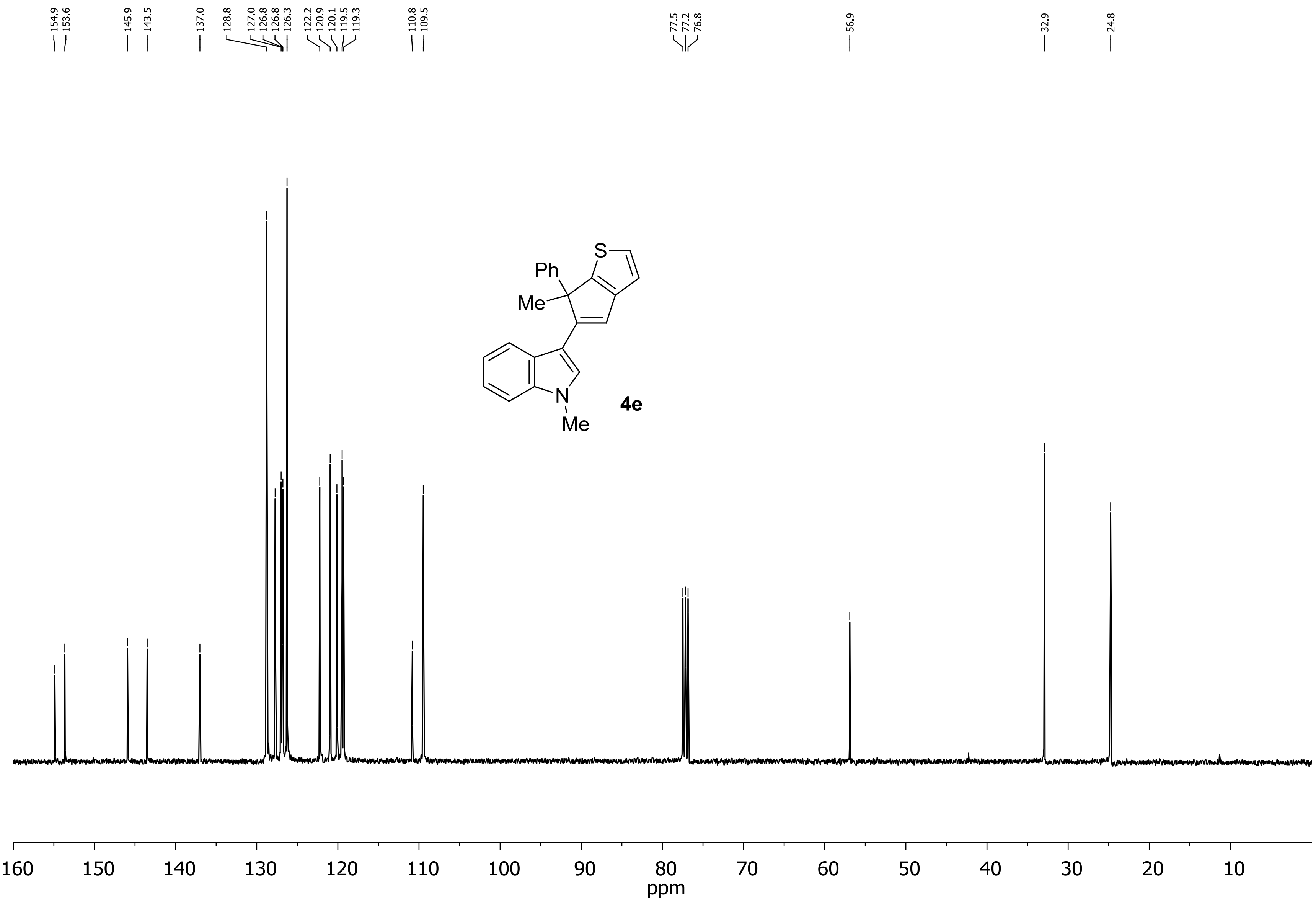
154.9
153.6
145.9
143.5
137.0
128.8
127.0
126.8
126.8
126.3
122.2
120.9
120.1
119.5
119.3
110.8
109.5
77.5
77.2
76.8
56.9
32.9
24.8
S
Ph
Me
N
Me
4e
160
150
140
130
120
110
100
90
80
70
60
50
40
30
20
10
ppm

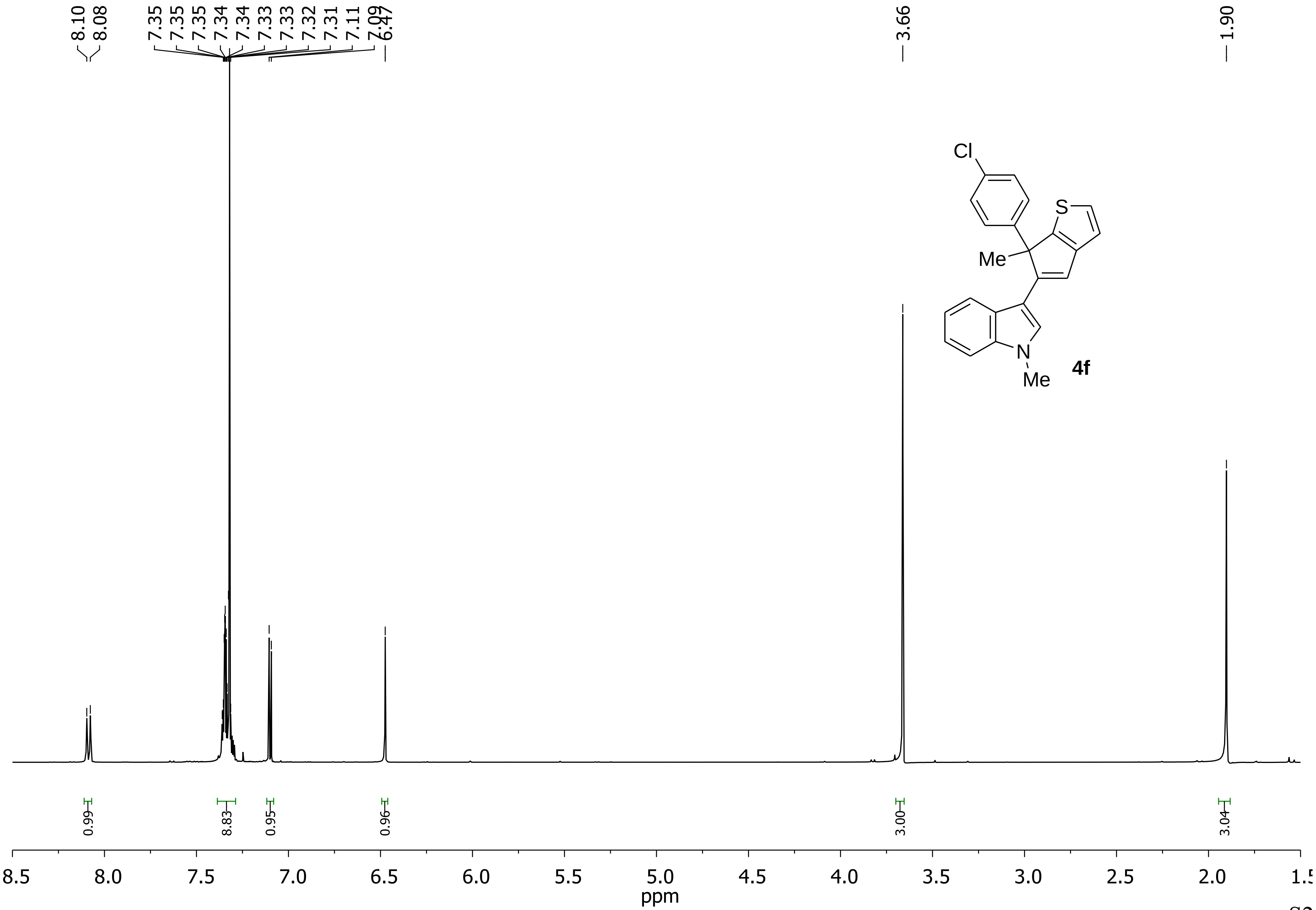

8.10
8.08
7.35
7.35
7.35
7.34
7.34
7.33
7.33
7.32
7.31
7.11
7.09
6.47
3.66
1.90
0.99
8.83
0.95
0.96
3.00
3.04
8.5
8.0
7.5
7.0
6.5
6.0
5.5
5.0
4.5
4.0
3.5
3.0
2.5
2.0
ppm
Cl
S
Me
N
Me

4f

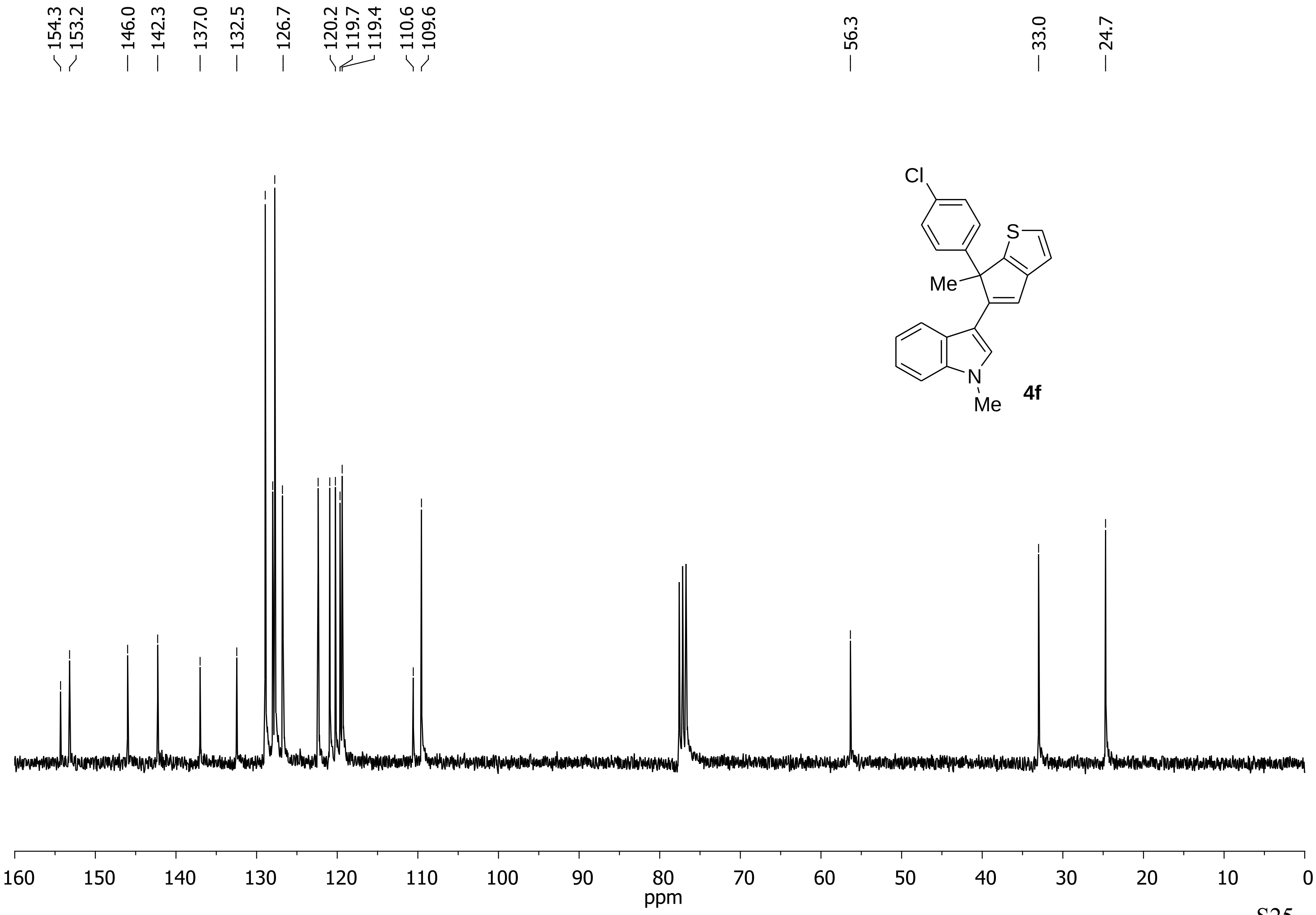

154.3
153.2
146.0
142.3
137.0
132.5
126.7
120.2
119.7
119.4
110.6
109.6
56.3
33.0
24.7
Cl
S
Me
N
Me
4f
160
150
140
130
120
110
100
90
80
70
60
50
40
30
20
10
0
ppm

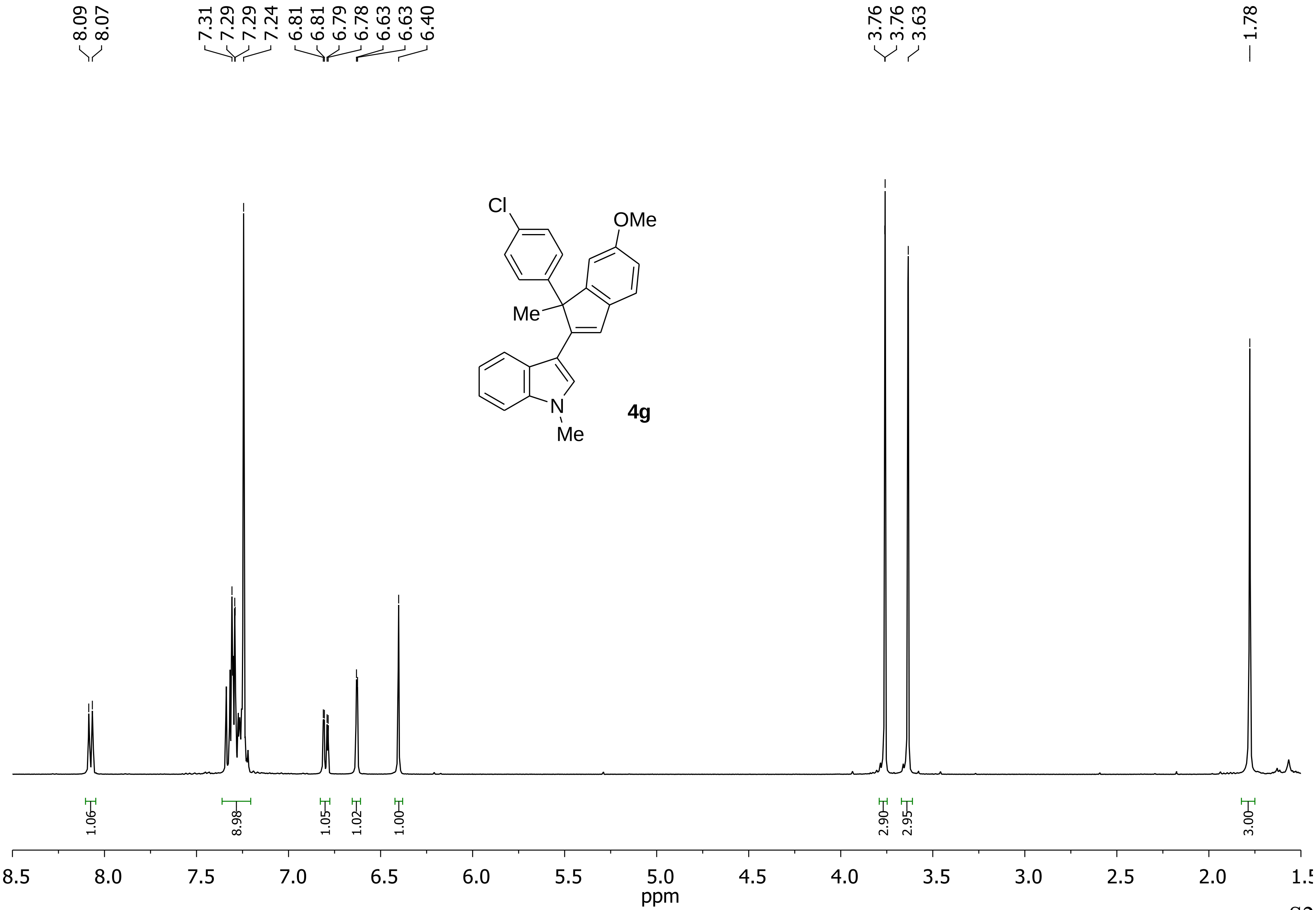
8.09
8.07
7.31
7.29
7.29
7.24
6.81
6.81
6.79
6.78
6.63
6.63
6.63
6.40
3.76
3.76
3.63
1.78
Cl
OMe
Me
N
Me
4g
1.06
8.98
1.05
1.02
1.00
2.90
2.95
3.00
8.5
8.0
7.5
7.0
6.5
6.0
5.5
5.0
4.5
4.0
3.5
3.0
2.5
2.0
ppm

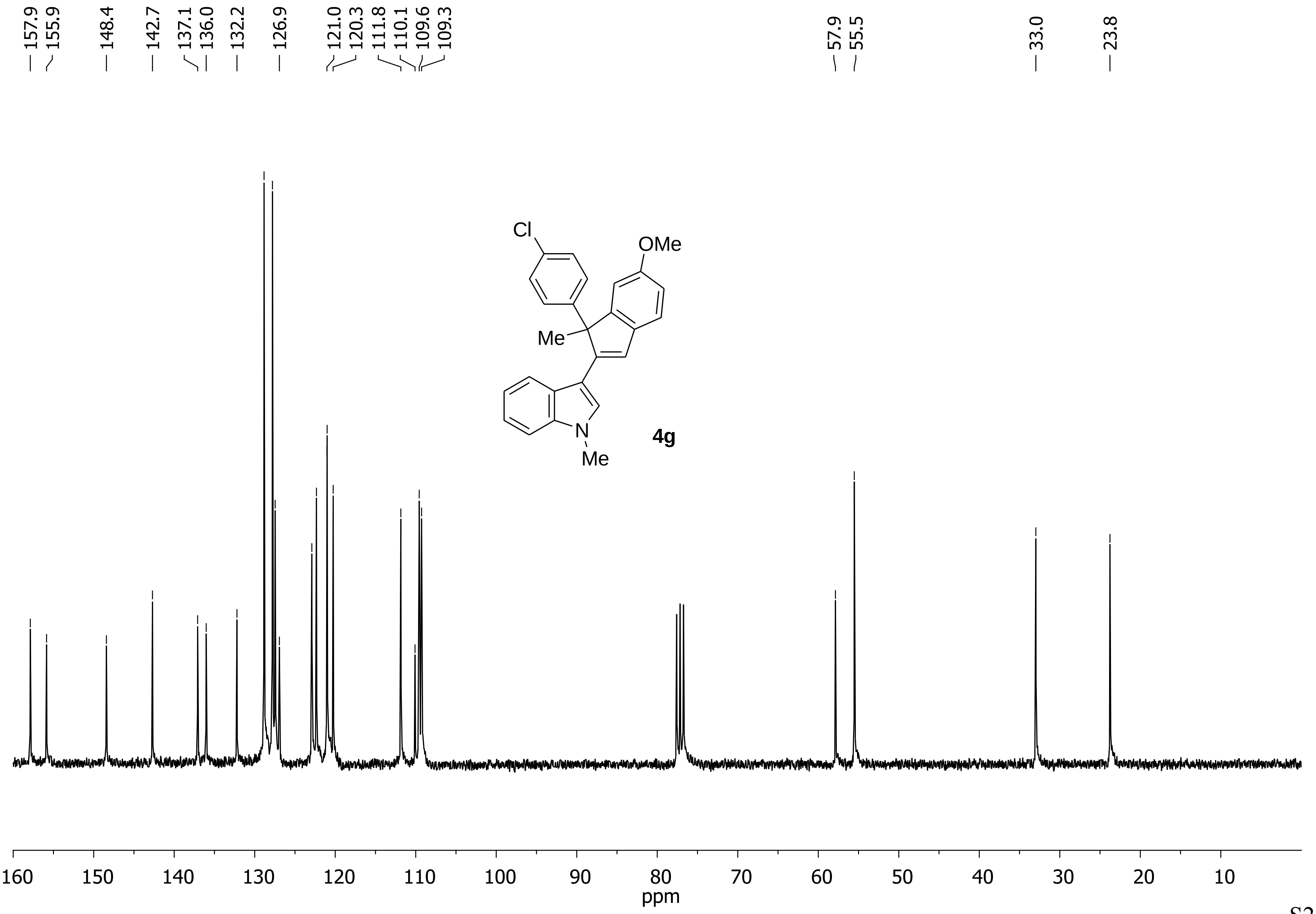
157.9
155.9
148.4
142.7
137.1
136.0
132.2
126.9
121.0
120.3
111.8
110.1
109.6
109.3
57.9
55.5
33.0
23.8
Cl
OMe
Me
N
Me
4g
160
150
140
130
120
110
100
90
80
70
60
50
40
30
20
10
ppm

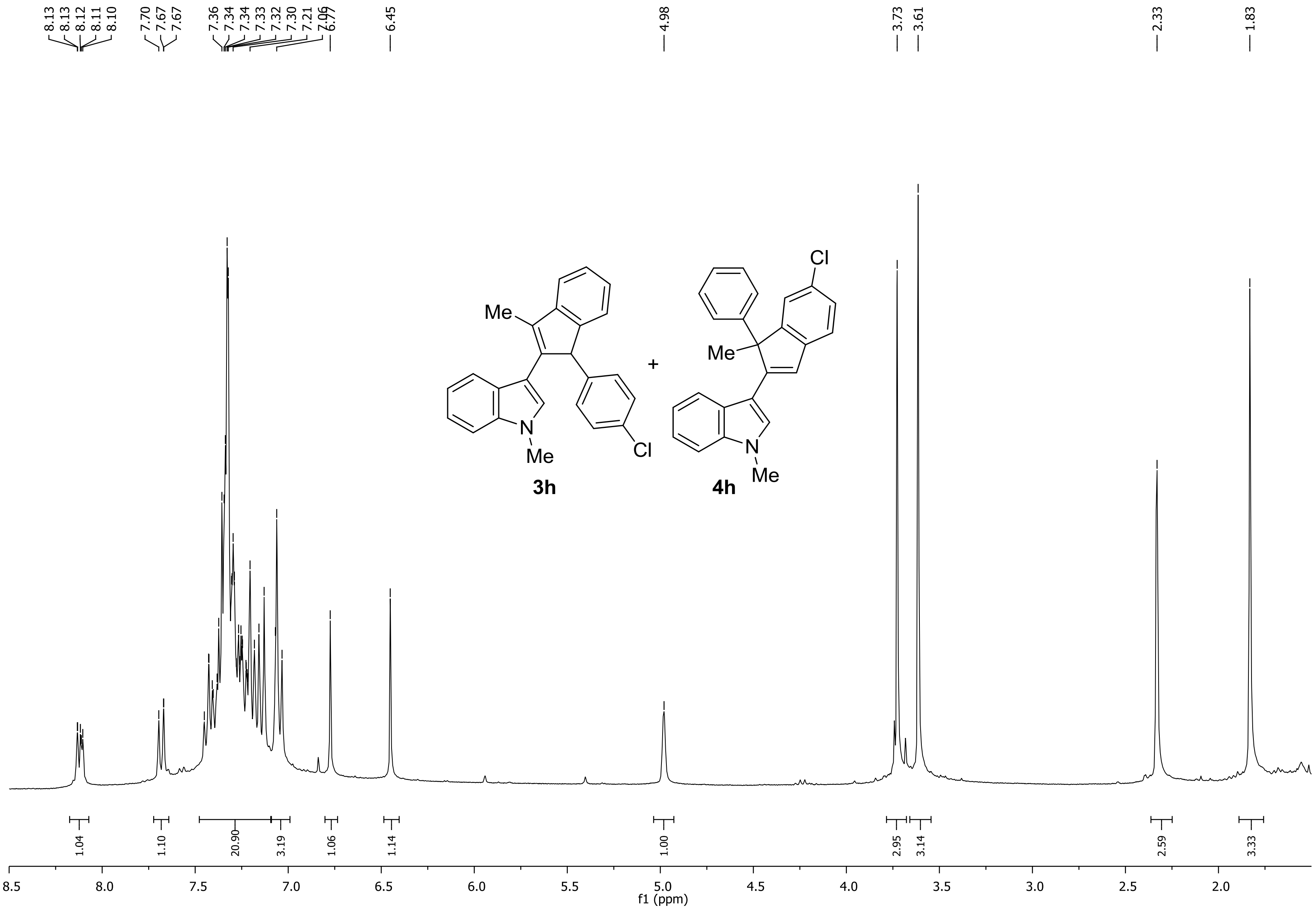
8.13
8.13
8.12
8.11
8.10
7.70
7.67
7.67
7.36
7.34
7.34
7.33
7.32
7.30
7.21
7.06
6.77
6.45
4.98
3.73
3.61
2.33
1.83
Me
N
Me
Cl
3h
+
Cl
Me
N
Me
4h
1.04
1.10
20.90
3.19
1.06
1.14
1.00
2.95
3.14
2.59
3.33
8.5
8.0
7.5
7.0
6.5
6.0
5.5
5.0
4.5
4.0
3.5
3.0
2.5
2.0
f1 (ppm)

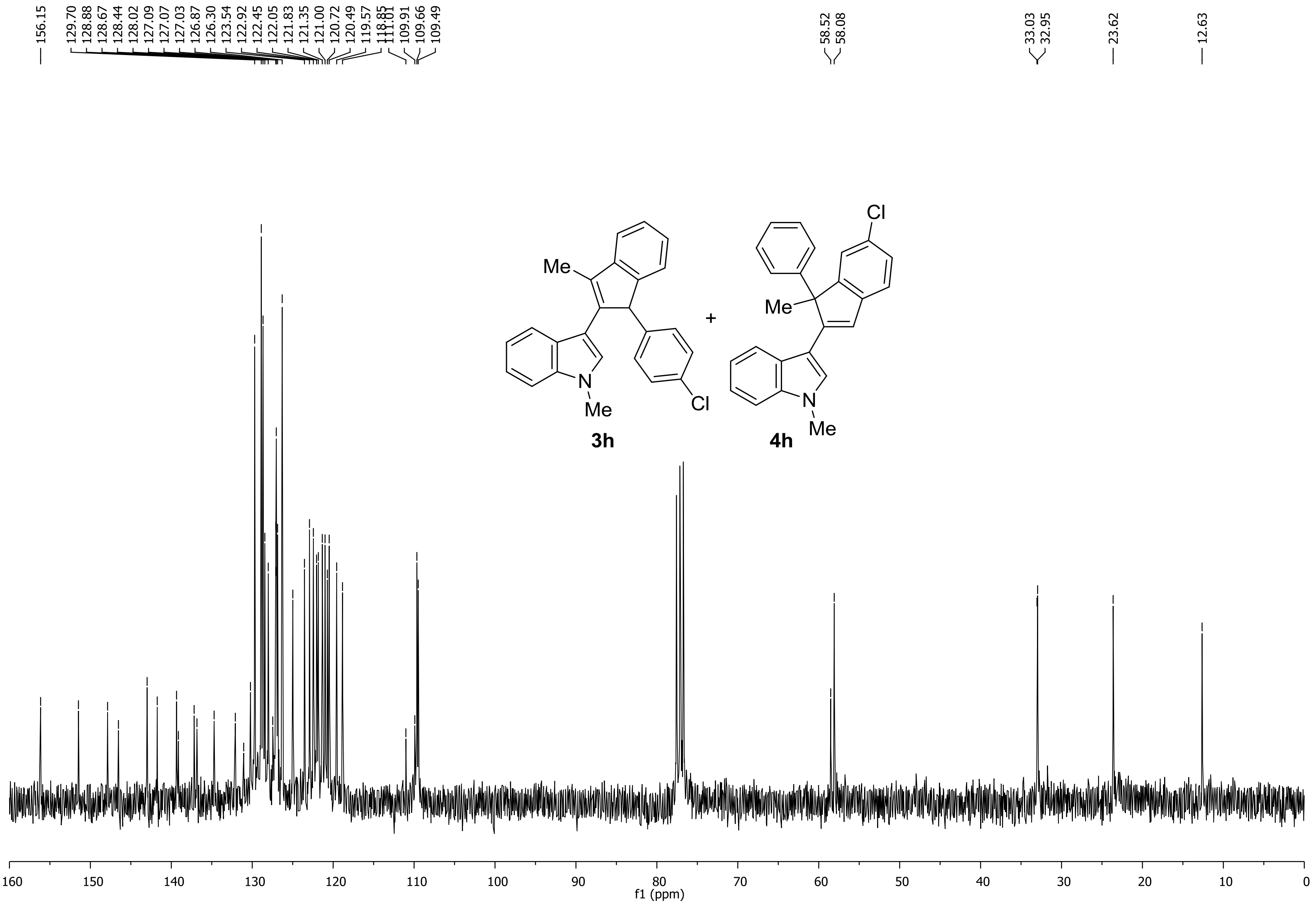
156.15
129.70
128.88
128.67
128.44
128.02
127.09
127.07
127.03
126.87
126.30
123.54
122.92
122.45
122.05
121.83
121.35
121.00
120.72
120.49
119.57
118.85
111.01
109.91
109.66
109.49
58.52
58.08
33.03
32.95
23.62
12.63
Me
N
Me
Cl
3h
+
Cl
Me
N
Me
4h
f1 (ppm)

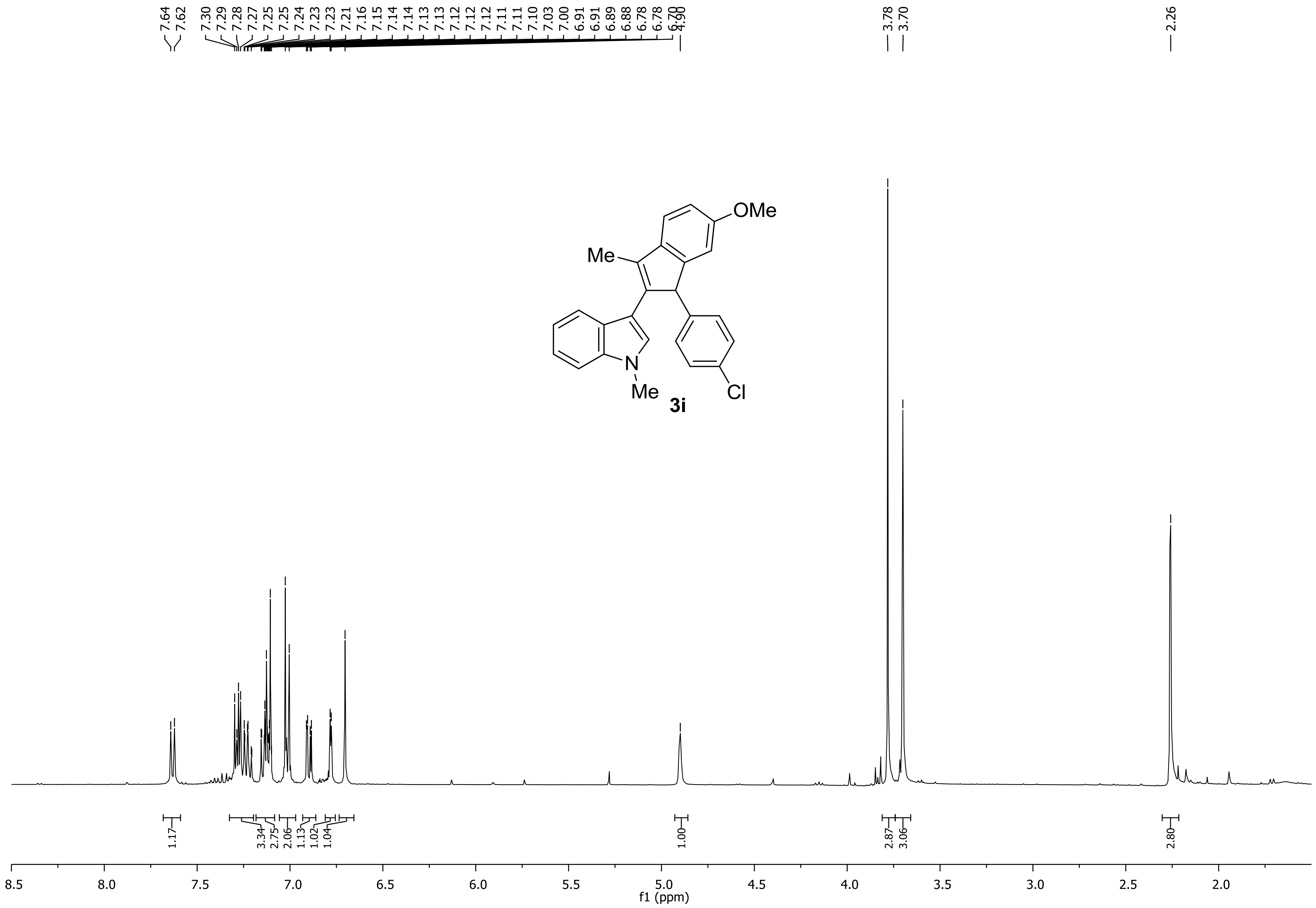

7.64
7.62
7.30
7.29
7.28
7.27
7.25
7.25
7.24
7.23
7.23
7.21
7.16
7.15
7.14
7.14
7.13
7.13
7.12
7.12
7.12
7.11
7.11
7.10
7.03
7.00
6.91
6.91
6.89
6.88
6.78
6.78
6.70
4.90
3.78
3.70
2.26
OMe
Me
N
Me
3i
Cl
1.17
3.34
2.75
2.06
1.13
1.02
1.04
1.00
2.87
3.06
2.80
8.5
8.0
7.5
7.0
6.5
6.0
5.5
5.0
4.5
4.0
3.5
3.0
2.5
2.0
f1 (ppm)

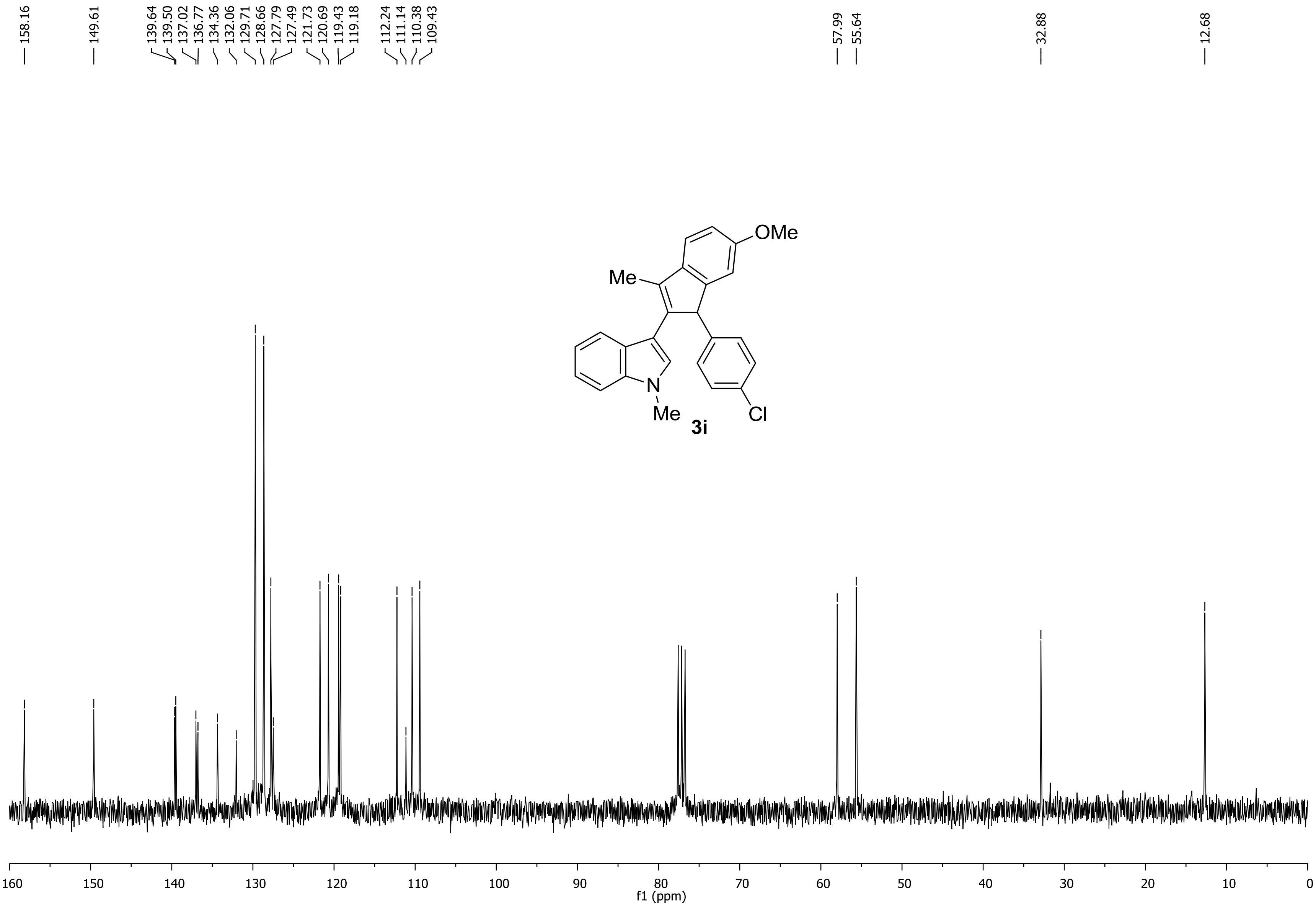
158.16
149.61
139.64
139.50
137.02
136.77
134.36
132.06
129.71
128.66
127.79
127.49
121.73
120.69
119.43
119.18
112.24
111.14
110.38
109.43
57.99
55.64
32.88
12.68
OMe
Me
N
Me
3i
Cl
160
150
140
130
120
110
100
90
80
70
60
50
40
30
20
10
0
f1 (ppm)

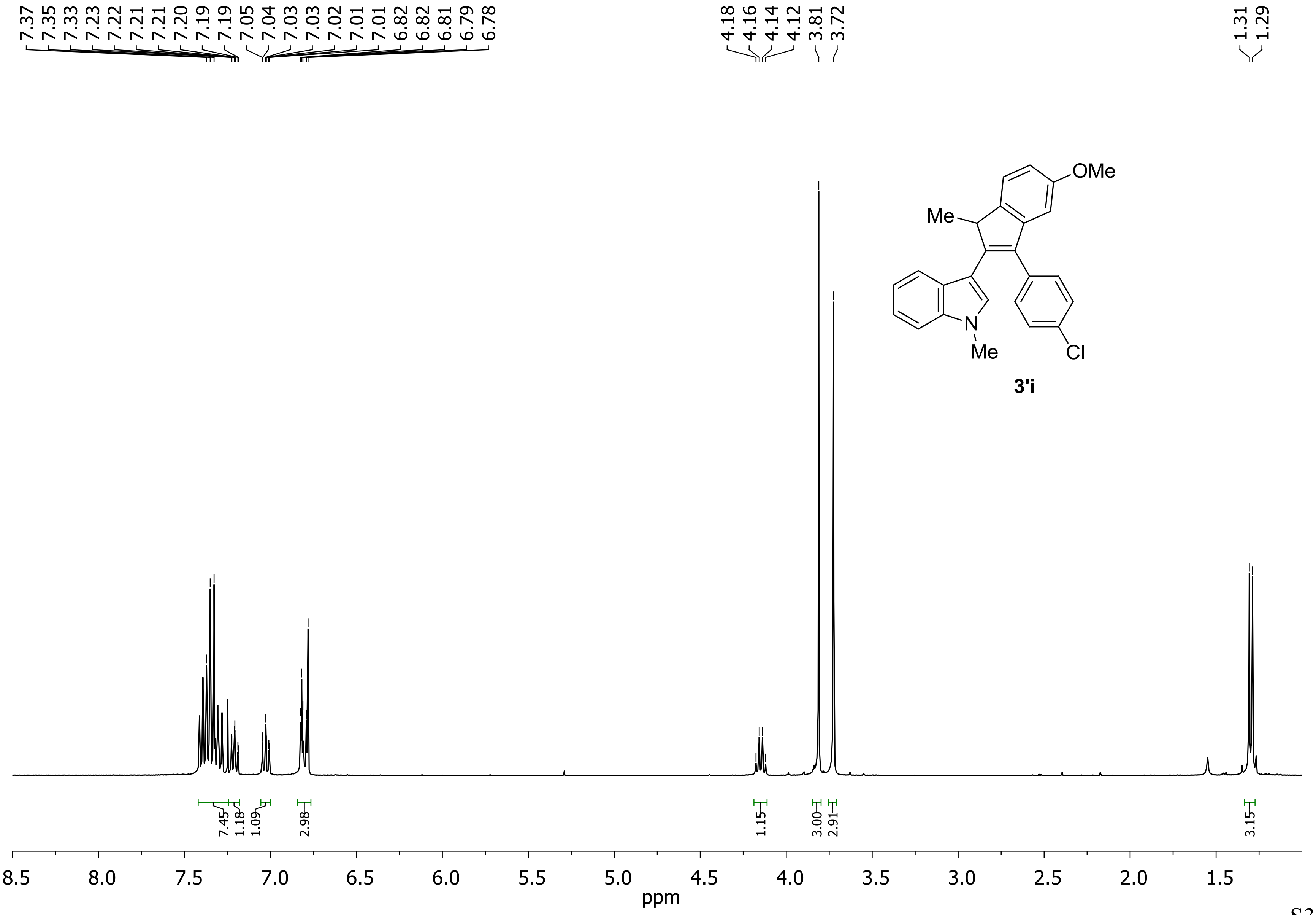
7.37 7.35 7.33 7.23 7.22 7.21 7.21 7.20 7.19 7.19 7.05 7.04 7.03 7.03 7.02 7.01 7.01 6.82 6.82 6.81 6.79 6.78
4.18 4.16 4.14 4.12 3.81 3.72
1.31 1.29
OMe
Me
N
Me
Cl
3'i
7.45 1.18 1.09 2.98 1.15 3.00 2.91 3.15
8.5 8.0 7.5 7.0 6.5 6.0 5.5 5.0 4.5 4.0 3.5 3.0 2.5 2.0 1.5
ppm

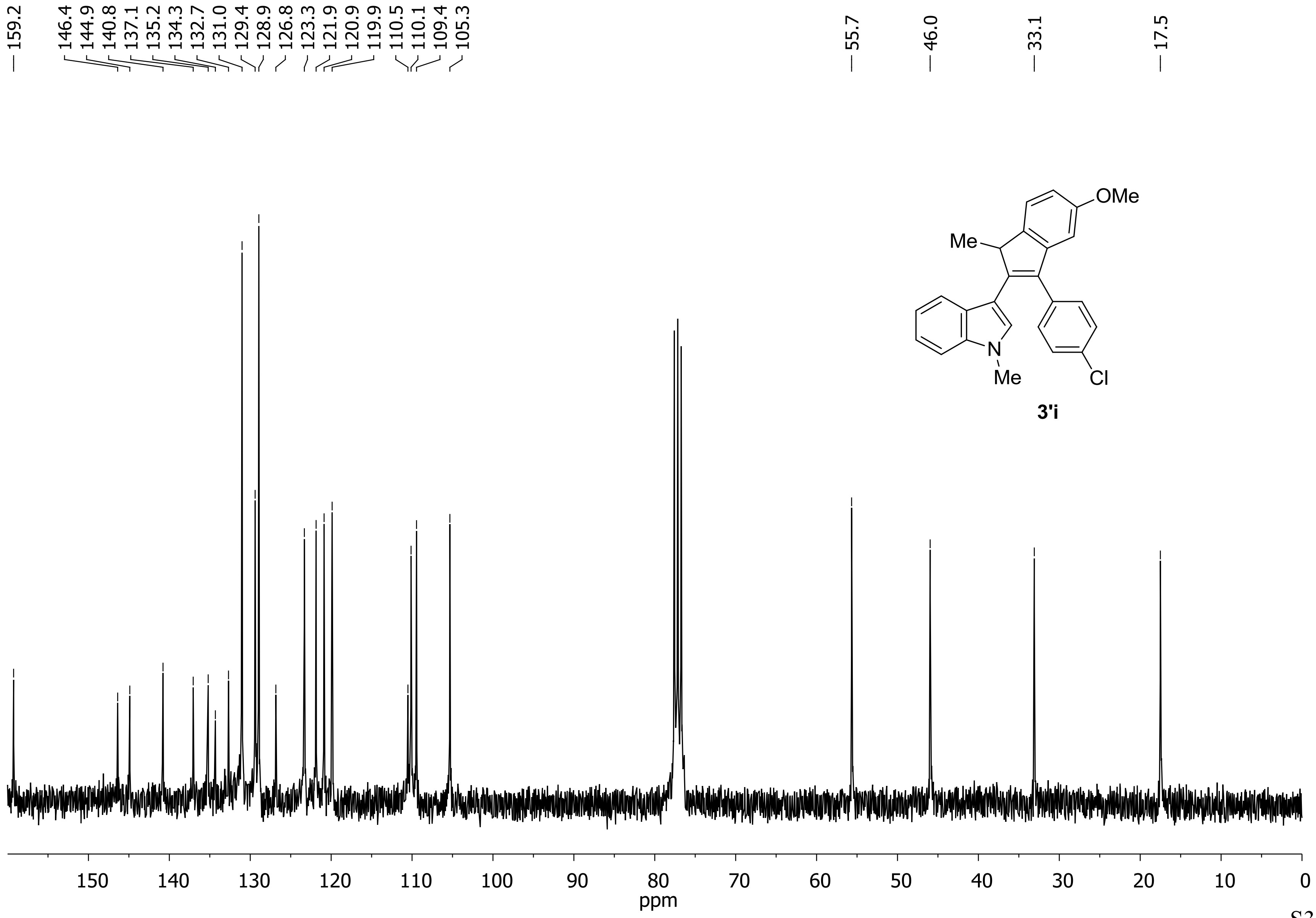

159.2
146.4
144.9
140.8
137.1
135.2
134.3
132.7
131.0
129.4
128.9
126.8
123.3
121.9
120.9
119.9
110.5
110.1
109.4
105.3
55.7
46.0
33.1
17.5
OMe
Me
N
Me
Cl
3'i
150
140
130
120
110
100
90
80
70
60
50
40
30
20
10
0
ppm

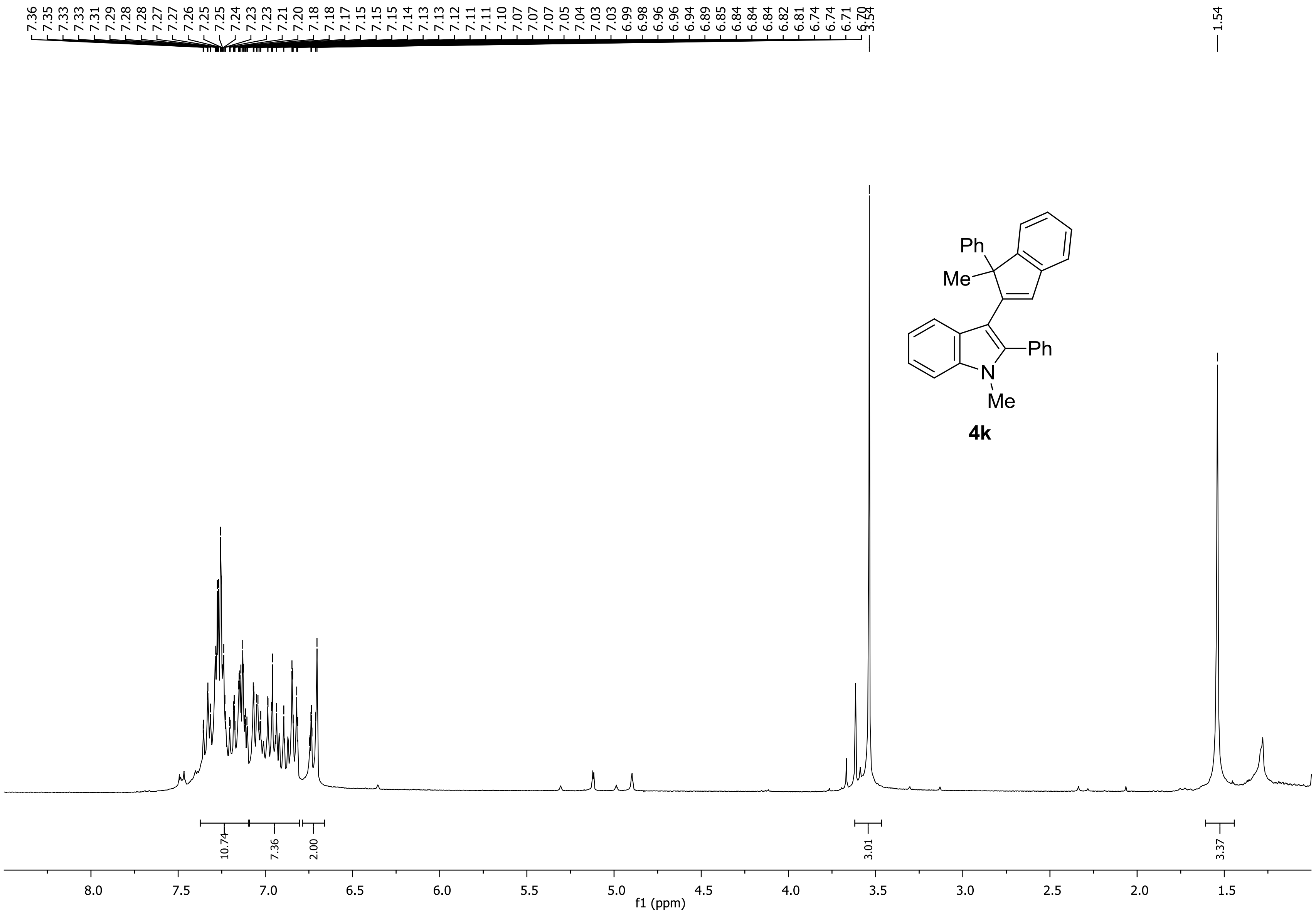
7.36
7.35
7.33
7.33
7.31
7.29
7.28
7.28
7.27
7.27
7.26
7.25
7.25
7.24
7.23
7.23
7.21
7.20
7.18
7.18
7.17
7.15
7.15
7.15
7.14
7.13
7.13
7.12
7.11
7.11
7.10
7.07
7.07
7.07
7.05
7.04
7.03
7.03
6.99
6.98
6.96
6.96
6.94
6.89
6.85
6.84
6.84
6.84
6.82
6.81
6.74
6.74
6.71
6.70
3.54
1.54
Ph
Me
Ph
N
Me
4k
10.74
7.36
2.00
3.01
3.37
8.0
7.5
7.0
6.5
6.0
5.5
5.0
4.5
4.0
3.5
3.0
2.5
2.0
1.5
f1 (ppm)

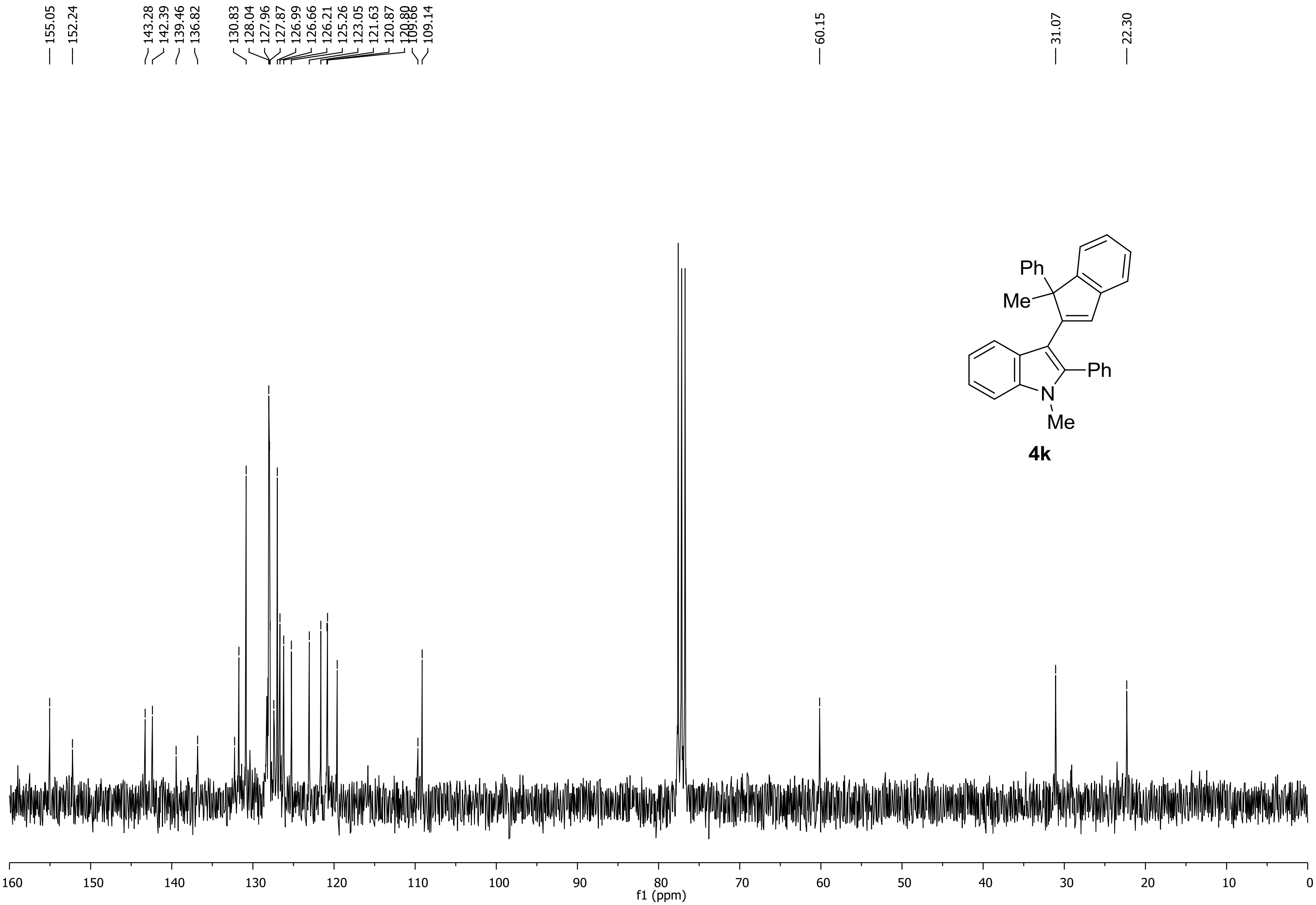
155.05
152.24
143.28
142.39
139.46
136.82
130.83
128.04
127.96
127.87
126.99
126.66
126.21
125.26
123.05
121.63
120.87
109.14
60.15
31.07
22.30
f1 (ppm)
Ph
Me
Ph
N
Me

4k

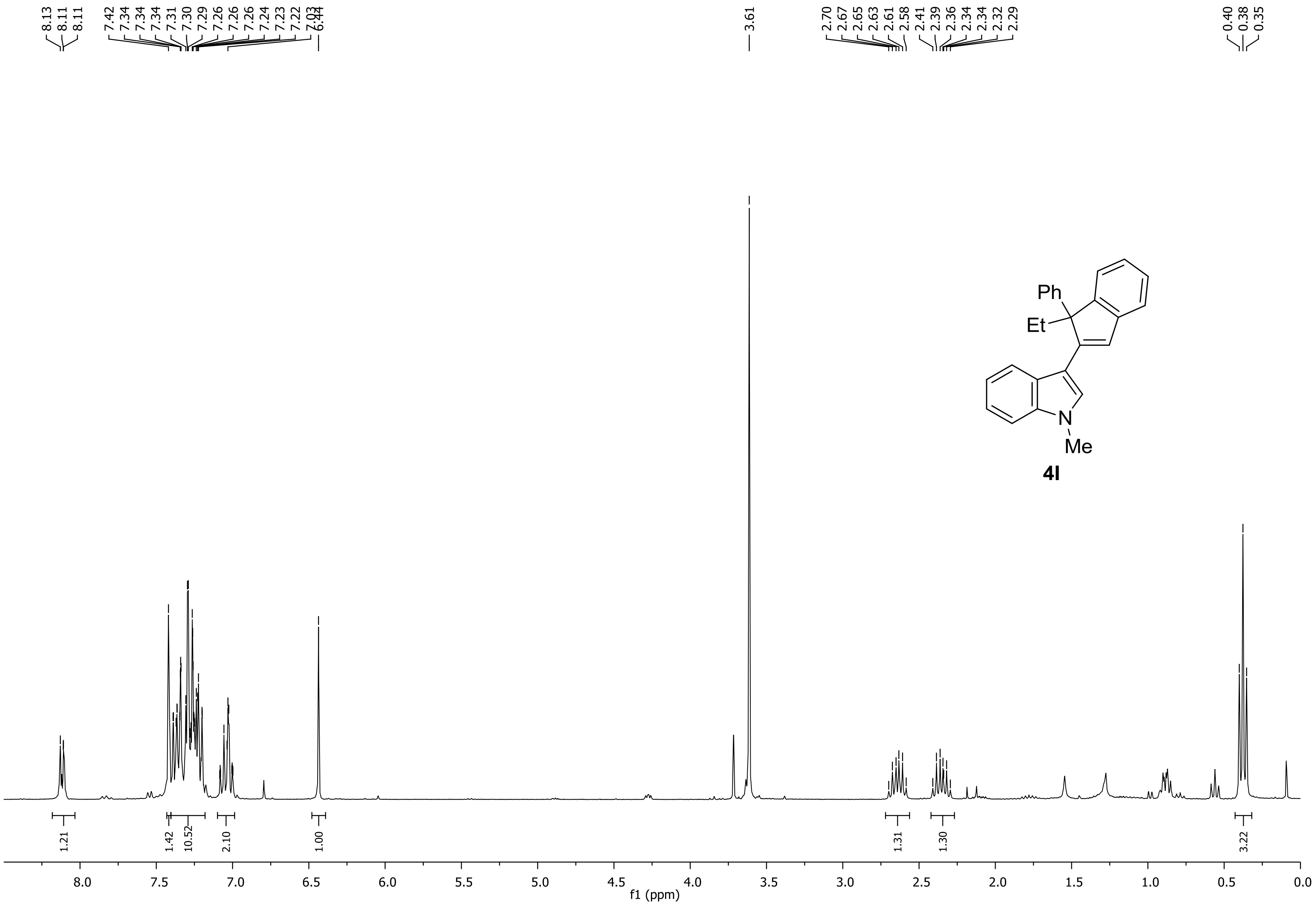
8.13
8.11
8.11
7.42
7.34
7.34
7.34
7.31
7.30
7.29
7.26
7.26
7.26
7.24
7.23
7.22
7.03
6.44
3.61
2.70
2.67
2.65
2.63
2.61
2.58
2.41
2.39
2.36
2.34
2.34
2.32
2.29
0.40
0.38
0.35
Ph
Et
N
Me
4l
1.21
1.42
10.52
2.10
1.00
1.31
1.30
3.22
8.0
7.5
7.0
6.5
6.0
5.5
5.0
4.5
4.0
3.5
3.0
2.5
2.0
1.5
1.0
0.5
0.0
f1 (ppm)

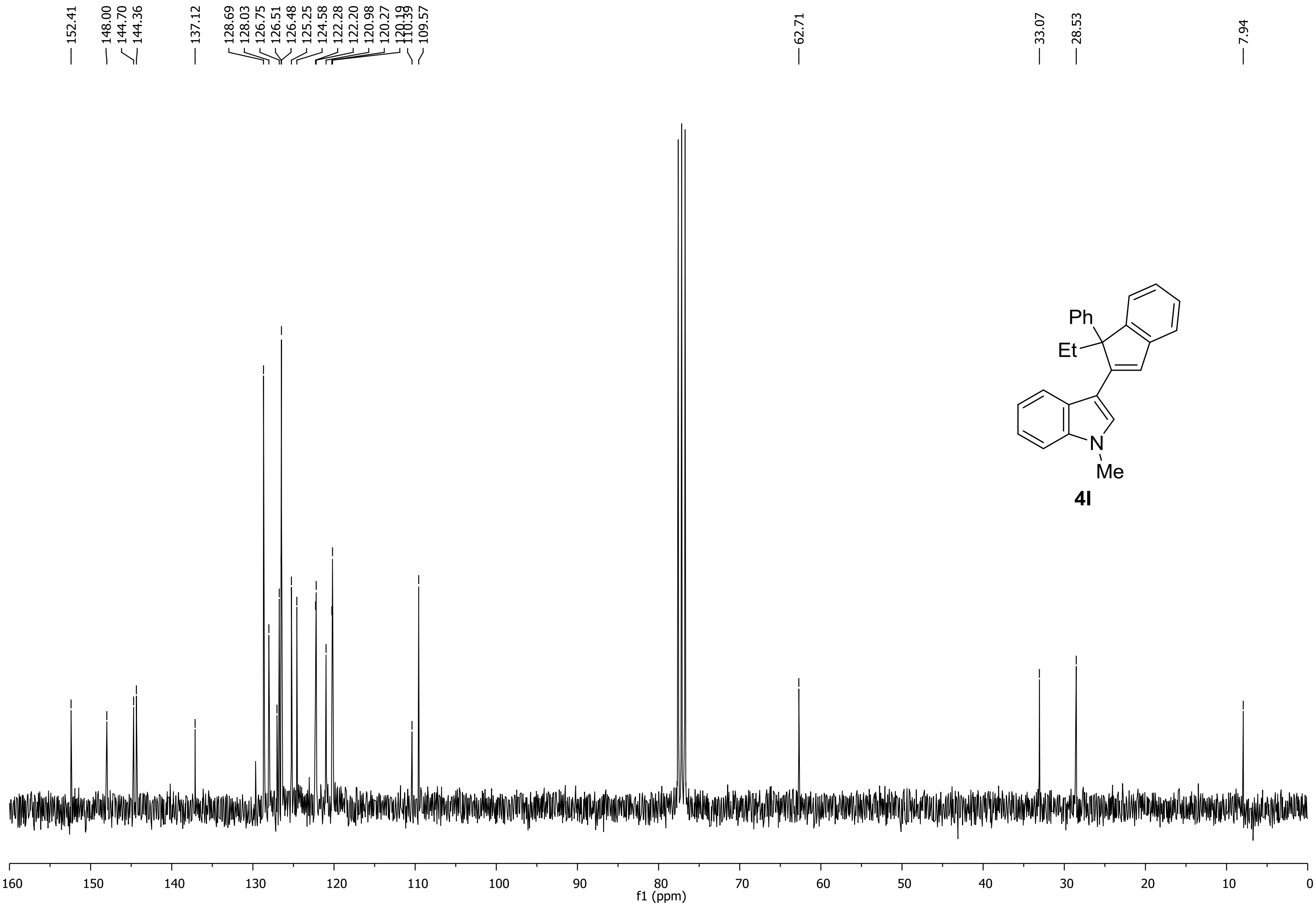

152.41
148.00
144.70
144.36
137.12
128.69
128.03
126.75
126.51
126.48
125.25
124.58
122.28
122.20
120.98
120.27
120.19
110.39
109.57
62.71
33.07
28.53
7.94
Ph
Et
N
Me
4l
160
150
140
130
120
110
100
90
80
70
60
50
40
30
20
10
0
f1 (ppm)

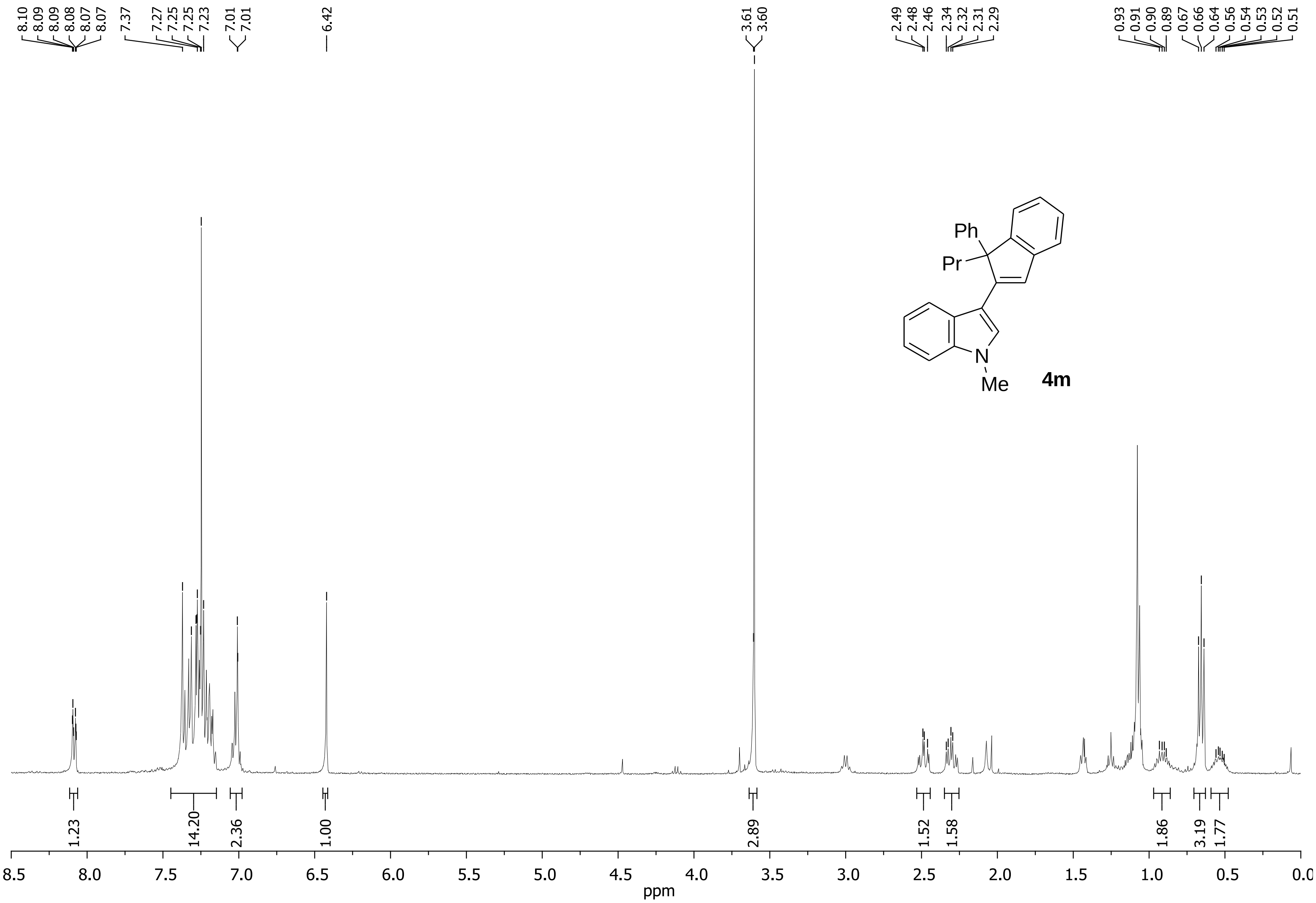
8.10 8.09 8.09 8.08 8.07 8.07 7.37 7.27 7.25 7.25 7.23 7.01 7.01 6.42 3.61 3.60 2.49 2.48 2.46 2.34 2.32 2.31 2.29 0.93 0.91 0.90 0.89 0.67 0.66 0.64 0.56 0.54 0.53 0.52 0.51
Ph
Pr
N
Me
4m
1.23 14.20 2.36 1.00 2.89 1.52 1.58 1.86 3.19 1.77
8.5 8.0 7.5 7.0 6.5 6.0 5.5 5.0 4.5 4.0 3.5 3.0 2.5 2.0 1.5 1.0 0.5 0.0
ppm

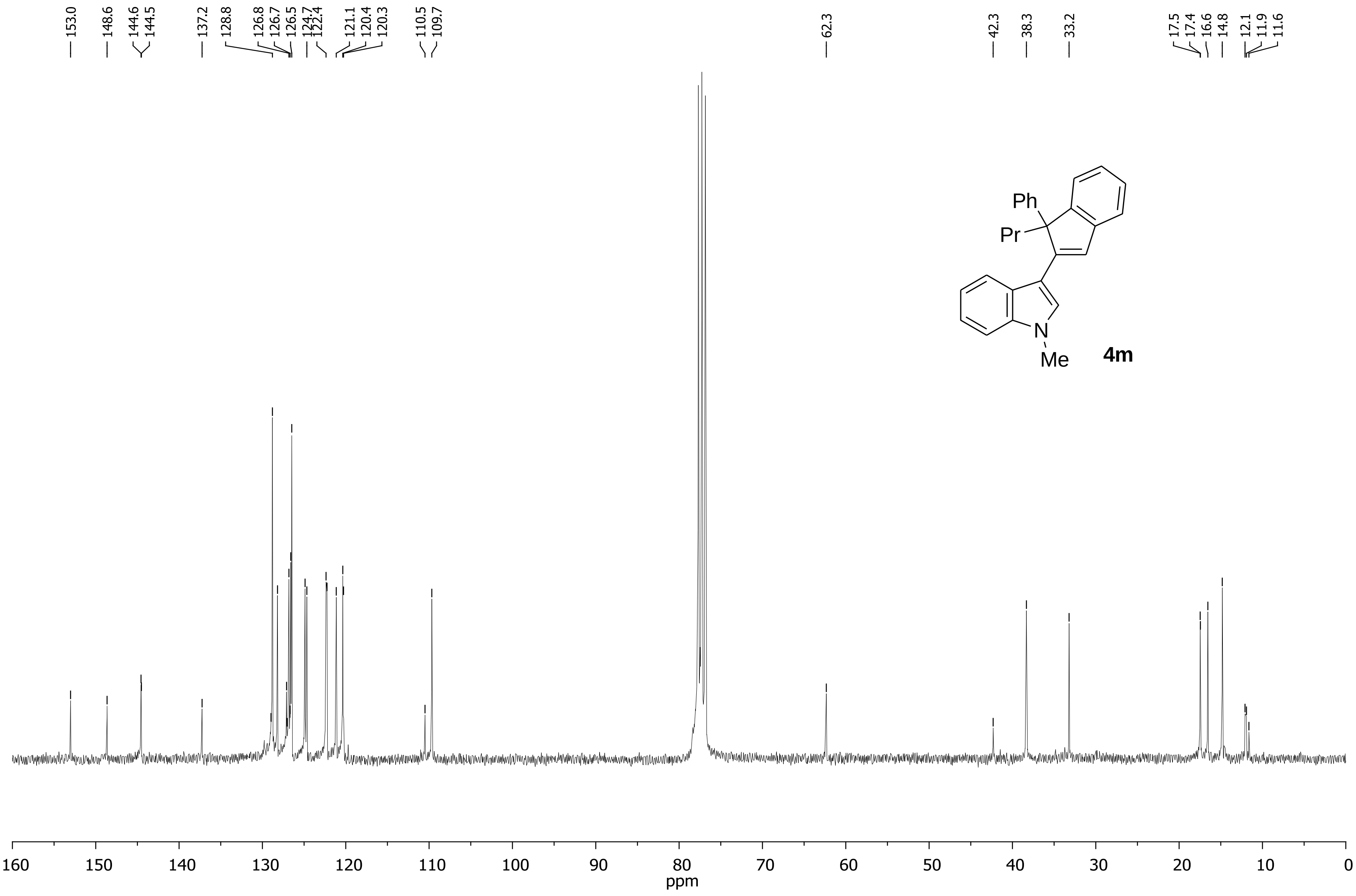
153.0
148.6
144.6
144.5
137.2
128.8
126.8
126.7
126.5
124.7
122.4
121.1
120.4
120.3
110.5
109.7
62.3
42.3
38.3
33.2
17.5
17.4
16.6
14.8
12.1
11.9
11.6
Ph
Pr
N
Me
160
150
140
130
120
110
100
90
80
70
60
50
40
30
20
10
0
ppm

4m
